# Supplementary material for: Complete chloroplast genome studies of different apple varieties indicated the origin of modern cultivated apples from Malus sieversii and Malus sylvestris
Source: PeerJ. 2022 Mar 18;10:e13107. doi: 10.7717/peerj.13107 (PMC8935992; doi:10.7717/peerj.13107)
Supplement: Supplemental Information 1 — WW: Wunan Town, Wuwei City; LX: Xingcheng, Liaoning; ZZ: Zhengzhou Fruit Tree Institute [file peerj-10-13107-s001.docx]

|  |  | *Red Delicious* | | | | | *Golden Delicious* | | | *Ralls* | | Red *Red Fuji* | | | *Malus sieversii* | *Malus sylvestris* |
| --- | --- | --- | --- | --- | --- | --- | --- | --- | --- | --- | --- | --- | --- | --- | --- | --- |
|  |  | WW | LX | | ZZ | | WW | LX | ZZ | LX | ZZ | WW | LX | ZZ |  |  |
| LSC(%) | A | 32.3 | | 32.3 | | 32.3 | 32.3 | 32.3 | 32.2 | 32.2 | 32.2 | 32.2 | 32.2 | 32.2 | 32.2 | 32.2 |
|  | C | 17.6 | | 17.6 | | 17.6 | 17.6 | 17.6 | 17.6 | 17.6 | 17.6 | 17.6 | 17.6 | 17.6 | 17.6 | 17.6 |
|  | G | 16.6 | | 16.6 | | 16.6 | 16.6 | 16.6 | 16.6 | 16.6 | 16.6 | 16.6 | 16.6 | 16.6 | 16.6 | 16.6 |
|  | T | 33.6 | | 33.6 | | 33.6 | 33.6 | 33.6 | 33.6 | 33.6 | 33.6 | 33.6 | 33.6 | 33.6 | 33.6 | 33.6 |
|  | GC | 34.1 | | 34.1 | | 34.1 | 34.1 | 34.2 | 34.1 | 34.2 | 34.2 | 34.2 | 34.2 | 34.2 | 34.1 | 34.2 |
| SSC(%) | A | 34.8 | | 34.8 | | 34.8 | 31.4 | 34.7 | 34.7 | 34.7 | 34.1 | 34.7 | 34.7 | 34.8 | 34.8 | 34.8 |
|  | C | 16.0 | | 14.5 | | 16.0 | 18.2 | 15.9 | 15.9 | 15.9 | 15.9 | 15.9 | 15.9 | 14.5 | 16.0 | 15.9 |
|  | G | 14.5 | | 16.0 | | 14.5 | 19.1 | 14.5 | 14.5 | 14.5 | 14.5 | 14.5 | 14.5 | 15.9 | 14.5 | 14.5 |
|  | T | 34.8 | | 34.8 | | 34.8 | 31.3 | 34.8 | 34.8 | 34.8 | 34.8 | 34.8 | 34.8 | 34.7 | 34.8 | 34.8 |
|  | GC | 30.4 | | 30.4 | | 30.4 | 37.3 | 30.4 | 30.4 | 30.4 | 30.4 | 30.4 | 30.4 | 30.4 | 30.4 | 30.4 |
| IR (%) | A | 28.8 | | 28.7 | | 28.8 | 28.9 | 28.8 | 28.8 | 28.8 | 28.7 | 28.8 | 28.7 | 28.7 | 28.6 | 28.6 |
|  | C | 20.6 | | 20.6 | | 20.6 | 20.1 | 20.6 | 20.6 | 20.6 | 20.6 | 20.6 | 20.6 | 20.6 | 20.6 | 20.6 |
|  | G | 22.1 | | 22.1 | | 22.1 | 21.0 | 22.1 | 22.1 | 22.1 | 22.1 | 22.1 | 22.1 | 22.1 | 22.1 | 22.1 |
|  | T | 28.6 | | 28.6 | | 28.6 | 30.0 | 28.5 | 28.5 | 28.5 | 28.6 | 28.5 | 28.6 | 28.6 | 28.7 | 28.7 |
|  | GC | 42.7 | | 42.7 | | 42.7 | 41.0 | 42.7 | 42.7 | 42.7 | 42.7 | 42.7 | 42.7 | 42.7 | 42.7 | 42.7 |
| Total(%) | A | 31.4 | | 31.4 | | 31.4 | 31.4 | 31.3 | 31.3 | 31.3 | 31.3 | 31.3 | 31.3 | 31.3 | 31.4 | 31.3 |
|  | C | 18.6 | | 18.4 | | 18.6 | 18.4 | 18.6 | 18.6 | 18.6 | 18.6 | 18.6 | 18.6 | 18.5 | 18.6 | 18.6 |
|  | G | 17.9 | | 18.1 | | 17.9 | 18.1 | 17.9 | 17.9 | 17.9 | 17.9 | 17.9 | 17.9 | 18.1 | 17.9 | 17.9 |
|  | T | 32.1 | | 32.1 | | 32.1 | 32.1 | 32.1 | 32.1 | 32.1 | 32.1 | 32.1 | 32.1 | 32.1 | 32.1 | 32.1 |
|  | GC | 36.5 | | 36.5 | | 36.5 | 36.5 | 36.6 | 36.6 | 36.6 | 36.6 | 36.6 | 36.6 | 36.6 | 36.5 | 36.6 |
