# Supplementary material for: Complete chloroplast genome studies of different apple varieties indicated the origin of modern cultivated apples from Malus sieversii and Malus sylvestris
Source: PeerJ. 2022 Mar 18;10:e13107. doi: 10.7717/peerj.13107 (PMC8935992; doi:10.7717/peerj.13107)
Supplement: Supplemental Information 2 — P1: mono-nucleotide; P2: di-nucleotide; C: hexa-nucleotide; LSC: Number of SSR loci in the LSC region; SSC: Number of SSR loci in the SSC region; IR: Number of SSR loci in the IR region. [file peerj-10-13107-s002.docx]

|  | *Red*  *Delicious* | | | *Golden*  *Delicious* | | | *Ralls* | | *Red Fuji* | | | *Malus sieversii* | *Malus sylvestris* |
| --- | --- | --- | --- | --- | --- | --- | --- | --- | --- | --- | --- | --- | --- |
| SSR | 62 | 62 | 62 | 62 | 58 | 57 | 57 | 58 | 57 | 58 | 57 | 58 | 65 |
| P1 | 50 | 50 | 50 | 50 | 47 | 47 | 47 | 47 | 46 | 47 | 47 | 47 | 51 |
| P2 | 3 | 3 | 3 | 3 | 2 | 2 | 2 | 2 | 2 | 2 | 2 | 2 | 3 |
| C | 9 | 9 | 9 | 9 | 9 | 8 | 8 | 9 | 9 | 9 | 8 | 9 | 11 |
| LSC | 50 | 50 | 50 | 49 | 47 | 46 | 46 | 47 | 46 | 47 | 46 | 52 | 47 |
| SSC | 8 | 8 | 8 | 9 | 7 | 7 | 7 | 7 | 7 | 7 | 7 | 9 | 7 |
| IR | 4 | 4 | 4 | 4 | 4 | 4 | 4 | 4 | 4 | 4 | 4 | 4 | 4 |
