# Supplementary material for: Complete chloroplast genome studies of different apple varieties indicated the origin of modern cultivated apples from Malus sieversii and Malus sylvestris
Source: PeerJ. 2022 Mar 18;10:e13107. doi: 10.7717/peerj.13107 (PMC8935992; doi:10.7717/peerj.13107)
Supplement: Supplemental Information 3 [file peerj-10-13107-s003.docx]

| Red Delicious | | | | | | |
| --- | --- | --- | --- | --- | --- | --- |
| SSR nr. | SSR type | SSR | size | start | end | region |
| 1 | c | (T)10act(A)15 | 28 | 261 | 288 | LSC |
| 2 | p1 | (T)11 | 11 | 1699 | 1709 | LSC |
| 3 | p1 | (T)11 | 11 | 2852 | 2862 | LSC |
| 4 | p1 | (C)13 | 13 | 5700 | 5712 | LSC |
| 5 | p1 | (A)10 | 10 | 6881 | 6890 | LSC |
| 6 | p1 | (A)14 | 14 | 7037 | 7050 | LSC |
| 7 | p1 | (A)12 | 12 | 7911 | 7922 | LSC |
| 8 | p1 | (T)10 | 10 | 8618 | 8627 | LSC |
| 9 | p1 | (T)11 | 11 | 9342 | 9352 | LSC |
| 10 | p1 | (T)15 | 15 | 13257 | 13271 | LSC |
| 11 | p1 | (T)10 | 10 | 13612 | 13621 | LSC |
| 12 | p1 | (A)11 | 11 | 14583 | 14593 | LSC |
| 13 | p1 | (A)12 | 12 | 15330 | 15341 | LSC |
| 14 | p1 | (T)12 | 12 | 15585 | 15596 | LSC |
| 15 | p1 | (T)14 | 14 | 17427 | 17440 | LSC |
| 16 | p1 | (T)11 | 11 | 19674 | 19684 | LSC |
| 17 | p1 | (T)10 | 10 | 27376 | 27385 | LSC |
| 18 | p1 | (T)12 | 12 | 28021 | 28032 | LSC |
| 19 | p1 | (A)13 | 13 | 28454 | 28466 | LSC |
| 20 | p1 | (A)11 | 11 | 28895 | 28905 | LSC |
| 21 | p1 | (A)13 | 13 | 32310 | 32322 | LSC |
| 22 | p1 | (T)10 | 10 | 33233 | 33242 | LSC |
| 23 | c | (A)17(T)13 | 61 | 38966 | 39026 | LSC |
| 24 | p1 | (A)10 | 10 | 39636 | 39645 | LSC |
| 25 | p1 | (A)10 | 10 | 39977 | 39986 | LSC |
| 26 | p1 | (A)11 | 11 | 45603 | 45613 | LSC |
| 27 | p1 | (A)10 | 10 | 47953 | 47962 | LSC |
| 28 | p1 | (A)13 | 13 | 50654 | 50666 | LSC |
| 29 | c | (T)11(A)18 | 55 | 52639 | 52693 | LSC |
| 30 | p1 | (T)10 | 10 | 58148 | 58157 | LSC |
| 31 | p1 | (T)17 | 17 | 60857 | 60873 | LSC |
| 32 | c | (T)19(T)15 | 61 | 67244 | 67304 | LSC |
| 33 | p1 | (T)17 | 17 | 69019 | 69035 | LSC |
| 34 | p1 | (A)12 | 12 | 69348 | 69359 | LSC |
| 35 | p2 | (AT)6 | 12 | 70437 | 70448 | LSC |
| 36 | p1 | (A)17 | 17 | 71290 | 71306 | LSC |
| 37 | p2 | (AT)6 | 12 | 71909 | 71920 | LSC |
| 38 | p1 | (T)10 | 10 | 72433 | 72442 | LSC |
| 39 | p1 | (T)16 | 16 | 73151 | 73166 | LSC |
| 40 | p1 | (T)11 | 11 | 74580 | 74590 | LSC |
| 41 | c | (A)12(T)13 | 68 | 75259 | 75326 | LSC |
| 42 | p1 | (A)10 | 10 | 79658 | 79667 | LSC |
| 43 | p1 | (A)10 | 10 | 80545 | 80554 | LSC |
| 44 | p1 | (A)12 | 12 | 82045 | 82056 | LSC |
| 45 | p1 | (T)17 | 17 | 83638 | 83654 | LSC |
| 46 | c | (T)10(A)12 | 57 | 85305 | 85361 | LSC |
| 47 | p1 | (T)16 | 16 | 86638 | 86653 | LSC |
| 48 | p1 | (T)10 | 10 | 86894 | 86903 | LSC |
| 49 | p1 | (T)10 | 10 | 87693 | 87702 | LSC |
| 50 | p1 | (T)10 | 10 | 104446 | 104455 | IRb |
| 51 | p1 | (A)10 | 10 | 112440 | 112449 | IRb |
| 52 | c | (A)19(A)10 | 97 | 117038 | 117134 | SSC |
| 53 | c* | (T)14(C)10(T)10* | 99 | 117876 | 117974 | SSC |
| 54 | p1 | (T)10 | 10 | 118711 | 118720 | SSC |
| 55 | p2 | (AT)6 | 12 | 118974 | 118985 | SSC |
| 56 | p1 | (T)10 | 10 | 124306 | 124315 | SSC |
| 57 | c | (A)10(T)17 | 122 | 126114 | 126235 | SSC |
| 58 | p1 | (A)11 | 11 | 126781 | 126791 | SSC |
| 59 | p1 | (T)10 | 10 | 132131 | 132140 | SSC |
| 61 | p1 | (T)10 | 10 | 136312 | 136321 | IRa |
| 62 | p1 | (A)10 | 10 | 144306 | 144315 | IRa |

| Ralls | | | | | | |
| --- | --- | --- | --- | --- | --- | --- |
| SSR nr. | SSR type | SSR | size | start | end | region |
| 1 | c | (T)10act(A)13 | 26 | 241 | 266 | LSC |
| 2 | p1 | (T)11 | 11 | 2805 | 2815 | LSC |
| 3 | p1 | (A)10 | 10 | 5684 | 5693 | LSC |
| 4 | p1 | (A)11 | 11 | 6853 | 6863 | LSC |
| 5 | p1 | (A)12 | 12 | 7010 | 7021 | LSC |
| 6 | p1 | (A)13 | 13 | 7898 | 7910 | LSC |
| 7 | p1 | (T)10 | 10 | 9329 | 9338 | LSC |
| 8 | p1 | (T)10 | 10 | 12528 | 12537 | LSC |
| 9 | p1 | (T)16 | 16 | 13247 | 13262 | LSC |
| 10 | p1 | (A)12 | 12 | 14573 | 14584 | LSC |
| 11 | p1 | (C)12 | 12 | 15108 | 15119 | LSC |
| 12 | p1 | (A)14 | 14 | 15324 | 15337 | LSC |
| 13 | p1 | (T)12 | 12 | 15581 | 15592 | LSC |
| 14 | p1 | (T)13 | 13 | 17423 | 17435 | LSC |
| 15 | p1 | (T)11 | 11 | 19668 | 19678 | LSC |
| 16 | p1 | (T)10 | 10 | 27370 | 27379 | LSC |
| 17 | p1 | (T)11 | 11 | 28015 | 28025 | LSC |
| 18 | p1 | (A)13 | 13 | 28447 | 28459 | LSC |
| 19 | p1 | (A)12 | 12 | 28868 | 28879 | LSC |
| 20 | p1 | (A)10 | 10 | 32274 | 32283 | LSC |
| 21 | p1 | (T)10 | 10 | 33195 | 33204 | LSC |
| 22 | c | (A)17(T)16 | 64 | 38681 | 38744 | LSC |
| 23 | p1 | (A)10 | 10 | 39381 | 39390 | LSC |
| 24 | p1 | (A)10 | 10 | 39746 | 39755 | LSC |
| 25 | p1 | (A)11 | 11 | 47721 | 47731 | LSC |
| 26 | p1 | (A)13 | 13 | 50452 | 50464 | LSC |
| 27 | c | (T)11(A)17 | 60 | 52413 | 52472 | LSC |
| 28 | p1 | (T)10 | 10 | 53886 | 53895 | LSC |
| 29 | p1 | (T)10 | 10 | 57920 | 57929 | LSC |
| 30 | p1 | (T)19 | 19 | 60635 | 60653 | LSC |
| 31 | c | (T)15(T)14 | 56 | 67007 | 67062 | LSC |
| 32 | p1 | (T)16 | 16 | 68777 | 68792 | LSC |
| 33 | p1 | (A)12 | 12 | 69105 | 69116 | LSC |
| 34 | p1 | (A)16 | 16 | 71044 | 71059 | LSC |
| 35 | p2 | (AT)6 | 12 | 71662 | 71673 | LSC |
| 36 | p1 | (T)12 | 12 | 72205 | 72216 | LSC |
| 37 | p1 | (T)12 | 12 | 72925 | 72936 | LSC |
| 38 | p1 | (T)14 | 14 | 74396 | 74409 | LSC |
| 39 | c | (A)12(T)16 | 67 | 75078 | 75144 | LSC |
| 40 | p1 | (A)11 | 11 | 80362 | 80372 | LSC |
| 41 | p1 | (A)11 | 11 | 81863 | 81873 | LSC |
| 42 | p1 | (T)17 | 17 | 83451 | 83467 | LSC |
| 43 | p1 | (T)10 | 10 | 84620 | 84629 | LSC |
| 44 | c | (T)10(A)13 | 53 | 85107 | 85159 | LSC |
| 45 | p1 | (T)19 | 19 | 86436 | 86454 | LSC |
| 46 | p1 | (T)10 | 10 | 87493 | 87502 | LSC |
| 47 | p1 | (T)10 | 10 | 104237 | 104246 | IRb |
| 48 | p1 | (A)10 | 10 | 112226 | 112235 | IRb |
| 49 | c | (A)23(A)10 | 100 | 116824 | 116923 | SSC |
| 50 | c | (T)15(T)11 | 100 | 117665 | 117764 | SSC |
| 51 | p2 | (AT)6 | 12 | 118748 | 118759 | SSC |
| 52 | p1 | (T)26 | 26 | 126004 | 126029 | SSC |
| 53 | p1 | (A)10 | 10 | 126575 | 126584 | SSC |
| 54 | p1 | (T)10 | 10 | 131924 | 131933 | SSC |
| 55 | p1 | (A)16 | 16 | 132555 | 132570 | SSC |
| 56 | p1 | (T)10 | 10 | 136105 | 136114 | IRa |
| 57 | p1 | (A)10 | 10 | 144094 | 144103 | IRa |

| Golden Delicious | | | | | | |
| --- | --- | --- | --- | --- | --- | --- |
| SSR nr. | SSR type | SSR | size | start | end | region |
| 1 | c | (T)10act(A)13 | 26 | 241 | 266 | LSC |
| 2 | p1 | (T)11 | 11 | 2805 | 2815 | LSC |
| 3 | p1 | (C)10 | 10 | 5520 | 5529 | LSC |
| 4 | c* | (C)10(A)10* | 20 | 5676 | 5695 | LSC |
| 5 | p1 | (A)11 | 11 | 6855 | 6865 | LSC |
| 6 | p1 | (A)12 | 12 | 7012 | 7023 | LSC |
| 7 | p1 | (A)13 | 13 | 7900 | 7912 | LSC |
| 8 | p1 | (T)10 | 10 | 9331 | 9340 | LSC |
| 9 | p1 | (T)10 | 10 | 12530 | 12539 | LSC |
| 10 | p1 | (T)16 | 16 | 13249 | 13264 | LSC |
| 11 | p1 | (A)12 | 12 | 14575 | 14586 | LSC |
| 12 | p1 | (C)12 | 12 | 15110 | 15121 | LSC |
| 13 | p1 | (A)14 | 14 | 15326 | 15339 | LSC |
| 14 | p1 | (T)12 | 12 | 15583 | 15594 | LSC |
| 15 | p1 | (T)13 | 13 | 17425 | 17437 | LSC |
| 16 | p1 | (T)11 | 11 | 19670 | 19680 | LSC |
| 17 | p1 | (T)10 | 10 | 27372 | 27381 | LSC |
| 18 | p1 | (T)11 | 11 | 28017 | 28027 | LSC |
| 19 | p1 | (A)13 | 13 | 28449 | 28461 | LSC |
| 20 | p1 | (A)12 | 12 | 28870 | 28881 | LSC |
| 21 | p1 | (A)10 | 10 | 32276 | 32285 | LSC |
| 22 | p1 | (T)10 | 10 | 33197 | 33206 | LSC |
| 23 | c | (A)17t(T)16 | 64 | 38683 | 38746 | LSC |
| 24 | p1 | (A)10 | 10 | 39383 | 39392 | LSC |
| 25 | p1 | (A)10 | 10 | 39748 | 39757 | LSC |
| 26 | p1 | (A)11 | 11 | 47723 | 47733 | LSC |
| 27 | p1 | (A)13 | 13 | 50454 | 50466 | LSC |
| 28 | c | (T)11(A)17 | 60 | 52415 | 52474 | LSC |
| 29 | p1 | (T)10 | 10 | 53888 | 53897 | LSC |
| 30 | p1 | (T)10 | 10 | 57922 | 57931 | LSC |
| 31 | p1 | (T)19 | 19 | 60637 | 60655 | LSC |
| 32 | c | (T)15(T)14 | 56 | 67009 | 67064 | LSC |
| 33 | p1 | (T)16 | 16 | 68779 | 68794 | LSC |
| 34 | p1 | (A)12 | 12 | 69107 | 69118 | LSC |
| 35 | p1 | (A)16 | 16 | 71046 | 71061 | LSC |
| 36 | p2 | (AT)6 | 12 | 71664 | 71675 | LSC |
| 37 | p1 | (T)12 | 12 | 72207 | 72218 | LSC |
| 38 | p1 | (T)12 | 12 | 72927 | 72938 | LSC |
| 39 | p1 | (T)14 | 14 | 74398 | 74411 | LSC |
| 40 | c | (A)12(T)16 | 67 | 75080 | 75146 | LSC |
| 41 | p1 | (A)11 | 11 | 80364 | 80374 | LSC |
| 42 | p1 | (A)11 | 11 | 81865 | 81875 | LSC |
| 43 | p1 | (T)17 | 17 | 83453 | 83469 | LSC |
| 44 | p1 | (T)10 | 10 | 84622 | 84631 | LSC |
| 45 | c | (T)10(A)13 | 53 | 85109 | 85161 | LSC |
| 46 | p1 | (T)19 | 19 | 86438 | 86456 | LSC |
| 47 | p1 | (T)10 | 10 | 87495 | 87504 | LSC |
| 48 | p1 | (T)10 | 10 | 104239 | 104248 | IRb |
| 49 | p1 | (A)10 | 10 | 112228 | 112237 | IRb |
| 50 | c | (A)22(A)10 | 100 | 116826 | 116925 | SSC |
| 51 | c | (T)15(T)11 | 100 | 117667 | 117766 | SSC |
| 52 | p2 | (AT)6 | 12 | 118750 | 118761 | SSC |
| 53 | p1 | (T)25 | 25 | 126006 | 126030 | SSC |
| 54 | p1 | (A)10 | 10 | 126576 | 126585 | SSC |
| 55 | p1 | (T)10 | 10 | 131925 | 131934 | SSC |
| 56 | p1 | (A)16 | 16 | 132556 | 132571 | SSC |
| 57 | p1 | (T)10 | 10 | 136106 | 136115 | IRa |
| 58 | p1 | (A)10 | 10 | 144095 | 144104 | IRa |

| Red Fuji | | | | | | |
| --- | --- | --- | --- | --- | --- | --- |
| SSR nr. | SSR type | SSR | size | start | end | region |
| 1 | c | (T)10act(A)13 | 26 | 241 | 266 | LSC |
| 2 | p1 | (T)11 | 11 | 2805 | 2815 | LSC |
| 3 | c* | (C)10(A)10* | 20 | 5675 | 5694 | LSC |
| 4 | p1 | (A)11 | 11 | 6854 | 6864 | LSC |
| 5 | p1 | (A)12 | 12 | 7011 | 7022 | LSC |
| 6 | p1 | (A)13 | 13 | 7899 | 7911 | LSC |
| 7 | p1 | (T)10 | 10 | 9330 | 9339 | LSC |
| 8 | p1 | (T)10 | 10 | 12529 | 12538 | LSC |
| 9 | p1 | (T)16 | 16 | 13248 | 13263 | LSC |
| 10 | p1 | (A)12 | 12 | 14574 | 14585 | LSC |
| 11 | p1 | (C)12 | 12 | 15109 | 15120 | LSC |
| 12 | p1 | (A)14 | 14 | 15325 | 15338 | LSC |
| 13 | p1 | (T)12 | 12 | 15582 | 15593 | LSC |
| 14 | p1 | (T)13 | 13 | 17424 | 17436 | LSC |
| 15 | p1 | (T)11 | 11 | 19669 | 19679 | LSC |
| 16 | p1 | (T)10 | 10 | 27371 | 27380 | LSC |
| 17 | p1 | (T)11 | 11 | 28016 | 28026 | LSC |
| 18 | p1 | (A)13 | 13 | 28448 | 28460 | LSC |
| 19 | p1 | (A)12 | 12 | 28869 | 28880 | LSC |
| 20 | p1 | (A)10 | 10 | 32275 | 32284 | LSC |
| 21 | p1 | (T)10 | 10 | 33196 | 33205 | LSC |
| 22 | c | (A)17(T)16 | 64 | 38682 | 38745 | LSC |
| 23 | p1 | (A)10 | 10 | 39382 | 39391 | LSC |
| 24 | p1 | (A)10 | 10 | 39747 | 39756 | LSC |
| 25 | p1 | (A)11 | 11 | 47722 | 47732 | LSC |
| 26 | p1 | (A)13 | 13 | 50453 | 50465 | LSC |
| 27 | c | (T)11(A)17 | 60 | 52414 | 52473 | LSC |
| 28 | p1 | (T)10 | 10 | 53887 | 53896 | LSC |
| 29 | p1 | (T)10 | 10 | 57921 | 57930 | LSC |
| 30 | p1 | (T)19 | 19 | 60636 | 60654 | LSC |
| 31 | c | (T)15(T)14 | 56 | 67008 | 67063 | LSC |
| 32 | p1 | (T)16 | 16 | 68778 | 68793 | LSC |
| 33 | p1 | (A)12 | 12 | 69106 | 69117 | LSC |
| 34 | p1 | (A)16 | 16 | 71045 | 71060 | LSC |
| 35 | p2 | (AT)6 | 12 | 71663 | 71674 | LSC |
| 36 | p1 | (T)12 | 12 | 72206 | 72217 | LSC |
| 37 | p1 | (T)12 | 12 | 72926 | 72937 | LSC |
| 38 | p1 | (T)14 | 14 | 74397 | 74410 | LSC |
| 39 | c | (A)12(T)16 | 67 | 75079 | 75145 | LSC |
| 40 | p1 | (A)11 | 11 | 80363 | 80373 | LSC |
| 41 | p1 | (A)11 | 11 | 81864 | 81874 | LSC |
| 42 | p1 | (T)17 | 17 | 83452 | 83468 | LSC |
| 43 | p1 | (T)10 | 10 | 84621 | 84630 | LSC |
| 44 | c | (T)10(A)13 | 53 | 85108 | 85160 | LSC |
| 45 | p1 | (T)19 | 19 | 86437 | 86455 | LSC |
| 46 | p1 | (T)10 | 10 | 87494 | 87503 | LSC |
| 47 | p1 | (T)10 | 10 | 104238 | 104247 | IRb |
| 48 | p1 | (A)10 | 10 | 112227 | 112236 | IRb |
| 49 | c | (A)(A)10 | 100 | 116825 | 116924 | SSC |
| 50 | c | (T)15(T)11 | 100 | 117666 | 117765 | SSC |
| 51 | p2 | (AT)6 | 12 | 118749 | 118760 | SSC |
| 52 | p1 | (T)26 | 26 | 126005 | 126030 | SSC |
| 53 | p1 | (A)10 | 10 | 126576 | 126585 | SSC |
| 54 | p1 | (T)10 | 10 | 131925 | 131934 | SSC |
| 55 | p1 | (A)16 | 16 | 132556 | 132571 | SSC |
| 56 | p1 | (T)10 | 10 | 136106 | 136115 | IRa |
| 57 | p1 | (A)10 | 10 | 144095 | 144104 | IRa |

| *M. sylvestris* | | | | | | |
| --- | --- | --- | --- | --- | --- | --- |
| SSR nr. | SSR type | SSR | size | start | end | region |
| 1 | c | (T)10act(A)13 | 26 | 196 | 221 | LSC |
| 2 | p1 | (T)11 | 11 | 2760 | 2770 | LSC |
| 3 | p1 | (C)10 | 10 | 5475 | 5484 | LSC |
| 4 | c | (C)10(A)10 | 20 | 5631 | 5650 | LSC |
| 5 | p1 | (A)11 | 11 | 6810 | 6820 | LSC |
| 6 | p1 | (A)12 | 12 | 6967 | 6978 | LSC |
| 7 | p1 | (A)13 | 13 | 7855 | 7867 | LSC |
| 8 | p1 | (T)10 | 10 | 9286 | 9295 | LSC |
| 9 | p1 | (T)10 | 10 | 12485 | 12494 | LSC |
| 10 | p1 | (T)16 | 16 | 13204 | 13219 | LSC |
| 11 | p1 | (A)12 | 12 | 14530 | 14541 | LSC |
| 12 | p1 | (C)12 | 12 | 15065 | 15076 | LSC |
| 13 | p1 | (A)14 | 14 | 15281 | 15294 | LSC |
| 14 | p1 | (T)12 | 12 | 15538 | 15549 | LSC |
| 15 | p1 | (T)13 | 13 | 17380 | 17392 | LSC |
| 16 | p1 | (T)11 | 11 | 19625 | 19635 | LSC |
| 17 | p1 | (T)10 | 10 | 27327 | 27336 | LSC |
| 18 | p1 | (T)11 | 11 | 27972 | 27982 | LSC |
| 19 | p1 | (A)13 | 13 | 28404 | 28416 | LSC |
| 20 | p1 | (A)12 | 12 | 28825 | 28836 | LSC |
| 21 | p1 | (A)10 | 10 | 32231 | 32240 | LSC |
| 22 | p1 | (T)10 | 10 | 33152 | 33161 | LSC |
| 23 | c | (A)17(T)16 | 64 | 38638 | 38701 | LSC |
| 24 | p1 | (A)10 | 10 | 39338 | 39347 | LSC |
| 25 | p1 | (A)10 | 10 | 39703 | 39712 | LSC |
| 26 | p1 | (A)11 | 11 | 47678 | 47688 | LSC |
| 27 | p1 | (A)13 | 13 | 50409 | 50421 | LSC |
| 28 | c | (T)11(A)17 | 60 | 52370 | 52429 | LSC |
| 29 | p1 | (T)10 | 10 | 53843 | 53852 | LSC |
| 30 | p1 | (T)10 | 10 | 57877 | 57886 | LSC |
| 31 | p1 | (T)19 | 19 | 60592 | 60610 | LSC |
| 32 | c | (T)15(T)14 | 56 | 66964 | 67019 | LSC |
| 33 | p1 | (T)16 | 16 | 68734 | 68749 | LSC |
| 34 | p1 | (A)12 | 12 | 69062 | 69073 | LSC |
| 35 | p1 | (A)16 | 16 | 71001 | 71016 | LSC |
| 36 | p2 | (AT)6 | 12 | 71619 | 71630 | LSC |
| 37 | p1 | (T)12 | 12 | 72162 | 72173 | LSC |
| 38 | p1 | (T)12 | 12 | 72882 | 72893 | LSC |
| 39 | p1 | (T)14 | 14 | 74353 | 74366 | LSC |
| 40 | c | (A)12(T)16 | 67 | 75035 | 75101 | LSC |
| 41 | p1 | (A)11 | 11 | 80319 | 80329 | LSC |
| 42 | p1 | (A)11 | 11 | 81820 | 81830 | LSC |
| 43 | p1 | (T)17 | 17 | 83408 | 83424 | LSC |
| 44 | p1 | (T)10 | 10 | 84577 | 84586 | LSC |
| 45 | c | (T)10(A)13 | 53 | 85064 | 85116 | LSC |
| 46 | p1 | (T)19 | 19 | 86393 | 86411 | LSC |
| 47 | p1 | (T)10 | 10 | 87450 | 87459 | LSC |
| 48 | p1 | (T)10 | 10 | 104194 | 104203 | IRb |
| 49 | p1 | (A)10 | 10 | 112183 | 112192 | IRb |
| 50 | c | (A)23(A)10 | 100 | 116781 | 116880 | SSC |
| 51 | c | (T)15(T)11 | 100 | 117622 | 117721 | SSC |
| 52 | p2 | (AT)6 | 12 | 118705 | 118716 | SSC |
| 53 | p1 | (T)25 | 25 | 125961 | 125985 | SSC |
| 54 | p1 | (A)10 | 10 | 126531 | 126540 | SSC |
| 55 | p1 | (T)10 | 10 | 131880 | 131889 | SSC |
| 56 | p1 | (A)16 | 16 | 132511 | 132526 | SSC |
| 57 | p1 | (T)10 | 10 | 136061 | 136070 | IRa |
| 58 | p1 | (A)10 | 10 | 144050 | 144059 | IRa |

| *M. sieversii* | | | | | | |
| --- | --- | --- | --- | --- | --- | --- |
| SSR nr. | SSR type | SSR | size | start | end | region |
| 1 | c | (T)10act(A)15 | 28 | 216 | 243 | LSC |
| 2 | p1 | (T)11 | 11 | 1654 | 1664 | LSC |
| 3 | p1 | (T)11 | 11 | 2807 | 2817 | LSC |
| 4 | c | (C)11(A)11 | 22 | 5676 | 5697 | LSC |
| 5 | p1 | (A)10 | 10 | 6857 | 6866 | LSC |
| 6 | p1 | (A)14 | 14 | 7013 | 7026 | LSC |
| 7 | p1 | (A)12 | 12 | 7887 | 7898 | LSC |
| 8 | p1 | (T)10 | 10 | 8594 | 8603 | LSC |
| 9 | p1 | (T)12 | 12 | 9318 | 9329 | LSC |
| 10 | p1 | (T)10 | 10 | 12397 | 12406 | LSC |
| 11 | p1 | (T)15 | 15 | 13121 | 13135 | LSC |
| 12 | p1 | (T)10 | 10 | 13476 | 13485 | LSC |
| 13 | p1 | (A)11 | 11 | 14447 | 14457 | LSC |
| 14 | p1 | (C)10 | 10 | 14981 | 14990 | LSC |
| 15 | p1 | (A)12 | 12 | 15195 | 15206 | LSC |
| 16 | p1 | (T)13 | 13 | 15450 | 15462 | LSC |
| 17 | p1 | (T)14 | 14 | 17284 | 17297 | LSC |
| 18 | p1 | (T)11 | 11 | 19531 | 19541 | LSC |
| 19 | p1 | (T)10 | 10 | 27233 | 27242 | LSC |
| 20 | p1 | (T)12 | 12 | 27878 | 27889 | LSC |
| 21 | p1 | (A)13 | 13 | 28311 | 28323 | LSC |
| 22 | p1 | (A)11 | 11 | 28752 | 28762 | LSC |
| 23 | p1 | (A)12 | 12 | 32167 | 32178 | LSC |
| 24 | p1 | (T)11 | 11 | 33089 | 33099 | LSC |
| 25 | c | (A)17(T)13 | 61 | 38819 | 38879 | LSC |
| 26 | c | (ATA)5(A)10(T)11 | 68 | 39479 | 39546 | LSC |
| 27 | p1 | (A)10 | 10 | 39848 | 39857 | LSC |
| 28 | p1 | (A)11 | 11 | 45474 | 45484 | LSC |
| 29 | p1 | (T)10 | 10 | 45996 | 46005 | LSC |
| 30 | p1 | (A)11 | 11 | 47826 | 47836 | LSC |
| 31 | p1 | (A)13 | 13 | 50528 | 50540 | LSC |
| 32 | c | (T)11(A)18 | 61 | 52489 | 52549 | LSC |
| 33 | p1 | (T)10 | 10 | 58047 | 58056 | LSC |
| 34 | p1 | (T)15 | 15 | 60756 | 60770 | LSC |
| 35 | c | (T)18(T)14 | 59 | 67141 | 67199 | LSC |
| 36 | p1 | (T)17 | 17 | 68914 | 68930 | LSC |
| 37 | p1 | (A)12 | 12 | 69243 | 69254 | LSC |
| 38 | p2 | (AT)6 | 12 | 70350 | 70361 | LSC |
| 39 | p1 | (A)17 | 17 | 71203 | 71219 | LSC |
| 40 | p2 | (AT)6 | 12 | 71822 | 71833 | LSC |
| 41 | p1 | (T)10 | 10 | 72346 | 72355 | LSC |
| 42 | p1 | (T)16 | 16 | 73064 | 73079 | LSC |
| 43 | p1 | (T)11 | 11 | 74493 | 74503 | LSC |
| 44 | c | (A)12(T)13 | 68 | 75172 | 75239 | LSC |
| 45 | p1 | (A)10 | 10 | 80457 | 80466 | LSC |
| 46 | p1 | (A)11 | 11 | 81957 | 81967 | LSC |
| 47 | p1 | (T)16 | 16 | 83549 | 83564 | LSC |
| 48 | p1 | (T)10 | 10 | 84728 | 84737 | LSC |
| 49 | c | (T)10(A)12 | 57 | 85215 | 85271 | LSC |
| 50 | p1 | (T)15 | 15 | 86548 | 86562 | LSC |
| 51 | p1 | (T)10 | 10 | 86803 | 86812 | LSC |
| 52 | p1 | (T)10 | 10 | 87602 | 87611 | LSC |
| 53 | p1 | (T)10 | 10 | 104349 | 104358 | IRb |
| 54 | p1 | (A)10 | 10 | 112338 | 112347 | IRb |
| 55 | c | (A)20(A)10 | 97 | 116936 | 117032 | SSC |
| 56 | c | (T)14(C)13(T)10 | 102 | 117774 | 117875 | SSC |
| 57 | p1 | (T)10 | 10 | 118612 | 118621 | SSC |
| 58 | p2 | (AT)6 | 12 | 118875 | 118886 | SSC |
| 59 | p1 | (T)10 | 10 | 124207 | 124216 | SSC |
| 60 | c | (A)10(T)19 | 124 | 126015 | 126138 | SSC |
| 61 | p1 | (A)11 | 11 | 126684 | 126694 | SSC |
| 62 | p1 | (T)10 | 10 | 132034 | 132043 | SSC |
| 63 | p1 | (A)16 | 16 | 132665 | 132680 | SSC |
| 64 | p1 | (T)10 | 10 | 136215 | 136224 | IRa |
| 65 | p1 | (A)10 | 10 | 144204 | 144213 | IRa |
