# Supplementary material for: Complete chloroplast genome studies of different apple varieties indicated the origin of modern cultivated apples from Malus sieversii and Malus sylvestris
Source: PeerJ. 2022 Mar 18;10:e13107. doi: 10.7717/peerj.13107 (PMC8935992; doi:10.7717/peerj.13107)
Supplement: Supplemental Information 5 — WW: Wunan Town, Wuwei City; LX: Xingcheng, Liaoning; ZZ: Zhengzhou Fruit Tree Institute [file peerj-10-13107-s005.docx]

| Name | Minimum | Maximum | Length | Coverage | Polymorphism Type | Variant Frequency |
| --- | --- | --- | --- | --- | --- | --- |
| C | 162630 | 162630 | 1 | 26 | SNP | 69.20% |
| G | 162630 | 162630 | 1 | 26 | SNP | 30.80% |
| C | 140333 | 140333 | 1 | 26 | SNP | 30.80% |
| T | 140333 | 140333 | 1 | 26 | SNP | 69.20% |
| G | 139589 | 139589 | 1 | 26 | SNP | 30.80% |
| T | 139589 | 139589 | 1 | 26 | SNP | 69.20% |
| A | 139588 | 139588 | 1 | 26 | SNP | 30.80% |
| T | 139588 | 139588 | 1 | 26 | SNP | 69.20% |
| A | 139587 | 139587 | 1 | 26 | SNP | 30.80% |
| T | 139587 | 139587 | 1 | 26 | SNP | 69.20% |
| A | 139586 | 139586 | 1 | 26 | SNP | 30.80% |
| C | 139586 | 139586 | 1 | 26 | SNP | 69.20% |
| G | 139481 | 139481 | 1 | 26 | SNP | 69.20% |
| T | 139481 | 139481 | 1 | 26 | SNP | 30.80% |
| C | 131152 | 131152 | 1 | 26 | SNP | 34.60% |
| T | 131152 | 131152 | 1 | 26 | SNP | 65.40% |
| C | 130615 | 130615 | 1 | 26 | SNP | 65.40% |
| T | 130615 | 130615 | 1 | 26 | SNP | 34.60% |
| G | 130600 | 130600 | 1 | 26 | SNP | 65.40% |
| T | 130600 | 130600 | 1 | 26 | SNP | 34.60% |
| A | 118304 | 118304 | 1 | 26 | SNP | 34.60% |
| G | 118304 | 118304 | 1 | 26 | SNP | 65.40% |
| C | 117876 | 117876 | 1 | 26 | SNP | 30.80% |
| T | 117876 | 117876 | 1 | 26 | SNP | 69.20% |
| C | 116668 | 116668 | 1 | 26 | SNP | 30.80% |
| T | 116668 | 116668 | 1 | 26 | SNP | 69.20% |
| A | 116643 | 116643 | 1 | 26 | SNP | 34.60% |
| T | 116643 | 116643 | 1 | 26 | SNP | 65.40% |
| A | 115832 | 115832 | 1 | 26 | SNP | 34.60% |
| C | 115832 | 115832 | 1 | 26 | SNP | 65.40% |
| A | 114060 | 114060 | 1 | 26 | SNP | 30.80% |
| C | 114060 | 114060 | 1 | 26 | SNP | 69.20% |
| G | 113955 | 113955 | 1 | 26 | SNP | 69.20% |
| T | 113955 | 113955 | 1 | 26 | SNP | 30.80% |
| A | 113954 | 113954 | 1 | 26 | SNP | 69.20% |
| T | 113954 | 113954 | 1 | 26 | SNP | 30.80% |
| A | 113953 | 113953 | 1 | 26 | SNP | 69.20% |
| T | 113953 | 113953 | 1 | 26 | SNP | 30.80% |
| A | 113952 | 113952 | 1 | 26 | SNP | 69.20% |
| C | 113952 | 113952 | 1 | 26 | SNP | 30.80% |
| A | 113208 | 113208 | 1 | 26 | SNP | 69.20% |
| G | 113208 | 113208 | 1 | 26 | SNP | 30.80% |
| C | 90911 | 90911 | 1 | 26 | SNP | 30.80% |
| G | 90911 | 90911 | 1 | 26 | SNP | 69.20% |
| A | 89070 | 89070 | 1 | 26 | SNP | 30.80% |
| C | 89070 | 89070 | 1 | 26 | SNP | 69.20% |
| G | 87806 | 87806 | 1 | 26 | SNP | 30.80% |
| T | 87806 | 87806 | 1 | 26 | SNP | 69.20% |
| A | 87200 | 87200 | 1 | 26 | SNP | 69.20% |
| C | 87200 | 87200 | 1 | 26 | SNP | 30.80% |
| A | 86947 | 86947 | 1 | 26 | SNP | 30.80% |
| G | 86947 | 86947 | 1 | 26 | SNP | 69.20% |
| G | 85479 | 85479 | 1 | 26 | SNP | 30.80% |
| T | 85479 | 85479 | 1 | 26 | SNP | 69.20% |
| G | 85468 | 85468 | 1 | 26 | SNP | 26.90% |
| T | 85468 | 85468 | 1 | 26 | SNP | 73.10% |
| A | 85467 | 85467 | 1 | 26 | SNP | 30.80% |
| C | 85467 | 85467 | 1 | 26 | SNP | 69.20% |
| A | 84350 | 84350 | 1 | 26 | SNP | 30.80% |
| C | 84350 | 84350 | 1 | 26 | SNP | 69.20% |
| A | 84332 | 84332 | 1 | 26 | SNP | 30.80% |
| T | 84332 | 84332 | 1 | 26 | SNP | 69.20% |
| A | 84331 | 84331 | 1 | 26 | SNP | 69.20% |
| T | 84331 | 84331 | 1 | 26 | SNP | 30.80% |
| G | 84287 | 84287 | 1 | 26 | SNP | 30.80% |
| T | 84287 | 84287 | 1 | 26 | SNP | 65.40% |
| A | 83301 | 83301 | 1 | 26 | SNP | 69.20% |
| C | 83301 | 83301 | 1 | 26 | SNP | 30.80% |
| G | 82621 | 82621 | 1 | 26 | SNP | 30.80% |
| T | 82621 | 82621 | 1 | 26 | SNP | 69.20% |
| G | 81247 | 81247 | 1 | 26 | SNP | 30.80% |
| T | 81247 | 81247 | 1 | 26 | SNP | 69.20% |
| A | 79026 | 79026 | 1 | 26 | SNP | 30.80% |
| G | 79026 | 79026 | 1 | 26 | SNP | 69.20% |
| G | 78199 | 78199 | 1 | 26 | SNP | 30.80% |
| T | 78199 | 78199 | 1 | 26 | SNP | 69.20% |
| C | 75102 | 75102 | 1 | 26 | SNP | 69.20% |
| T | 75102 | 75102 | 1 | 26 | SNP | 30.80% |
| A | 73708 | 73708 | 1 | 26 | SNP | 30.80% |
| T | 73708 | 73708 | 1 | 26 | SNP | 69.20% |
| A | 72057 | 72057 | 1 | 26 | SNP | 30.80% |
| C | 72057 | 72057 | 1 | 26 | SNP | 69.20% |
| A | 71485 | 71485 | 1 | 26 | SNP | 69.20% |
| T | 71485 | 71485 | 1 | 26 | SNP | 30.80% |
| C | 71002 | 71002 | 1 | 26 | SNP | 69.20% |
| T | 71002 | 71002 | 1 | 26 | SNP | 30.80% |
| A | 70836 | 70836 | 1 | 26 | SNP | 26.90% |
| G | 70836 | 70836 | 1 | 26 | SNP | 69.20% |
| A | 70149 | 70149 | 1 | 26 | SNP | 30.80% |
| G | 70149 | 70149 | 1 | 26 | SNP | 69.20% |
| G | 70051 | 70051 | 1 | 26 | SNP | 30.80% |
| T | 70051 | 70051 | 1 | 26 | SNP | 69.20% |
| A | 69658 | 69658 | 1 | 26 | SNP | 69.20% |
| G | 69658 | 69658 | 1 | 26 | SNP | 30.80% |
| A | 69504 | 69504 | 1 | 26 | SNP | 30.80% |
| G | 69504 | 69504 | 1 | 26 | SNP | 69.20% |
| A | 67999 | 67999 | 1 | 26 | SNP | 69.20% |
| G | 67999 | 67999 | 1 | 26 | SNP | 30.80% |
| C | 67378 | 67378 | 1 | 26 | SNP | 30.80% |
| T | 67378 | 67378 | 1 | 26 | SNP | 69.20% |
| A | 64292 | 64292 | 1 | 26 | SNP | 69.20% |
| G | 64292 | 64292 | 1 | 26 | SNP | 30.80% |
| A | 64131 | 64131 | 1 | 26 | SNP | 30.80% |
| G | 64131 | 64131 | 1 | 26 | SNP | 69.20% |
| A | 63767 | 63767 | 1 | 26 | SNP | 69.20% |
| C | 63767 | 63767 | 1 | 26 | SNP | 30.80% |
| C | 63437 | 63437 | 1 | 26 | SNP | 69.20% |
| T | 63437 | 63437 | 1 | 26 | SNP | 30.80% |
| A | 63422 | 63422 | 1 | 26 | SNP | 69.20% |
| G | 63422 | 63422 | 1 | 26 | SNP | 30.80% |
| A | 63073 | 63073 | 1 | 26 | SNP | 30.80% |
| T | 63073 | 63073 | 1 | 26 | SNP | 69.20% |
| A | 63071 | 63071 | 1 | 26 | SNP | 69.20% |
| T | 63071 | 63071 | 1 | 26 | SNP | 30.80% |
| A | 63069 | 63069 | 1 | 26 | SNP | 30.80% |
| G | 63069 | 63069 | 1 | 26 | SNP | 69.20% |
| G | 63067 | 63067 | 1 | 26 | SNP | 69.20% |
| T | 63067 | 63067 | 1 | 26 | SNP | 30.80% |
| A | 63066 | 63066 | 1 | 26 | SNP | 69.20% |
| T | 63066 | 63066 | 1 | 26 | SNP | 30.80% |
| A | 63063 | 63063 | 1 | 26 | SNP | 69.20% |
| G | 63063 | 63063 | 1 | 26 | SNP | 30.80% |
| A | 63058 | 63058 | 1 | 26 | SNP | 69.20% |
| G | 63058 | 63058 | 1 | 26 | SNP | 30.80% |
| A | 63057 | 63057 | 1 | 26 | SNP | 30.80% |
| T | 63057 | 63057 | 1 | 26 | SNP | 69.20% |
| A | 61262 | 61262 | 1 | 26 | SNP | 73.10% |
| C | 61262 | 61262 | 1 | 26 | SNP | 26.90% |
| C | 58772 | 58772 | 1 | 26 | SNP | 69.20% |
| T | 58772 | 58772 | 1 | 26 | SNP | 30.80% |
| C | 56785 | 56785 | 1 | 26 | SNP | 30.80% |
| G | 56785 | 56785 | 1 | 26 | SNP | 69.20% |
| A | 55395 | 55395 | 1 | 26 | SNP | 30.80% |
| T | 55395 | 55395 | 1 | 26 | SNP | 69.20% |
| A | 55394 | 55394 | 1 | 26 | SNP | 69.20% |
| T | 55394 | 55394 | 1 | 26 | SNP | 30.80% |
| C | 55186 | 55186 | 1 | 26 | SNP | 26.90% |
| T | 55186 | 55186 | 1 | 26 | SNP | 73.10% |
| C | 54104 | 54104 | 1 | 26 | SNP | 69.20% |
| T | 54104 | 54104 | 1 | 26 | SNP | 30.80% |
| G | 54041 | 54041 | 1 | 26 | SNP | 30.80% |
| T | 54041 | 54041 | 1 | 26 | SNP | 69.20% |
| G | 53175 | 53175 | 1 | 26 | SNP | 69.20% |
| T | 53175 | 53175 | 1 | 26 | SNP | 30.80% |
| C | 51839 | 51839 | 1 | 26 | SNP | 30.80% |
| T | 51839 | 51839 | 1 | 26 | SNP | 69.20% |
| C | 51125 | 51125 | 1 | 26 | SNP | 30.80% |
| T | 51125 | 51125 | 1 | 26 | SNP | 69.20% |
| A | 51107 | 51107 | 1 | 26 | SNP | 69.20% |
| T | 51107 | 51107 | 1 | 26 | SNP | 30.80% |
| C | 51011 | 51011 | 1 | 26 | SNP | 73.10% |
| T | 51011 | 51011 | 1 | 26 | SNP | 26.90% |
| A | 50895 | 50895 | 1 | 26 | SNP | 69.20% |
| T | 50895 | 50895 | 1 | 26 | SNP | 30.80% |
| A | 50727 | 50727 | 1 | 26 | SNP | 69.20% |
| T | 50727 | 50727 | 1 | 26 | SNP | 30.80% |
| C | 48996 | 48996 | 1 | 26 | SNP | 30.80% |
| T | 48996 | 48996 | 1 | 26 | SNP | 69.20% |
| A | 45949 | 45949 | 1 | 26 | SNP | 30.80% |
| G | 45949 | 45949 | 1 | 26 | SNP | 69.20% |
| A | 40276 | 40276 | 1 | 26 | SNP | 30.80% |
| G | 40276 | 40276 | 1 | 26 | SNP | 69.20% |
| A | 40045 | 40045 | 1 | 26 | SNP | 30.80% |
| T | 40045 | 40045 | 1 | 26 | SNP | 69.20% |
| A | 40022 | 40022 | 1 | 26 | SNP | 69.20% |
| T | 40022 | 40022 | 1 | 26 | SNP | 30.80% |
| G | 39261 | 39261 | 1 | 26 | SNP | 69.20% |
| T | 39261 | 39261 | 1 | 26 | SNP | 30.80% |
| A | 33491 | 33491 | 1 | 26 | SNP | 69.20% |
| T | 33491 | 33491 | 1 | 26 | SNP | 30.80% |
| A | 33455 | 33455 | 1 | 26 | SNP | 73.10% |
| C | 33455 | 33455 | 1 | 26 | SNP | 26.90% |
| A | 33350 | 33350 | 1 | 26 | SNP | 69.20% |
| G | 33350 | 33350 | 1 | 26 | SNP | 30.80% |
| C | 32987 | 32987 | 1 | 26 | SNP | 69.20% |
| T | 32987 | 32987 | 1 | 26 | SNP | 30.80% |
| A | 32708 | 32708 | 1 | 26 | SNP | 73.10% |
| C | 32708 | 32708 | 1 | 26 | SNP | 26.90% |
| A | 32513 | 32513 | 1 | 26 | SNP | 65.40% |
| C | 32513 | 32513 | 1 | 26 | SNP | 30.80% |
| G | 32512 | 32512 | 1 | 26 | SNP | 65.40% |
| T | 32512 | 32512 | 1 | 26 | SNP | 30.80% |
| A | 31569 | 31569 | 1 | 26 | SNP | 73.10% |
| C | 31569 | 31569 | 1 | 26 | SNP | 26.90% |
| A | 31221 | 31221 | 1 | 26 | SNP | 26.90% |
| C | 31221 | 31221 | 1 | 26 | SNP | 73.10% |
| G | 30969 | 30969 | 1 | 26 | SNP | 69.20% |
| T | 30969 | 30969 | 1 | 26 | SNP | 30.80% |
| C | 30868 | 30868 | 1 | 26 | SNP | 69.20% |
| T | 30868 | 30868 | 1 | 26 | SNP | 30.80% |
| A | 30235 | 30235 | 1 | 26 | SNP | 30.80% |
| C | 30235 | 30235 | 1 | 26 | SNP | 69.20% |
| C | 24493 | 24493 | 1 | 26 | SNP | 69.20% |
| T | 24493 | 24493 | 1 | 26 | SNP | 30.80% |
| G | 24017 | 24017 | 1 | 26 | SNP | 73.10% |
| T | 24017 | 24017 | 1 | 26 | SNP | 26.90% |
| A | 22877 | 22877 | 1 | 26 | SNP | 69.20% |
| C | 22877 | 22877 | 1 | 26 | SNP | 30.80% |
| C | 19637 | 19637 | 1 | 26 | SNP | 30.80% |
| T | 19637 | 19637 | 1 | 26 | SNP | 69.20% |
| G | 18071 | 18071 | 1 | 26 | SNP | 30.80% |
| T | 18071 | 18071 | 1 | 26 | SNP | 69.20% |
| G | 17860 | 17860 | 1 | 26 | SNP | 30.80% |
| T | 17860 | 17860 | 1 | 26 | SNP | 69.20% |
| C | 17619 | 17619 | 1 | 26 | SNP | 30.80% |
| T | 17619 | 17619 | 1 | 26 | SNP | 69.20% |
| C | 17618 | 17618 | 1 | 26 | SNP | 69.20% |
| T | 17618 | 17618 | 1 | 26 | SNP | 30.80% |
| A | 16806 | 16806 | 1 | 26 | SNP | 30.80% |
| G | 16806 | 16806 | 1 | 26 | SNP | 69.20% |
| A | 14345 | 14345 | 1 | 26 | SNP | 30.80% |
| G | 14345 | 14345 | 1 | 26 | SNP | 69.20% |
| C | 13828 | 13828 | 1 | 26 | SNP | 30.80% |
| T | 13828 | 13828 | 1 | 26 | SNP | 69.20% |
| G | 12411 | 12411 | 1 | 26 | SNP | 30.80% |
| T | 12411 | 12411 | 1 | 26 | SNP | 69.20% |
| A | 12382 | 12382 | 1 | 26 | SNP | 30.80% |
| G | 12382 | 12382 | 1 | 26 | SNP | 69.20% |
| C | 11848 | 11848 | 1 | 26 | SNP | 30.80% |
| T | 11848 | 11848 | 1 | 26 | SNP | 69.20% |
| A | 10563 | 10563 | 1 | 26 | SNP | 69.20% |
| T | 10563 | 10563 | 1 | 26 | SNP | 30.80% |
| A | 10535 | 10535 | 1 | 26 | SNP | 65.40% |
| T | 10535 | 10535 | 1 | 26 | SNP | 30.80% |
| A | 10533 | 10533 | 1 | 26 | SNP | 30.80% |
| T | 10533 | 10533 | 1 | 26 | SNP | 65.40% |
| A | 10531 | 10531 | 1 | 26 | SNP | 30.80% |
| T | 10531 | 10531 | 1 | 26 | SNP | 65.40% |
| A | 10530 | 10530 | 1 | 26 | SNP | 65.40% |
| T | 10530 | 10530 | 1 | 26 | SNP | 30.80% |
| A | 10529 | 10529 | 1 | 26 | SNP | 30.80% |
| T | 10529 | 10529 | 1 | 26 | SNP | 65.40% |
| A | 10126 | 10126 | 1 | 26 | SNP | 69.20% |
| C | 10126 | 10126 | 1 | 26 | SNP | 30.80% |
| A | 9990 | 9990 | 1 | 26 | SNP | 69.20% |
| T | 9990 | 9990 | 1 | 26 | SNP | 30.80% |
| A | 9986 | 9986 | 1 | 26 | SNP | 69.20% |
| T | 9986 | 9986 | 1 | 26 | SNP | 30.80% |
| A | 9985 | 9985 | 1 | 26 | SNP | 69.20% |
| T | 9985 | 9985 | 1 | 26 | SNP | 30.80% |
| G | 9964 | 9964 | 1 | 26 | SNP | 26.90% |
| T | 9964 | 9964 | 1 | 26 | SNP | 73.10% |
| A | 8842 | 8842 | 1 | 26 | SNP | 69.20% |
| C | 8842 | 8842 | 1 | 26 | SNP | 30.80% |
| A | 8752 | 8752 | 1 | 26 | SNP | 69.20% |
| C | 8752 | 8752 | 1 | 26 | SNP | 30.80% |
| G | 8751 | 8751 | 1 | 26 | SNP | 69.20% |
| T | 8751 | 8751 | 1 | 26 | SNP | 30.80% |
| C | 7225 | 7225 | 1 | 26 | SNP | 30.80% |
| G | 7225 | 7225 | 1 | 26 | SNP | 69.20% |
| G | 6632 | 6632 | 1 | 26 | SNP | 26.90% |
| T | 6632 | 6632 | 1 | 26 | SNP | 73.10% |
| A | 6125 | 6125 | 1 | 26 | SNP | 69.20% |
| C | 6125 | 6125 | 1 | 26 | SNP | 30.80% |
| A | 5737 | 5737 | 1 | 26 | SNP | 73.10% |
| C | 5737 | 5737 | 1 | 26 | SNP | 26.90% |
| A | 5154 | 5154 | 1 | 26 | SNP | 30.80% |
| G | 5154 | 5154 | 1 | 26 | SNP | 69.20% |
| C | 4498 | 4498 | 1 | 26 | SNP | 69.20% |
| T | 4498 | 4498 | 1 | 26 | SNP | 30.80% |
| A | 4355 | 4355 | 1 | 26 | SNP | 69.20% |
| C | 4355 | 4355 | 1 | 26 | SNP | 30.80% |
| C | 4038 | 4038 | 1 | 26 | SNP | 30.80% |
| T | 4038 | 4038 | 1 | 26 | SNP | 69.20% |
| A | 3278 | 3278 | 1 | 26 | SNP | 69.20% |
| C | 3278 | 3278 | 1 | 26 | SNP | 30.80% |
| A | 2589 | 2589 | 1 | 26 | SNP | 69.20% |
| G | 2589 | 2589 | 1 | 26 | SNP | 30.80% |
| A | 282 | 282 | 1 | 26 | SNP | 30.80% |
| G | 282 | 282 | 1 | 26 | SNP | 69.20% |
