# Supplementary material for: Complete chloroplast genome studies of different apple varieties indicated the origin of modern cultivated apples from Malus sieversii and Malus sylvestris
Source: PeerJ. 2022 Mar 18;10:e13107. doi: 10.7717/peerj.13107 (PMC8935992; doi:10.7717/peerj.13107)
Supplement: Supplemental Information 6 [file peerj-10-13107-s006.docx]

| Name | Minimum | Maximum | Length | Coverage | Polymorphism Type | Variant Frequency |
| --- | --- | --- | --- | --- | --- | --- |
| T | 163699 | 163699 | 1 | 26 | Indel | 30.80% |
|  | 163699 | 163699 | 1 | 26 | Indel | 69.20% |
| A | 163698 | 163698 | 1 | 26 | Indel | 30.80% |
|  | 163698 | 163698 | 1 | 26 | Indel | 69.20% |
| A | 150361 | 150361 | 1 | 26 | Indel | 30.80% |
|  | 150361 | 150361 | 1 | 26 | Indel | 69.20% |
| C | 150360 | 150360 | 1 | 26 | Indel | 30.80% |
|  | 150360 | 150360 | 1 | 26 | Indel | 69.20% |
| A | 150359 | 150359 | 1 | 26 | Indel | 30.80% |
|  | 150359 | 150359 | 1 | 26 | Indel | 69.20% |
| T | 150358 | 150358 | 1 | 26 | Indel | 30.80% |
|  | 150358 | 150358 | 1 | 26 | Indel | 69.20% |
| A | 150357 | 150357 | 1 | 26 | Indel | 30.80% |
|  | 150357 | 150357 | 1 | 26 | Indel | 69.20% |
| T | 150356 | 150356 | 1 | 26 | Indel | 30.80% |
|  | 150356 | 150356 | 1 | 26 | Indel | 69.20% |
| T | 150355 | 150355 | 1 | 26 | Indel | 30.80% |
|  | 150355 | 150355 | 1 | 26 | Indel | 69.20% |
| T | 143892 | 143892 | 1 | 26 | Indel | 30.80% |
|  | 143892 | 143892 | 1 | 26 | Indel | 69.20% |
| T | 143891 | 143891 | 1 | 26 | Indel | 30.80% |
|  | 143891 | 143891 | 1 | 26 | Indel | 69.20% |
| T | 143890 | 143890 | 1 | 26 | Indel | 30.80% |
|  | 143890 | 143890 | 1 | 26 | Indel | 69.20% |
| C | 143889 | 143889 | 1 | 26 | Indel | 34.60% |
|  | 143889 | 143889 | 1 | 26 | Indel | 65.40% |
| A | 143888 | 143888 | 1 | 26 | Indel | 34.60% |
|  | 143888 | 143888 | 1 | 26 | Indel | 65.40% |
| A | 129994 | 129994 | 1 | 26 | Indel | 34.60% |
|  | 129994 | 129994 | 1 | 26 | Indel | 65.40% |
| T | 129303 | 129303 | 1 | 26 | Indel | 73.10% |
|  | 129303 | 129303 | 1 | 26 | Indel | 26.90% |
| A | 128990 | 128990 | 1 | 26 | Indel | 34.60% |
|  | 128990 | 128990 | 1 | 26 | Indel | 65.40% |
| A | 126016 | 126016 | 1 | 26 | Indel | 65.40% |
|  | 126016 | 126016 | 1 | 26 | Indel | 34.60% |
| A | 126015 | 126015 | 1 | 26 | Indel | 65.40% |
|  | 126015 | 126015 | 1 | 26 | Indel | 34.60% |
| C | 126014 | 126014 | 1 | 26 | Indel | 65.40% |
|  | 126014 | 126014 | 1 | 26 | Indel | 34.60% |
| T | 126013 | 126013 | 1 | 26 | Indel | 65.40% |
|  | 126013 | 126013 | 1 | 26 | Indel | 34.60% |
| T | 126012 | 126012 | 1 | 26 | Indel | 65.40% |
|  | 126012 | 126012 | 1 | 26 | Indel | 34.60% |
| A | 126011 | 126011 | 1 | 26 | Indel | 65.40% |
|  | 126011 | 126011 | 1 | 26 | Indel | 34.60% |
| G | 126010 | 126010 | 1 | 26 | Indel | 65.40% |
|  | 126010 | 126010 | 1 | 26 | Indel | 34.60% |
| A | 126009 | 126009 | 1 | 26 | Indel | 65.40% |
|  | 126009 | 126009 | 1 | 26 | Indel | 34.60% |
| T | 126008 | 126008 | 1 | 26 | Indel | 65.40% |
|  | 126008 | 126008 | 1 | 26 | Indel | 34.60% |
| A | 126007 | 126007 | 1 | 26 | Indel | 65.40% |
|  | 126007 | 126007 | 1 | 26 | Indel | 34.60% |
| A | 126006 | 126006 | 1 | 26 | Indel | 65.40% |
|  | 126006 | 126006 | 1 | 26 | Indel | 34.60% |
| G | 126005 | 126005 | 1 | 26 | Indel | 65.40% |
|  | 126005 | 126005 | 1 | 26 | Indel | 34.60% |
| A | 126004 | 126004 | 1 | 26 | Indel | 65.40% |
|  | 126004 | 126004 | 1 | 26 | Indel | 34.60% |
| T | 122919 | 122919 | 1 | 26 | Indel | 34.60% |
|  | 122919 | 122919 | 1 | 26 | Indel | 65.40% |
| T | 122917 | 122917 | 1 | 26 | Indel | 34.60% |
|  | 122917 | 122917 | 1 | 26 | Indel | 65.40% |
| T | 122915 | 122915 | 1 | 26 | Indel | 34.60% |
|  | 122915 | 122915 | 1 | 26 | Indel | 65.40% |
| T | 120568 | 120568 | 1 | 26 | Indel | 26.90% |
|  | 120568 | 120568 | 1 | 26 | Indel | 65.40% |
| T | 120273 | 120273 | 1 | 26 | Indel | 34.60% |
|  | 120273 | 120273 | 1 | 26 | Indel | 65.40% |
| T | 120269 | 120269 | 1 | 26 | Indel | 34.60% |
|  | 120269 | 120269 | 1 | 26 | Indel | 65.40% |
| A | 120268 | 120268 | 1 | 26 | Indel | 34.60% |
|  | 120268 | 120268 | 1 | 26 | Indel | 65.40% |
| T | 120261 | 120261 | 1 | 26 | Indel | 34.60% |
|  | 120261 | 120261 | 1 | 26 | Indel | 65.40% |
| A | 120259 | 120259 | 1 | 26 | Indel | 34.60% |
|  | 120259 | 120259 | 1 | 26 | Indel | 65.40% |
| G | 109647 | 109647 | 1 | 26 | Indel | 30.80% |
|  | 109647 | 109647 | 1 | 26 | Indel | 69.20% |
| A | 109646 | 109646 | 1 | 26 | Indel | 30.80% |
|  | 109646 | 109646 | 1 | 26 | Indel | 69.20% |
| A | 109645 | 109645 | 1 | 26 | Indel | 30.80% |
|  | 109645 | 109645 | 1 | 26 | Indel | 69.20% |
| A | 109644 | 109644 | 1 | 26 | Indel | 30.80% |
|  | 109644 | 109644 | 1 | 26 | Indel | 69.20% |
| T | 109643 | 109643 | 1 | 26 | Indel | 30.80% |
|  | 109643 | 109643 | 1 | 26 | Indel | 69.20% |
| T | 103182 | 103182 | 1 | 26 | Indel | 30.80% |
|  | 103182 | 103182 | 1 | 26 | Indel | 69.20% |
| G | 103181 | 103181 | 1 | 26 | Indel | 30.80% |
|  | 103181 | 103181 | 1 | 26 | Indel | 69.20% |
| T | 103180 | 103180 | 1 | 26 | Indel | 30.80% |
|  | 103180 | 103180 | 1 | 26 | Indel | 69.20% |
| A | 103179 | 103179 | 1 | 26 | Indel | 30.80% |
|  | 103179 | 103179 | 1 | 26 | Indel | 69.20% |
| A | 103178 | 103178 | 1 | 26 | Indel | 30.80% |
|  | 103178 | 103178 | 1 | 26 | Indel | 69.20% |
| T | 103177 | 103177 | 1 | 26 | Indel | 30.80% |
|  | 103177 | 103177 | 1 | 26 | Indel | 69.20% |
| A | 103176 | 103176 | 1 | 26 | Indel | 30.80% |
|  | 103176 | 103176 | 1 | 26 | Indel | 69.20% |
| A | 89834 | 89834 | 1 | 26 | Indel | 30.80% |
|  | 89834 | 89834 | 1 | 26 | Indel | 69.20% |
| T | 89833 | 89833 | 1 | 26 | Indel | 30.80% |
|  | 89833 | 89833 | 1 | 26 | Indel | 69.20% |
| T | 87548 | 87548 | 1 | 26 | Indel | 30.80% |
|  | 87548 | 87548 | 1 | 26 | Indel | 69.20% |
| T | 87291 | 87291 | 1 | 26 | Indel | 69.20% |
|  | 87291 | 87291 | 1 | 26 | Indel | 30.80% |
| T | 87290 | 87290 | 1 | 26 | Indel | 69.20% |
|  | 87290 | 87290 | 1 | 26 | Indel | 30.80% |
| T | 87289 | 87289 | 1 | 26 | Indel | 69.20% |
|  | 87289 | 87289 | 1 | 26 | Indel | 30.80% |
| A | 86000 | 86000 | 1 | 26 | Indel | 69.20% |
|  | 86000 | 86000 | 1 | 26 | Indel | 30.80% |
| A | 85984 | 85984 | 1 | 26 | Indel | 30.80% |
|  | 85984 | 85984 | 1 | 26 | Indel | 69.20% |
| A | 85983 | 85983 | 1 | 26 | Indel | 30.80% |
|  | 85983 | 85983 | 1 | 26 | Indel | 69.20% |
| C | 85982 | 85982 | 1 | 26 | Indel | 30.80% |
|  | 85982 | 85982 | 1 | 26 | Indel | 69.20% |
| T | 85981 | 85981 | 1 | 26 | Indel | 30.80% |
|  | 85981 | 85981 | 1 | 26 | Indel | 69.20% |
| T | 85980 | 85980 | 1 | 26 | Indel | 30.80% |
|  | 85980 | 85980 | 1 | 26 | Indel | 69.20% |
| A | 84327 | 84327 | 1 | 26 | Indel | 30.80% |
|  | 84327 | 84327 | 1 | 26 | Indel | 69.20% |
| G | 84326 | 84326 | 1 | 26 | Indel | 30.80% |
|  | 84326 | 84326 | 1 | 26 | Indel | 69.20% |
| T | 84325 | 84325 | 1 | 26 | Indel | 30.80% |
|  | 84325 | 84325 | 1 | 26 | Indel | 69.20% |
| T | 84324 | 84324 | 1 | 26 | Indel | 30.80% |
|  | 84324 | 84324 | 1 | 26 | Indel | 69.20% |
| T | 84323 | 84323 | 1 | 26 | Indel | 30.80% |
|  | 84323 | 84323 | 1 | 26 | Indel | 69.20% |
| A | 84322 | 84322 | 1 | 26 | Indel | 30.80% |
|  | 84322 | 84322 | 1 | 26 | Indel | 69.20% |
| T | 84321 | 84321 | 1 | 26 | Indel | 30.80% |
|  | 84321 | 84321 | 1 | 26 | Indel | 69.20% |
| T | 84320 | 84320 | 1 | 26 | Indel | 30.80% |
|  | 84320 | 84320 | 1 | 26 | Indel | 69.20% |
| T | 84319 | 84319 | 1 | 26 | Indel | 30.80% |
|  | 84319 | 84319 | 1 | 26 | Indel | 69.20% |
| A | 84318 | 84318 | 1 | 26 | Indel | 30.80% |
|  | 84318 | 84318 | 1 | 26 | Indel | 69.20% |
| T | 84317 | 84317 | 1 | 26 | Indel | 30.80% |
|  | 84317 | 84317 | 1 | 26 | Indel | 69.20% |
| T | 84299 | 84299 | 1 | 26 | Indel | 30.80% |
|  | 84299 | 84299 | 1 | 26 | Indel | 69.20% |
| T | 84298 | 84298 | 1 | 26 | Indel | 34.60% |
|  | 84298 | 84298 | 1 | 26 | Indel | 65.40% |
| T | 84297 | 84297 | 1 | 26 | Indel | 34.60% |
|  | 84297 | 84297 | 1 | 26 | Indel | 65.40% |
| T | 84296 | 84296 | 1 | 26 | Indel | 34.60% |
|  | 84296 | 84296 | 1 | 26 | Indel | 65.40% |
| A | 82695 | 82695 | 1 | 26 | Indel | 26.90% |
|  | 82695 | 82695 | 1 | 26 | Indel | 73.10% |
| A | 81194 | 81194 | 1 | 26 | Indel | 69.20% |
|  | 81194 | 81194 | 1 | 26 | Indel | 30.80% |
| A | 80307 | 80307 | 1 | 26 | Indel | 26.90% |
|  | 80307 | 80307 | 1 | 26 | Indel | 73.10% |
| A | 75962 | 75962 | 1 | 26 | Indel | 30.80% |
|  | 75962 | 75962 | 1 | 26 | Indel | 69.20% |
| T | 75228 | 75228 | 1 | 26 | Indel | 69.20% |
|  | 75228 | 75228 | 1 | 26 | Indel | 30.80% |
| T | 75227 | 75227 | 1 | 26 | Indel | 69.20% |
|  | 75227 | 75227 | 1 | 26 | Indel | 30.80% |
| T | 75226 | 75226 | 1 | 26 | Indel | 69.20% |
|  | 75226 | 75226 | 1 | 26 | Indel | 30.80% |
| T | 75077 | 75077 | 1 | 26 | Indel | 69.20% |
|  | 75077 | 75077 | 1 | 26 | Indel | 30.80% |
| A | 75076 | 75076 | 1 | 26 | Indel | 69.20% |
|  | 75076 | 75076 | 1 | 26 | Indel | 30.80% |
| A | 75075 | 75075 | 1 | 26 | Indel | 69.20% |
|  | 75075 | 75075 | 1 | 26 | Indel | 30.80% |
| A | 75074 | 75074 | 1 | 26 | Indel | 69.20% |
|  | 75074 | 75074 | 1 | 26 | Indel | 30.80% |
| C | 75073 | 75073 | 1 | 26 | Indel | 69.20% |
|  | 75073 | 75073 | 1 | 26 | Indel | 30.80% |
| T | 75072 | 75072 | 1 | 26 | Indel | 69.20% |
|  | 75072 | 75072 | 1 | 26 | Indel | 30.80% |
| A | 75071 | 75071 | 1 | 26 | Indel | 69.20% |
|  | 75071 | 75071 | 1 | 26 | Indel | 30.80% |
| T | 75070 | 75070 | 1 | 26 | Indel | 69.20% |
|  | 75070 | 75070 | 1 | 26 | Indel | 30.80% |
| A | 75069 | 75069 | 1 | 26 | Indel | 69.20% |
|  | 75069 | 75069 | 1 | 26 | Indel | 30.80% |
| A | 75068 | 75068 | 1 | 26 | Indel | 69.20% |
|  | 75068 | 75068 | 1 | 26 | Indel | 30.80% |
| A | 75067 | 75067 | 1 | 26 | Indel | 69.20% |
|  | 75067 | 75067 | 1 | 26 | Indel | 30.80% |
| A | 75066 | 75066 | 1 | 26 | Indel | 69.20% |
|  | 75066 | 75066 | 1 | 26 | Indel | 30.80% |
| T | 75065 | 75065 | 1 | 26 | Indel | 69.20% |
|  | 75065 | 75065 | 1 | 26 | Indel | 30.80% |
| A | 75064 | 75064 | 1 | 26 | Indel | 69.20% |
|  | 75064 | 75064 | 1 | 26 | Indel | 30.80% |
| T | 75063 | 75063 | 1 | 26 | Indel | 69.20% |
|  | 75063 | 75063 | 1 | 26 | Indel | 30.80% |
| A | 75062 | 75062 | 1 | 26 | Indel | 69.20% |
|  | 75062 | 75062 | 1 | 26 | Indel | 30.80% |
| A | 75061 | 75061 | 1 | 26 | Indel | 69.20% |
|  | 75061 | 75061 | 1 | 26 | Indel | 30.80% |
| A | 75060 | 75060 | 1 | 26 | Indel | 69.20% |
|  | 75060 | 75060 | 1 | 26 | Indel | 30.80% |
| T | 75059 | 75059 | 1 | 26 | Indel | 69.20% |
|  | 75059 | 75059 | 1 | 26 | Indel | 30.80% |
| T | 75058 | 75058 | 1 | 26 | Indel | 69.20% |
|  | 75058 | 75058 | 1 | 26 | Indel | 30.80% |
| A | 75057 | 75057 | 1 | 26 | Indel | 69.20% |
|  | 75057 | 75057 | 1 | 26 | Indel | 30.80% |
| A | 75056 | 75056 | 1 | 26 | Indel | 69.20% |
|  | 75056 | 75056 | 1 | 26 | Indel | 30.80% |
| A | 75055 | 75055 | 1 | 26 | Indel | 69.20% |
|  | 75055 | 75055 | 1 | 26 | Indel | 30.80% |
| T | 74584 | 74584 | 1 | 26 | Indel | 69.20% |
|  | 74584 | 74584 | 1 | 26 | Indel | 30.80% |
| A | 74583 | 74583 | 1 | 26 | Indel | 69.20% |
|  | 74583 | 74583 | 1 | 26 | Indel | 30.80% |
| G | 74582 | 74582 | 1 | 26 | Indel | 69.20% |
|  | 74582 | 74582 | 1 | 26 | Indel | 30.80% |
| A | 74581 | 74581 | 1 | 26 | Indel | 69.20% |
|  | 74581 | 74581 | 1 | 26 | Indel | 30.80% |
| A | 74580 | 74580 | 1 | 26 | Indel | 69.20% |
|  | 74580 | 74580 | 1 | 26 | Indel | 30.80% |
| A | 74579 | 74579 | 1 | 26 | Indel | 69.20% |
|  | 74579 | 74579 | 1 | 26 | Indel | 30.80% |
| A | 74578 | 74578 | 1 | 26 | Indel | 69.20% |
|  | 74578 | 74578 | 1 | 26 | Indel | 30.80% |
| T | 74577 | 74577 | 1 | 26 | Indel | 69.20% |
|  | 74577 | 74577 | 1 | 26 | Indel | 30.80% |
| T | 74576 | 74576 | 1 | 26 | Indel | 69.20% |
|  | 74576 | 74576 | 1 | 26 | Indel | 30.80% |
| A | 74575 | 74575 | 1 | 26 | Indel | 69.20% |
|  | 74575 | 74575 | 1 | 26 | Indel | 30.80% |
| T | 74574 | 74574 | 1 | 26 | Indel | 69.20% |
|  | 74574 | 74574 | 1 | 26 | Indel | 30.80% |
| A | 74573 | 74573 | 1 | 26 | Indel | 69.20% |
|  | 74573 | 74573 | 1 | 26 | Indel | 30.80% |
| A | 74572 | 74572 | 1 | 26 | Indel | 69.20% |
|  | 74572 | 74572 | 1 | 26 | Indel | 30.80% |
| G | 74571 | 74571 | 1 | 26 | Indel | 69.20% |
|  | 74571 | 74571 | 1 | 26 | Indel | 30.80% |
| A | 74570 | 74570 | 1 | 26 | Indel | 69.20% |
|  | 74570 | 74570 | 1 | 26 | Indel | 30.80% |
| T | 74569 | 74569 | 1 | 26 | Indel | 69.20% |
|  | 74569 | 74569 | 1 | 26 | Indel | 30.80% |
| A | 74568 | 74568 | 1 | 26 | Indel | 69.20% |
|  | 74568 | 74568 | 1 | 26 | Indel | 30.80% |
| A | 74567 | 74567 | 1 | 26 | Indel | 69.20% |
|  | 74567 | 74567 | 1 | 26 | Indel | 30.80% |
| G | 74566 | 74566 | 1 | 26 | Indel | 69.20% |
|  | 74566 | 74566 | 1 | 26 | Indel | 30.80% |
| T | 74565 | 74565 | 1 | 26 | Indel | 69.20% |
|  | 74565 | 74565 | 1 | 26 | Indel | 30.80% |
| A | 74564 | 74564 | 1 | 26 | Indel | 69.20% |
|  | 74564 | 74564 | 1 | 26 | Indel | 30.80% |
| A | 74563 | 74563 | 1 | 26 | Indel | 69.20% |
|  | 74563 | 74563 | 1 | 26 | Indel | 30.80% |
| T | 74562 | 74562 | 1 | 26 | Indel | 69.20% |
|  | 74562 | 74562 | 1 | 26 | Indel | 30.80% |
| T | 73754 | 73754 | 1 | 26 | Indel | 30.80% |
|  | 73754 | 73754 | 1 | 26 | Indel | 69.20% |
| T | 73753 | 73753 | 1 | 26 | Indel | 30.80% |
|  | 73753 | 73753 | 1 | 26 | Indel | 69.20% |
| T | 73752 | 73752 | 1 | 26 | Indel | 30.80% |
|  | 73752 | 73752 | 1 | 26 | Indel | 69.20% |
| T | 73751 | 73751 | 1 | 26 | Indel | 30.80% |
|  | 73751 | 73751 | 1 | 26 | Indel | 69.20% |
| T | 73032 | 73032 | 1 | 26 | Indel | 69.20% |
|  | 73032 | 73032 | 1 | 26 | Indel | 30.80% |
| T | 73031 | 73031 | 1 | 26 | Indel | 69.20% |
|  | 73031 | 73031 | 1 | 26 | Indel | 30.80% |
| A | 72538 | 72538 | 1 | 26 | Indel | 69.20% |
|  | 72538 | 72538 | 1 | 26 | Indel | 30.80% |
| A | 72537 | 72537 | 1 | 26 | Indel | 69.20% |
|  | 72537 | 72537 | 1 | 26 | Indel | 30.80% |
| T | 72536 | 72536 | 1 | 26 | Indel | 69.20% |
|  | 72536 | 72536 | 1 | 26 | Indel | 30.80% |
| A | 72535 | 72535 | 1 | 26 | Indel | 69.20% |
|  | 72535 | 72535 | 1 | 26 | Indel | 30.80% |
| T | 72534 | 72534 | 1 | 26 | Indel | 69.20% |
|  | 72534 | 72534 | 1 | 26 | Indel | 30.80% |
| A | 72533 | 72533 | 1 | 26 | Indel | 69.20% |
|  | 72533 | 72533 | 1 | 26 | Indel | 30.80% |
| A | 72532 | 72532 | 1 | 26 | Indel | 69.20% |
|  | 72532 | 72532 | 1 | 26 | Indel | 30.80% |
| A | 72531 | 72531 | 1 | 26 | Indel | 69.20% |
|  | 72531 | 72531 | 1 | 26 | Indel | 30.80% |
| T | 72530 | 72530 | 1 | 26 | Indel | 69.20% |
|  | 72530 | 72530 | 1 | 26 | Indel | 30.80% |
| A | 72529 | 72529 | 1 | 26 | Indel | 69.20% |
|  | 72529 | 72529 | 1 | 26 | Indel | 30.80% |
| A | 72528 | 72528 | 1 | 26 | Indel | 69.20% |
|  | 72528 | 72528 | 1 | 26 | Indel | 30.80% |
| A | 72527 | 72527 | 1 | 26 | Indel | 69.20% |
|  | 72527 | 72527 | 1 | 26 | Indel | 30.80% |
| G | 72526 | 72526 | 1 | 26 | Indel | 69.20% |
|  | 72526 | 72526 | 1 | 26 | Indel | 30.80% |
| A | 72525 | 72525 | 1 | 26 | Indel | 69.20% |
|  | 72525 | 72525 | 1 | 26 | Indel | 30.80% |
| T | 72524 | 72524 | 1 | 26 | Indel | 69.20% |
|  | 72524 | 72524 | 1 | 26 | Indel | 30.80% |
| A | 72523 | 72523 | 1 | 26 | Indel | 69.20% |
|  | 72523 | 72523 | 1 | 26 | Indel | 30.80% |
| A | 72522 | 72522 | 1 | 26 | Indel | 69.20% |
|  | 72522 | 72522 | 1 | 26 | Indel | 30.80% |
| A | 72521 | 72521 | 1 | 26 | Indel | 69.20% |
|  | 72521 | 72521 | 1 | 26 | Indel | 30.80% |
| T | 72520 | 72520 | 1 | 26 | Indel | 69.20% |
|  | 72520 | 72520 | 1 | 26 | Indel | 30.80% |
| A | 71869 | 71869 | 1 | 26 | Indel | 30.80% |
|  | 71869 | 71869 | 1 | 26 | Indel | 69.20% |
| T | 71821 | 71821 | 1 | 26 | Indel | 30.80% |
|  | 71821 | 71821 | 1 | 26 | Indel | 69.20% |
| T | 71017 | 71017 | 1 | 26 | Indel | 30.80% |
|  | 71017 | 71017 | 1 | 26 | Indel | 69.20% |
| A | 71016 | 71016 | 1 | 26 | Indel | 34.60% |
|  | 71016 | 71016 | 1 | 26 | Indel | 65.40% |
| T | 69574 | 69574 | 1 | 26 | Indel | 30.80% |
|  | 69574 | 69574 | 1 | 26 | Indel | 69.20% |
| T | 67857 | 67857 | 1 | 26 | Indel | 30.80% |
|  | 67857 | 67857 | 1 | 26 | Indel | 69.20% |
| T | 67802 | 67802 | 1 | 26 | Indel | 30.80% |
|  | 67802 | 67802 | 1 | 26 | Indel | 69.20% |
| T | 67801 | 67801 | 1 | 26 | Indel | 30.80% |
|  | 67801 | 67801 | 1 | 26 | Indel | 69.20% |
| T | 67800 | 67800 | 1 | 26 | Indel | 30.80% |
|  | 67800 | 67800 | 1 | 26 | Indel | 69.20% |
| T | 67799 | 67799 | 1 | 26 | Indel | 26.90% |
|  | 67799 | 67799 | 1 | 26 | Indel | 73.10% |
| A | 64332 | 64332 | 1 | 26 | Indel | 30.80% |
|  | 64332 | 64332 | 1 | 26 | Indel | 69.20% |
| T | 63645 | 63645 | 1 | 26 | Indel | 30.80% |
|  | 63645 | 63645 | 1 | 26 | Indel | 69.20% |
| A | 63644 | 63644 | 1 | 26 | Indel | 30.80% |
|  | 63644 | 63644 | 1 | 26 | Indel | 69.20% |
| A | 63643 | 63643 | 1 | 26 | Indel | 30.80% |
|  | 63643 | 63643 | 1 | 26 | Indel | 69.20% |
| T | 63642 | 63642 | 1 | 26 | Indel | 30.80% |
|  | 63642 | 63642 | 1 | 26 | Indel | 69.20% |
| T | 63641 | 63641 | 1 | 26 | Indel | 30.80% |
|  | 63641 | 63641 | 1 | 26 | Indel | 69.20% |
| A | 63567 | 63567 | 1 | 26 | Indel | 30.80% |
|  | 63567 | 63567 | 1 | 26 | Indel | 69.20% |
| A | 63566 | 63566 | 1 | 26 | Indel | 30.80% |
|  | 63566 | 63566 | 1 | 26 | Indel | 69.20% |
| A | 63565 | 63565 | 1 | 26 | Indel | 30.80% |
|  | 63565 | 63565 | 1 | 26 | Indel | 69.20% |
| G | 63564 | 63564 | 1 | 26 | Indel | 30.80% |
|  | 63564 | 63564 | 1 | 26 | Indel | 69.20% |
| A | 63563 | 63563 | 1 | 26 | Indel | 30.80% |
|  | 63563 | 63563 | 1 | 26 | Indel | 69.20% |
| T | 63562 | 63562 | 1 | 26 | Indel | 30.80% |
|  | 63562 | 63562 | 1 | 26 | Indel | 69.20% |
| T | 63561 | 63561 | 1 | 26 | Indel | 30.80% |
|  | 63561 | 63561 | 1 | 26 | Indel | 69.20% |
| A | 63560 | 63560 | 1 | 26 | Indel | 30.80% |
|  | 63560 | 63560 | 1 | 26 | Indel | 69.20% |
| A | 63559 | 63559 | 1 | 26 | Indel | 30.80% |
|  | 63559 | 63559 | 1 | 26 | Indel | 69.20% |
| G | 63558 | 63558 | 1 | 26 | Indel | 30.80% |
|  | 63558 | 63558 | 1 | 26 | Indel | 69.20% |
| T | 63557 | 63557 | 1 | 26 | Indel | 30.80% |
|  | 63557 | 63557 | 1 | 26 | Indel | 69.20% |
| A | 63556 | 63556 | 1 | 26 | Indel | 30.80% |
|  | 63556 | 63556 | 1 | 26 | Indel | 69.20% |
| G | 63555 | 63555 | 1 | 26 | Indel | 30.80% |
|  | 63555 | 63555 | 1 | 26 | Indel | 69.20% |
| A | 63554 | 63554 | 1 | 26 | Indel | 30.80% |
|  | 63554 | 63554 | 1 | 26 | Indel | 69.20% |
| A | 63553 | 63553 | 1 | 26 | Indel | 30.80% |
|  | 63553 | 63553 | 1 | 26 | Indel | 69.20% |
| A | 63552 | 63552 | 1 | 26 | Indel | 30.80% |
|  | 63552 | 63552 | 1 | 26 | Indel | 69.20% |
| G | 63551 | 63551 | 1 | 26 | Indel | 30.80% |
|  | 63551 | 63551 | 1 | 26 | Indel | 69.20% |
| A | 63550 | 63550 | 1 | 26 | Indel | 30.80% |
|  | 63550 | 63550 | 1 | 26 | Indel | 69.20% |
| T | 63549 | 63549 | 1 | 26 | Indel | 30.80% |
|  | 63549 | 63549 | 1 | 26 | Indel | 69.20% |
| G | 63548 | 63548 | 1 | 26 | Indel | 30.80% |
|  | 63548 | 63548 | 1 | 26 | Indel | 69.20% |
| T | 63547 | 63547 | 1 | 26 | Indel | 30.80% |
|  | 63547 | 63547 | 1 | 26 | Indel | 69.20% |
| A | 63546 | 63546 | 1 | 26 | Indel | 30.80% |
|  | 63546 | 63546 | 1 | 26 | Indel | 69.20% |
| C | 63545 | 63545 | 1 | 26 | Indel | 30.80% |
|  | 63545 | 63545 | 1 | 26 | Indel | 69.20% |
| T | 63061 | 63061 | 1 | 26 | Indel | 69.20% |
|  | 63061 | 63061 | 1 | 26 | Indel | 30.80% |
| T | 63060 | 63060 | 1 | 26 | Indel | 69.20% |
|  | 63060 | 63060 | 1 | 26 | Indel | 30.80% |
| T | 63059 | 63059 | 1 | 26 | Indel | 69.20% |
|  | 63059 | 63059 | 1 | 26 | Indel | 30.80% |
| G | 61429 | 61429 | 1 | 26 | Indel | 69.20% |
|  | 61429 | 61429 | 1 | 26 | Indel | 30.80% |
| C | 61428 | 61428 | 1 | 26 | Indel | 69.20% |
|  | 61428 | 61428 | 1 | 26 | Indel | 30.80% |
| T | 61427 | 61427 | 1 | 26 | Indel | 69.20% |
|  | 61427 | 61427 | 1 | 26 | Indel | 30.80% |
| C | 61426 | 61426 | 1 | 26 | Indel | 69.20% |
|  | 61426 | 61426 | 1 | 26 | Indel | 30.80% |
| C | 61425 | 61425 | 1 | 26 | Indel | 69.20% |
|  | 61425 | 61425 | 1 | 26 | Indel | 30.80% |
| C | 61424 | 61424 | 1 | 26 | Indel | 69.20% |
|  | 61424 | 61424 | 1 | 26 | Indel | 30.80% |
| C | 61423 | 61423 | 1 | 26 | Indel | 69.20% |
|  | 61423 | 61423 | 1 | 26 | Indel | 30.80% |
| T | 61422 | 61422 | 1 | 26 | Indel | 69.20% |
|  | 61422 | 61422 | 1 | 26 | Indel | 30.80% |
| A | 61421 | 61421 | 1 | 26 | Indel | 69.20% |
|  | 61421 | 61421 | 1 | 26 | Indel | 30.80% |
| T | 61399 | 61399 | 1 | 26 | Indel | 69.20% |
|  | 61399 | 61399 | 1 | 26 | Indel | 30.80% |
| A | 60964 | 60964 | 1 | 26 | Indel | 69.20% |
|  | 60964 | 60964 | 1 | 26 | Indel | 30.80% |
| G | 60963 | 60963 | 1 | 26 | Indel | 69.20% |
|  | 60963 | 60963 | 1 | 26 | Indel | 30.80% |
| T | 60962 | 60962 | 1 | 26 | Indel | 69.20% |
|  | 60962 | 60962 | 1 | 26 | Indel | 30.80% |
| T | 60961 | 60961 | 1 | 26 | Indel | 69.20% |
|  | 60961 | 60961 | 1 | 26 | Indel | 30.80% |
| A | 60960 | 60960 | 1 | 26 | Indel | 69.20% |
|  | 60960 | 60960 | 1 | 26 | Indel | 30.80% |
| A | 60959 | 60959 | 1 | 26 | Indel | 69.20% |
|  | 60959 | 60959 | 1 | 26 | Indel | 30.80% |
| A | 56442 | 56442 | 1 | 26 | Indel | 69.20% |
|  | 56442 | 56442 | 1 | 26 | Indel | 30.80% |
| T | 56441 | 56441 | 1 | 26 | Indel | 69.20% |
|  | 56441 | 56441 | 1 | 26 | Indel | 30.80% |
| T | 56440 | 56440 | 1 | 26 | Indel | 69.20% |
|  | 56440 | 56440 | 1 | 26 | Indel | 30.80% |
| C | 56439 | 56439 | 1 | 26 | Indel | 69.20% |
|  | 56439 | 56439 | 1 | 26 | Indel | 30.80% |
| C | 55664 | 55664 | 1 | 26 | Indel | 73.10% |
|  | 55664 | 55664 | 1 | 26 | Indel | 26.90% |
| T | 55663 | 55663 | 1 | 26 | Indel | 73.10% |
|  | 55663 | 55663 | 1 | 26 | Indel | 26.90% |
| T | 55662 | 55662 | 1 | 26 | Indel | 73.10% |
|  | 55662 | 55662 | 1 | 26 | Indel | 26.90% |
| T | 55661 | 55661 | 1 | 26 | Indel | 73.10% |
|  | 55661 | 55661 | 1 | 26 | Indel | 26.90% |
| T | 55660 | 55660 | 1 | 26 | Indel | 73.10% |
|  | 55660 | 55660 | 1 | 26 | Indel | 26.90% |
| A | 55659 | 55659 | 1 | 26 | Indel | 73.10% |
|  | 55659 | 55659 | 1 | 26 | Indel | 26.90% |
| T | 55584 | 55584 | 1 | 26 | Indel | 69.20% |
|  | 55584 | 55584 | 1 | 26 | Indel | 30.80% |
| A | 55583 | 55583 | 1 | 26 | Indel | 69.20% |
|  | 55583 | 55583 | 1 | 26 | Indel | 30.80% |
| T | 55392 | 55392 | 1 | 26 | Indel | 30.80% |
|  | 55392 | 55392 | 1 | 26 | Indel | 69.20% |
| A | 55391 | 55391 | 1 | 26 | Indel | 30.80% |
|  | 55391 | 55391 | 1 | 26 | Indel | 69.20% |
| A | 55390 | 55390 | 1 | 26 | Indel | 30.80% |
|  | 55390 | 55390 | 1 | 26 | Indel | 69.20% |
| T | 55389 | 55389 | 1 | 26 | Indel | 30.80% |
|  | 55389 | 55389 | 1 | 26 | Indel | 69.20% |
| A | 55388 | 55388 | 1 | 26 | Indel | 30.80% |
|  | 55388 | 55388 | 1 | 26 | Indel | 69.20% |
| T | 55387 | 55387 | 1 | 26 | Indel | 30.80% |
|  | 55387 | 55387 | 1 | 26 | Indel | 69.20% |
| A | 55386 | 55386 | 1 | 26 | Indel | 30.80% |
|  | 55386 | 55386 | 1 | 26 | Indel | 69.20% |
| T | 55385 | 55385 | 1 | 26 | Indel | 30.80% |
|  | 55385 | 55385 | 1 | 26 | Indel | 69.20% |
| A | 55384 | 55384 | 1 | 26 | Indel | 30.80% |
|  | 55384 | 55384 | 1 | 26 | Indel | 69.20% |
| T | 55383 | 55383 | 1 | 26 | Indel | 30.80% |
|  | 55383 | 55383 | 1 | 26 | Indel | 69.20% |
| C | 55382 | 55382 | 1 | 26 | Indel | 30.80% |
|  | 55382 | 55382 | 1 | 26 | Indel | 69.20% |
| T | 55381 | 55381 | 1 | 26 | Indel | 30.80% |
|  | 55381 | 55381 | 1 | 26 | Indel | 69.20% |
| T | 55380 | 55380 | 1 | 26 | Indel | 30.80% |
|  | 55380 | 55380 | 1 | 26 | Indel | 69.20% |
| T | 55379 | 55379 | 1 | 26 | Indel | 30.80% |
|  | 55379 | 55379 | 1 | 26 | Indel | 69.20% |
| G | 55378 | 55378 | 1 | 26 | Indel | 30.80% |
|  | 55378 | 55378 | 1 | 26 | Indel | 69.20% |
| T | 55377 | 55377 | 1 | 26 | Indel | 30.80% |
|  | 55377 | 55377 | 1 | 26 | Indel | 69.20% |
| A | 55376 | 55376 | 1 | 26 | Indel | 30.80% |
|  | 55376 | 55376 | 1 | 26 | Indel | 69.20% |
| T | 55375 | 55375 | 1 | 26 | Indel | 30.80% |
|  | 55375 | 55375 | 1 | 26 | Indel | 69.20% |
| A | 55374 | 55374 | 1 | 26 | Indel | 30.80% |
|  | 55374 | 55374 | 1 | 26 | Indel | 69.20% |
| T | 55373 | 55373 | 1 | 26 | Indel | 30.80% |
|  | 55373 | 55373 | 1 | 26 | Indel | 69.20% |
| T | 55372 | 55372 | 1 | 26 | Indel | 30.80% |
|  | 55372 | 55372 | 1 | 26 | Indel | 69.20% |
| A | 55371 | 55371 | 1 | 26 | Indel | 30.80% |
|  | 55371 | 55371 | 1 | 26 | Indel | 69.20% |
| G | 55370 | 55370 | 1 | 26 | Indel | 30.80% |
|  | 55370 | 55370 | 1 | 26 | Indel | 69.20% |
| A | 55369 | 55369 | 1 | 26 | Indel | 30.80% |
|  | 55369 | 55369 | 1 | 26 | Indel | 69.20% |
| T | 55368 | 55368 | 1 | 26 | Indel | 30.80% |
|  | 55368 | 55368 | 1 | 26 | Indel | 69.20% |
| A | 55367 | 55367 | 1 | 26 | Indel | 30.80% |
|  | 55367 | 55367 | 1 | 26 | Indel | 69.20% |
| T | 55366 | 55366 | 1 | 26 | Indel | 30.80% |
|  | 55366 | 55366 | 1 | 26 | Indel | 69.20% |
| A | 55365 | 55365 | 1 | 26 | Indel | 30.80% |
|  | 55365 | 55365 | 1 | 26 | Indel | 69.20% |
| A | 55364 | 55364 | 1 | 26 | Indel | 30.80% |
|  | 55364 | 55364 | 1 | 26 | Indel | 69.20% |
| A | 55363 | 55363 | 1 | 26 | Indel | 30.80% |
|  | 55363 | 55363 | 1 | 26 | Indel | 69.20% |
| A | 55362 | 55362 | 1 | 26 | Indel | 30.80% |
|  | 55362 | 55362 | 1 | 26 | Indel | 69.20% |
| T | 55361 | 55361 | 1 | 26 | Indel | 30.80% |
|  | 55361 | 55361 | 1 | 26 | Indel | 69.20% |
| A | 55360 | 55360 | 1 | 26 | Indel | 30.80% |
|  | 55360 | 55360 | 1 | 26 | Indel | 69.20% |
| T | 55359 | 55359 | 1 | 26 | Indel | 30.80% |
|  | 55359 | 55359 | 1 | 26 | Indel | 69.20% |
| C | 55358 | 55358 | 1 | 26 | Indel | 30.80% |
|  | 55358 | 55358 | 1 | 26 | Indel | 69.20% |
| T | 55357 | 55357 | 1 | 26 | Indel | 30.80% |
|  | 55357 | 55357 | 1 | 26 | Indel | 69.20% |
| A | 55356 | 55356 | 1 | 26 | Indel | 30.80% |
|  | 55356 | 55356 | 1 | 26 | Indel | 69.20% |
| A | 55355 | 55355 | 1 | 26 | Indel | 30.80% |
|  | 55355 | 55355 | 1 | 26 | Indel | 69.20% |
| A | 55354 | 55354 | 1 | 26 | Indel | 30.80% |
|  | 55354 | 55354 | 1 | 26 | Indel | 69.20% |
| G | 55353 | 55353 | 1 | 26 | Indel | 30.80% |
|  | 55353 | 55353 | 1 | 26 | Indel | 69.20% |
| A | 55352 | 55352 | 1 | 26 | Indel | 30.80% |
|  | 55352 | 55352 | 1 | 26 | Indel | 69.20% |
| T | 55351 | 55351 | 1 | 26 | Indel | 30.80% |
|  | 55351 | 55351 | 1 | 26 | Indel | 69.20% |
| A | 55350 | 55350 | 1 | 26 | Indel | 30.80% |
|  | 55350 | 55350 | 1 | 26 | Indel | 69.20% |
| A | 55349 | 55349 | 1 | 26 | Indel | 30.80% |
|  | 55349 | 55349 | 1 | 26 | Indel | 69.20% |
| A | 55348 | 55348 | 1 | 26 | Indel | 30.80% |
|  | 55348 | 55348 | 1 | 26 | Indel | 69.20% |
| A | 55347 | 55347 | 1 | 26 | Indel | 30.80% |
|  | 55347 | 55347 | 1 | 26 | Indel | 69.20% |
| G | 55346 | 55346 | 1 | 26 | Indel | 30.80% |
|  | 55346 | 55346 | 1 | 26 | Indel | 69.20% |
| A | 55345 | 55345 | 1 | 26 | Indel | 30.80% |
|  | 55345 | 55345 | 1 | 26 | Indel | 69.20% |
| T | 55344 | 55344 | 1 | 26 | Indel | 30.80% |
|  | 55344 | 55344 | 1 | 26 | Indel | 69.20% |
| A | 55343 | 55343 | 1 | 26 | Indel | 30.80% |
|  | 55343 | 55343 | 1 | 26 | Indel | 69.20% |
| A | 55342 | 55342 | 1 | 26 | Indel | 30.80% |
|  | 55342 | 55342 | 1 | 26 | Indel | 69.20% |
| A | 55341 | 55341 | 1 | 26 | Indel | 30.80% |
|  | 55341 | 55341 | 1 | 26 | Indel | 69.20% |
| T | 55340 | 55340 | 1 | 26 | Indel | 30.80% |
|  | 55340 | 55340 | 1 | 26 | Indel | 69.20% |
| A | 55339 | 55339 | 1 | 26 | Indel | 30.80% |
|  | 55339 | 55339 | 1 | 26 | Indel | 69.20% |
| T | 55338 | 55338 | 1 | 26 | Indel | 30.80% |
|  | 55338 | 55338 | 1 | 26 | Indel | 69.20% |
| T | 55337 | 55337 | 1 | 26 | Indel | 30.80% |
|  | 55337 | 55337 | 1 | 26 | Indel | 69.20% |
| T | 54556 | 54556 | 1 | 26 | Indel | 69.20% |
|  | 54556 | 54556 | 1 | 26 | Indel | 30.80% |
| A | 53125 | 53125 | 1 | 26 | Indel | 30.80% |
|  | 53125 | 53125 | 1 | 26 | Indel | 69.20% |
| T | 53106 | 53106 | 1 | 26 | Indel | 73.10% |
|  | 53106 | 53106 | 1 | 26 | Indel | 26.90% |
| A | 53105 | 53105 | 1 | 26 | Indel | 73.10% |
|  | 53105 | 53105 | 1 | 26 | Indel | 26.90% |
| T | 53104 | 53104 | 1 | 26 | Indel | 73.10% |
|  | 53104 | 53104 | 1 | 26 | Indel | 26.90% |
| T | 53103 | 53103 | 1 | 26 | Indel | 73.10% |
|  | 53103 | 53103 | 1 | 26 | Indel | 26.90% |
| T | 53102 | 53102 | 1 | 26 | Indel | 73.10% |
|  | 53102 | 53102 | 1 | 26 | Indel | 26.90% |
| G | 53101 | 53101 | 1 | 26 | Indel | 69.20% |
|  | 53101 | 53101 | 1 | 26 | Indel | 26.90% |
| T | 50948 | 50948 | 1 | 26 | Indel | 69.20% |
|  | 50948 | 50948 | 1 | 26 | Indel | 30.80% |
| T | 50947 | 50947 | 1 | 26 | Indel | 69.20% |
|  | 50947 | 50947 | 1 | 26 | Indel | 30.80% |
| G | 50946 | 50946 | 1 | 26 | Indel | 69.20% |
|  | 50946 | 50946 | 1 | 26 | Indel | 30.80% |
| T | 50945 | 50945 | 1 | 26 | Indel | 69.20% |
|  | 50945 | 50945 | 1 | 26 | Indel | 30.80% |
| A | 50944 | 50944 | 1 | 26 | Indel | 69.20% |
|  | 50944 | 50944 | 1 | 26 | Indel | 30.80% |
| A | 50943 | 50943 | 1 | 26 | Indel | 69.20% |
|  | 50943 | 50943 | 1 | 26 | Indel | 30.80% |
| T | 50942 | 50942 | 1 | 26 | Indel | 69.20% |
|  | 50942 | 50942 | 1 | 26 | Indel | 30.80% |
| A | 50941 | 50941 | 1 | 26 | Indel | 69.20% |
|  | 50941 | 50941 | 1 | 26 | Indel | 30.80% |
| T | 50940 | 50940 | 1 | 26 | Indel | 69.20% |
|  | 50940 | 50940 | 1 | 26 | Indel | 30.80% |
| T | 50939 | 50939 | 1 | 26 | Indel | 69.20% |
|  | 50939 | 50939 | 1 | 26 | Indel | 30.80% |
| G | 50938 | 50938 | 1 | 26 | Indel | 69.20% |
|  | 50938 | 50938 | 1 | 26 | Indel | 30.80% |
| T | 50937 | 50937 | 1 | 26 | Indel | 69.20% |
|  | 50937 | 50937 | 1 | 26 | Indel | 30.80% |
| A | 50936 | 50936 | 1 | 26 | Indel | 69.20% |
|  | 50936 | 50936 | 1 | 26 | Indel | 30.80% |
| T | 50935 | 50935 | 1 | 26 | Indel | 69.20% |
|  | 50935 | 50935 | 1 | 26 | Indel | 30.80% |
| T | 50934 | 50934 | 1 | 26 | Indel | 69.20% |
|  | 50934 | 50934 | 1 | 26 | Indel | 30.80% |
| G | 50933 | 50933 | 1 | 26 | Indel | 69.20% |
|  | 50933 | 50933 | 1 | 26 | Indel | 30.80% |
| T | 50932 | 50932 | 1 | 26 | Indel | 69.20% |
|  | 50932 | 50932 | 1 | 26 | Indel | 30.80% |
| T | 50931 | 50931 | 1 | 26 | Indel | 69.20% |
|  | 50931 | 50931 | 1 | 26 | Indel | 30.80% |
| T | 50930 | 50930 | 1 | 26 | Indel | 69.20% |
|  | 50930 | 50930 | 1 | 26 | Indel | 30.80% |
| T | 50929 | 50929 | 1 | 26 | Indel | 69.20% |
|  | 50929 | 50929 | 1 | 26 | Indel | 30.80% |
| T | 50928 | 50928 | 1 | 26 | Indel | 69.20% |
|  | 50928 | 50928 | 1 | 26 | Indel | 30.80% |
| T | 50927 | 50927 | 1 | 26 | Indel | 69.20% |
|  | 50927 | 50927 | 1 | 26 | Indel | 30.80% |
| T | 50926 | 50926 | 1 | 26 | Indel | 69.20% |
|  | 50926 | 50926 | 1 | 26 | Indel | 30.80% |
| T | 50680 | 50680 | 1 | 26 | Indel | 30.80% |
|  | 50680 | 50680 | 1 | 26 | Indel | 69.20% |
| A | 50679 | 50679 | 1 | 26 | Indel | 30.80% |
|  | 50679 | 50679 | 1 | 26 | Indel | 69.20% |
| A | 50678 | 50678 | 1 | 26 | Indel | 30.80% |
|  | 50678 | 50678 | 1 | 26 | Indel | 69.20% |
| T | 50677 | 50677 | 1 | 26 | Indel | 30.80% |
|  | 50677 | 50677 | 1 | 26 | Indel | 69.20% |
| G | 50676 | 50676 | 1 | 26 | Indel | 30.80% |
|  | 50676 | 50676 | 1 | 26 | Indel | 69.20% |
| T | 50675 | 50675 | 1 | 26 | Indel | 30.80% |
|  | 50675 | 50675 | 1 | 26 | Indel | 69.20% |
| A | 50674 | 50674 | 1 | 26 | Indel | 30.80% |
|  | 50674 | 50674 | 1 | 26 | Indel | 69.20% |
| A | 50673 | 50673 | 1 | 26 | Indel | 30.80% |
|  | 50673 | 50673 | 1 | 26 | Indel | 69.20% |
| T | 50672 | 50672 | 1 | 26 | Indel | 30.80% |
|  | 50672 | 50672 | 1 | 26 | Indel | 69.20% |
| G | 50671 | 50671 | 1 | 26 | Indel | 30.80% |
|  | 50671 | 50671 | 1 | 26 | Indel | 69.20% |
| A | 50670 | 50670 | 1 | 26 | Indel | 30.80% |
|  | 50670 | 50670 | 1 | 26 | Indel | 69.20% |
| A | 50669 | 50669 | 1 | 26 | Indel | 30.80% |
|  | 50669 | 50669 | 1 | 26 | Indel | 69.20% |
| A | 50668 | 50668 | 1 | 26 | Indel | 30.80% |
|  | 50668 | 50668 | 1 | 26 | Indel | 69.20% |
| T | 50667 | 50667 | 1 | 26 | Indel | 30.80% |
|  | 50667 | 50667 | 1 | 26 | Indel | 69.20% |
| A | 50404 | 50404 | 1 | 26 | Indel | 69.20% |
|  | 50404 | 50404 | 1 | 26 | Indel | 30.80% |
| T | 50403 | 50403 | 1 | 26 | Indel | 69.20% |
|  | 50403 | 50403 | 1 | 26 | Indel | 30.80% |
| A | 50402 | 50402 | 1 | 26 | Indel | 69.20% |
|  | 50402 | 50402 | 1 | 26 | Indel | 30.80% |
| T | 50401 | 50401 | 1 | 26 | Indel | 69.20% |
|  | 50401 | 50401 | 1 | 26 | Indel | 30.80% |
| A | 50400 | 50400 | 1 | 26 | Indel | 69.20% |
|  | 50400 | 50400 | 1 | 26 | Indel | 30.80% |
| A | 50399 | 50399 | 1 | 26 | Indel | 69.20% |
|  | 50399 | 50399 | 1 | 26 | Indel | 30.80% |
| T | 50398 | 50398 | 1 | 26 | Indel | 69.20% |
|  | 50398 | 50398 | 1 | 26 | Indel | 30.80% |
| T | 50397 | 50397 | 1 | 26 | Indel | 69.20% |
|  | 50397 | 50397 | 1 | 26 | Indel | 30.80% |
| A | 50396 | 50396 | 1 | 26 | Indel | 69.20% |
|  | 50396 | 50396 | 1 | 26 | Indel | 30.80% |
| T | 50395 | 50395 | 1 | 26 | Indel | 69.20% |
|  | 50395 | 50395 | 1 | 26 | Indel | 30.80% |
| C | 50394 | 50394 | 1 | 26 | Indel | 69.20% |
|  | 50394 | 50394 | 1 | 26 | Indel | 30.80% |
| C | 50393 | 50393 | 1 | 26 | Indel | 69.20% |
|  | 50393 | 50393 | 1 | 26 | Indel | 30.80% |
| A | 50392 | 50392 | 1 | 26 | Indel | 69.20% |
|  | 50392 | 50392 | 1 | 26 | Indel | 30.80% |
| T | 50391 | 50391 | 1 | 26 | Indel | 69.20% |
|  | 50391 | 50391 | 1 | 26 | Indel | 30.80% |
| A | 50390 | 50390 | 1 | 26 | Indel | 69.20% |
|  | 50390 | 50390 | 1 | 26 | Indel | 30.80% |
| C | 50389 | 50389 | 1 | 26 | Indel | 69.20% |
|  | 50389 | 50389 | 1 | 26 | Indel | 30.80% |
| A | 50388 | 50388 | 1 | 26 | Indel | 69.20% |
|  | 50388 | 50388 | 1 | 26 | Indel | 30.80% |
| A | 50387 | 50387 | 1 | 26 | Indel | 69.20% |
|  | 50387 | 50387 | 1 | 26 | Indel | 30.80% |
| G | 50386 | 50386 | 1 | 26 | Indel | 69.20% |
|  | 50386 | 50386 | 1 | 26 | Indel | 30.80% |
| A | 50385 | 50385 | 1 | 26 | Indel | 69.20% |
|  | 50385 | 50385 | 1 | 26 | Indel | 30.80% |
| A | 48328 | 48328 | 1 | 26 | Indel | 73.10% |
|  | 48328 | 48328 | 1 | 26 | Indel | 26.90% |
| T | 46964 | 46964 | 1 | 26 | Indel | 73.10% |
|  | 46964 | 46964 | 1 | 26 | Indel | 26.90% |
| A | 45977 | 45977 | 1 | 26 | Indel | 30.80% |
|  | 45977 | 45977 | 1 | 26 | Indel | 69.20% |
| A | 45976 | 45976 | 1 | 26 | Indel | 30.80% |
|  | 45976 | 45976 | 1 | 26 | Indel | 69.20% |
| T | 40087 | 40087 | 1 | 26 | Indel | 69.20% |
|  | 40087 | 40087 | 1 | 26 | Indel | 30.80% |
| C | 40086 | 40086 | 1 | 26 | Indel | 69.20% |
|  | 40086 | 40086 | 1 | 26 | Indel | 30.80% |
| A | 40085 | 40085 | 1 | 26 | Indel | 69.20% |
|  | 40085 | 40085 | 1 | 26 | Indel | 30.80% |
| A | 40084 | 40084 | 1 | 26 | Indel | 69.20% |
|  | 40084 | 40084 | 1 | 26 | Indel | 30.80% |
| A | 40083 | 40083 | 1 | 26 | Indel | 69.20% |
|  | 40083 | 40083 | 1 | 26 | Indel | 30.80% |
| T | 40082 | 40082 | 1 | 26 | Indel | 69.20% |
|  | 40082 | 40082 | 1 | 26 | Indel | 30.80% |
| C | 40081 | 40081 | 1 | 26 | Indel | 69.20% |
|  | 40081 | 40081 | 1 | 26 | Indel | 30.80% |
| A | 40080 | 40080 | 1 | 26 | Indel | 69.20% |
|  | 40080 | 40080 | 1 | 26 | Indel | 30.80% |
| T | 40079 | 40079 | 1 | 26 | Indel | 69.20% |
|  | 40079 | 40079 | 1 | 26 | Indel | 30.80% |
| A | 40078 | 40078 | 1 | 26 | Indel | 69.20% |
|  | 40078 | 40078 | 1 | 26 | Indel | 30.80% |
| A | 40077 | 40077 | 1 | 26 | Indel | 69.20% |
|  | 40077 | 40077 | 1 | 26 | Indel | 30.80% |
| A | 40076 | 40076 | 1 | 26 | Indel | 69.20% |
|  | 40076 | 40076 | 1 | 26 | Indel | 30.80% |
| T | 40075 | 40075 | 1 | 26 | Indel | 69.20% |
|  | 40075 | 40075 | 1 | 26 | Indel | 30.80% |
| T | 40074 | 40074 | 1 | 26 | Indel | 69.20% |
|  | 40074 | 40074 | 1 | 26 | Indel | 30.80% |
| A | 40073 | 40073 | 1 | 26 | Indel | 69.20% |
|  | 40073 | 40073 | 1 | 26 | Indel | 30.80% |
| T | 40072 | 40072 | 1 | 26 | Indel | 69.20% |
|  | 40072 | 40072 | 1 | 26 | Indel | 30.80% |
| T | 40071 | 40071 | 1 | 26 | Indel | 69.20% |
|  | 40071 | 40071 | 1 | 26 | Indel | 30.80% |
| A | 40070 | 40070 | 1 | 26 | Indel | 69.20% |
|  | 40070 | 40070 | 1 | 26 | Indel | 30.80% |
| A | 40069 | 40069 | 1 | 26 | Indel | 69.20% |
|  | 40069 | 40069 | 1 | 26 | Indel | 30.80% |
| A | 40068 | 40068 | 1 | 26 | Indel | 69.20% |
|  | 40068 | 40068 | 1 | 26 | Indel | 30.80% |
| T | 40067 | 40067 | 1 | 26 | Indel | 69.20% |
|  | 40067 | 40067 | 1 | 26 | Indel | 30.80% |
| A | 40066 | 40066 | 1 | 26 | Indel | 69.20% |
|  | 40066 | 40066 | 1 | 26 | Indel | 30.80% |
| A | 40065 | 40065 | 1 | 26 | Indel | 69.20% |
|  | 40065 | 40065 | 1 | 26 | Indel | 30.80% |
| A | 39847 | 39847 | 1 | 26 | Indel | 69.20% |
|  | 39847 | 39847 | 1 | 26 | Indel | 30.80% |
| T | 39846 | 39846 | 1 | 26 | Indel | 69.20% |
|  | 39846 | 39846 | 1 | 26 | Indel | 30.80% |
| T | 39845 | 39845 | 1 | 26 | Indel | 69.20% |
|  | 39845 | 39845 | 1 | 26 | Indel | 30.80% |
| A | 39844 | 39844 | 1 | 26 | Indel | 69.20% |
|  | 39844 | 39844 | 1 | 26 | Indel | 30.80% |
| T | 39843 | 39843 | 1 | 26 | Indel | 69.20% |
|  | 39843 | 39843 | 1 | 26 | Indel | 30.80% |
| A | 39842 | 39842 | 1 | 26 | Indel | 69.20% |
|  | 39842 | 39842 | 1 | 26 | Indel | 30.80% |
| T | 39841 | 39841 | 1 | 26 | Indel | 69.20% |
|  | 39841 | 39841 | 1 | 26 | Indel | 30.80% |
| G | 39840 | 39840 | 1 | 26 | Indel | 69.20% |
|  | 39840 | 39840 | 1 | 26 | Indel | 30.80% |
| T | 39839 | 39839 | 1 | 26 | Indel | 69.20% |
|  | 39839 | 39839 | 1 | 26 | Indel | 30.80% |
| T | 39838 | 39838 | 1 | 26 | Indel | 69.20% |
|  | 39838 | 39838 | 1 | 26 | Indel | 30.80% |
| A | 39837 | 39837 | 1 | 26 | Indel | 69.20% |
|  | 39837 | 39837 | 1 | 26 | Indel | 30.80% |
| T | 39836 | 39836 | 1 | 26 | Indel | 69.20% |
|  | 39836 | 39836 | 1 | 26 | Indel | 30.80% |
| A | 39835 | 39835 | 1 | 26 | Indel | 69.20% |
|  | 39835 | 39835 | 1 | 26 | Indel | 30.80% |
| T | 39834 | 39834 | 1 | 26 | Indel | 69.20% |
|  | 39834 | 39834 | 1 | 26 | Indel | 30.80% |
| A | 39833 | 39833 | 1 | 26 | Indel | 69.20% |
|  | 39833 | 39833 | 1 | 26 | Indel | 30.80% |
| T | 39832 | 39832 | 1 | 26 | Indel | 69.20% |
|  | 39832 | 39832 | 1 | 26 | Indel | 30.80% |
| G | 39831 | 39831 | 1 | 26 | Indel | 69.20% |
|  | 39831 | 39831 | 1 | 26 | Indel | 30.80% |
| T | 39830 | 39830 | 1 | 26 | Indel | 69.20% |
|  | 39830 | 39830 | 1 | 26 | Indel | 30.80% |
| T | 39829 | 39829 | 1 | 26 | Indel | 69.20% |
|  | 39829 | 39829 | 1 | 26 | Indel | 30.80% |
| A | 39828 | 39828 | 1 | 26 | Indel | 69.20% |
|  | 39828 | 39828 | 1 | 26 | Indel | 30.80% |
| G | 39827 | 39827 | 1 | 26 | Indel | 69.20% |
|  | 39827 | 39827 | 1 | 26 | Indel | 30.80% |
| G | 39826 | 39826 | 1 | 26 | Indel | 69.20% |
|  | 39826 | 39826 | 1 | 26 | Indel | 30.80% |
| T | 39825 | 39825 | 1 | 26 | Indel | 69.20% |
|  | 39825 | 39825 | 1 | 26 | Indel | 30.80% |
| A | 39824 | 39824 | 1 | 26 | Indel | 69.20% |
|  | 39824 | 39824 | 1 | 26 | Indel | 30.80% |
| T | 39823 | 39823 | 1 | 26 | Indel | 69.20% |
|  | 39823 | 39823 | 1 | 26 | Indel | 30.80% |
| T | 39317 | 39317 | 1 | 26 | Indel | 69.20% |
|  | 39317 | 39317 | 1 | 26 | Indel | 30.80% |
| T | 39316 | 39316 | 1 | 26 | Indel | 69.20% |
|  | 39316 | 39316 | 1 | 26 | Indel | 30.80% |
| T | 39315 | 39315 | 1 | 26 | Indel | 69.20% |
|  | 39315 | 39315 | 1 | 26 | Indel | 30.80% |
| T | 35759 | 35759 | 1 | 26 | Indel | 69.20% |
|  | 35759 | 35759 | 1 | 26 | Indel | 30.80% |
| T | 35758 | 35758 | 1 | 26 | Indel | 69.20% |
|  | 35758 | 35758 | 1 | 26 | Indel | 30.80% |
| T | 35757 | 35757 | 1 | 26 | Indel | 69.20% |
|  | 35757 | 35757 | 1 | 26 | Indel | 30.80% |
| A | 35756 | 35756 | 1 | 26 | Indel | 69.20% |
|  | 35756 | 35756 | 1 | 26 | Indel | 30.80% |
| A | 35755 | 35755 | 1 | 26 | Indel | 69.20% |
|  | 35755 | 35755 | 1 | 26 | Indel | 30.80% |
| T | 35754 | 35754 | 1 | 26 | Indel | 69.20% |
|  | 35754 | 35754 | 1 | 26 | Indel | 30.80% |
| T | 35753 | 35753 | 1 | 26 | Indel | 69.20% |
|  | 35753 | 35753 | 1 | 26 | Indel | 30.80% |
| A | 35752 | 35752 | 1 | 26 | Indel | 69.20% |
|  | 35752 | 35752 | 1 | 26 | Indel | 30.80% |
| T | 35751 | 35751 | 1 | 26 | Indel | 69.20% |
|  | 35751 | 35751 | 1 | 26 | Indel | 30.80% |
| A | 35750 | 35750 | 1 | 26 | Indel | 69.20% |
|  | 35750 | 35750 | 1 | 26 | Indel | 30.80% |
| T | 35749 | 35749 | 1 | 26 | Indel | 69.20% |
|  | 35749 | 35749 | 1 | 26 | Indel | 30.80% |
| A | 35748 | 35748 | 1 | 26 | Indel | 69.20% |
|  | 35748 | 35748 | 1 | 26 | Indel | 30.80% |
| A | 35747 | 35747 | 1 | 26 | Indel | 69.20% |
|  | 35747 | 35747 | 1 | 26 | Indel | 30.80% |
| C | 35746 | 35746 | 1 | 26 | Indel | 69.20% |
|  | 35746 | 35746 | 1 | 26 | Indel | 30.80% |
| A | 35745 | 35745 | 1 | 26 | Indel | 69.20% |
|  | 35745 | 35745 | 1 | 26 | Indel | 30.80% |
| T | 35744 | 35744 | 1 | 26 | Indel | 69.20% |
|  | 35744 | 35744 | 1 | 26 | Indel | 30.80% |
| A | 35743 | 35743 | 1 | 26 | Indel | 69.20% |
|  | 35743 | 35743 | 1 | 26 | Indel | 30.80% |
| A | 35742 | 35742 | 1 | 26 | Indel | 69.20% |
|  | 35742 | 35742 | 1 | 26 | Indel | 30.80% |
| C | 35741 | 35741 | 1 | 26 | Indel | 69.20% |
|  | 35741 | 35741 | 1 | 26 | Indel | 30.80% |
| T | 35740 | 35740 | 1 | 26 | Indel | 69.20% |
|  | 35740 | 35740 | 1 | 26 | Indel | 30.80% |
| T | 35739 | 35739 | 1 | 26 | Indel | 69.20% |
|  | 35739 | 35739 | 1 | 26 | Indel | 30.80% |
| A | 35738 | 35738 | 1 | 26 | Indel | 69.20% |
|  | 35738 | 35738 | 1 | 26 | Indel | 30.80% |
| A | 35737 | 35737 | 1 | 26 | Indel | 69.20% |
|  | 35737 | 35737 | 1 | 26 | Indel | 30.80% |
| T | 35736 | 35736 | 1 | 26 | Indel | 69.20% |
|  | 35736 | 35736 | 1 | 26 | Indel | 30.80% |
| T | 35735 | 35735 | 1 | 26 | Indel | 69.20% |
|  | 35735 | 35735 | 1 | 26 | Indel | 30.80% |
| T | 35734 | 35734 | 1 | 26 | Indel | 69.20% |
|  | 35734 | 35734 | 1 | 26 | Indel | 30.80% |
| A | 35733 | 35733 | 1 | 26 | Indel | 69.20% |
|  | 35733 | 35733 | 1 | 26 | Indel | 30.80% |
| T | 35732 | 35732 | 1 | 26 | Indel | 69.20% |
|  | 35732 | 35732 | 1 | 26 | Indel | 30.80% |
| G | 35731 | 35731 | 1 | 26 | Indel | 69.20% |
|  | 35731 | 35731 | 1 | 26 | Indel | 30.80% |
| T | 35730 | 35730 | 1 | 26 | Indel | 69.20% |
|  | 35730 | 35730 | 1 | 26 | Indel | 30.80% |
| A | 35729 | 35729 | 1 | 26 | Indel | 69.20% |
|  | 35729 | 35729 | 1 | 26 | Indel | 30.80% |
| T | 35728 | 35728 | 1 | 26 | Indel | 69.20% |
|  | 35728 | 35728 | 1 | 26 | Indel | 30.80% |
| G | 35727 | 35727 | 1 | 26 | Indel | 69.20% |
|  | 35727 | 35727 | 1 | 26 | Indel | 30.80% |
| A | 35726 | 35726 | 1 | 26 | Indel | 69.20% |
|  | 35726 | 35726 | 1 | 26 | Indel | 30.80% |
| T | 35725 | 35725 | 1 | 26 | Indel | 69.20% |
|  | 35725 | 35725 | 1 | 26 | Indel | 30.80% |
| A | 35724 | 35724 | 1 | 26 | Indel | 69.20% |
|  | 35724 | 35724 | 1 | 26 | Indel | 30.80% |
| A | 35723 | 35723 | 1 | 26 | Indel | 69.20% |
|  | 35723 | 35723 | 1 | 26 | Indel | 30.80% |
| T | 35722 | 35722 | 1 | 26 | Indel | 69.20% |
|  | 35722 | 35722 | 1 | 26 | Indel | 30.80% |
| A | 35721 | 35721 | 1 | 26 | Indel | 69.20% |
|  | 35721 | 35721 | 1 | 26 | Indel | 30.80% |
| A | 35720 | 35720 | 1 | 26 | Indel | 69.20% |
|  | 35720 | 35720 | 1 | 26 | Indel | 30.80% |
| A | 35114 | 35114 | 1 | 26 | Indel | 30.80% |
|  | 35114 | 35114 | 1 | 26 | Indel | 69.20% |
| A | 35113 | 35113 | 1 | 26 | Indel | 30.80% |
|  | 35113 | 35113 | 1 | 26 | Indel | 69.20% |
| T | 35112 | 35112 | 1 | 26 | Indel | 30.80% |
|  | 35112 | 35112 | 1 | 26 | Indel | 69.20% |
| A | 35111 | 35111 | 1 | 26 | Indel | 30.80% |
|  | 35111 | 35111 | 1 | 26 | Indel | 69.20% |
| A | 35110 | 35110 | 1 | 26 | Indel | 30.80% |
|  | 35110 | 35110 | 1 | 26 | Indel | 69.20% |
| T | 35109 | 35109 | 1 | 26 | Indel | 30.80% |
|  | 35109 | 35109 | 1 | 26 | Indel | 69.20% |
| A | 35108 | 35108 | 1 | 26 | Indel | 30.80% |
|  | 35108 | 35108 | 1 | 26 | Indel | 69.20% |
| T | 35107 | 35107 | 1 | 26 | Indel | 30.80% |
|  | 35107 | 35107 | 1 | 26 | Indel | 69.20% |
| T | 35106 | 35106 | 1 | 26 | Indel | 30.80% |
|  | 35106 | 35106 | 1 | 26 | Indel | 69.20% |
| A | 35105 | 35105 | 1 | 26 | Indel | 30.80% |
|  | 35105 | 35105 | 1 | 26 | Indel | 69.20% |
| T | 35104 | 35104 | 1 | 26 | Indel | 30.80% |
|  | 35104 | 35104 | 1 | 26 | Indel | 69.20% |
| A | 35103 | 35103 | 1 | 26 | Indel | 30.80% |
|  | 35103 | 35103 | 1 | 26 | Indel | 69.20% |
| A | 35102 | 35102 | 1 | 26 | Indel | 30.80% |
|  | 35102 | 35102 | 1 | 26 | Indel | 69.20% |
| A | 35101 | 35101 | 1 | 26 | Indel | 30.80% |
|  | 35101 | 35101 | 1 | 26 | Indel | 69.20% |
| T | 35100 | 35100 | 1 | 26 | Indel | 30.80% |
|  | 35100 | 35100 | 1 | 26 | Indel | 69.20% |
| T | 35099 | 35099 | 1 | 26 | Indel | 30.80% |
|  | 35099 | 35099 | 1 | 26 | Indel | 69.20% |
| A | 35098 | 35098 | 1 | 26 | Indel | 30.80% |
|  | 35098 | 35098 | 1 | 26 | Indel | 69.20% |
| T | 35097 | 35097 | 1 | 26 | Indel | 30.80% |
|  | 35097 | 35097 | 1 | 26 | Indel | 69.20% |
| A | 35096 | 35096 | 1 | 26 | Indel | 30.80% |
|  | 35096 | 35096 | 1 | 26 | Indel | 69.20% |
| T | 35095 | 35095 | 1 | 26 | Indel | 30.80% |
|  | 35095 | 35095 | 1 | 26 | Indel | 69.20% |
| T | 35094 | 35094 | 1 | 26 | Indel | 30.80% |
|  | 35094 | 35094 | 1 | 26 | Indel | 69.20% |
| A | 35093 | 35093 | 1 | 26 | Indel | 30.80% |
|  | 35093 | 35093 | 1 | 26 | Indel | 69.20% |
| T | 35092 | 35092 | 1 | 26 | Indel | 30.80% |
|  | 35092 | 35092 | 1 | 26 | Indel | 69.20% |
| A | 35091 | 35091 | 1 | 26 | Indel | 30.80% |
|  | 35091 | 35091 | 1 | 26 | Indel | 69.20% |
| A | 35090 | 35090 | 1 | 26 | Indel | 30.80% |
|  | 35090 | 35090 | 1 | 26 | Indel | 69.20% |
| A | 35089 | 35089 | 1 | 26 | Indel | 30.80% |
|  | 35089 | 35089 | 1 | 26 | Indel | 69.20% |
| T | 35088 | 35088 | 1 | 26 | Indel | 30.80% |
|  | 35088 | 35088 | 1 | 26 | Indel | 69.20% |
| A | 35087 | 35087 | 1 | 26 | Indel | 30.80% |
|  | 35087 | 35087 | 1 | 26 | Indel | 69.20% |
| T | 35086 | 35086 | 1 | 26 | Indel | 30.80% |
|  | 35086 | 35086 | 1 | 26 | Indel | 69.20% |
| T | 35085 | 35085 | 1 | 26 | Indel | 30.80% |
|  | 35085 | 35085 | 1 | 26 | Indel | 69.20% |
| T | 35084 | 35084 | 1 | 26 | Indel | 30.80% |
|  | 35084 | 35084 | 1 | 26 | Indel | 69.20% |
| A | 35083 | 35083 | 1 | 26 | Indel | 30.80% |
|  | 35083 | 35083 | 1 | 26 | Indel | 69.20% |
| A | 35082 | 35082 | 1 | 26 | Indel | 30.80% |
|  | 35082 | 35082 | 1 | 26 | Indel | 69.20% |
| A | 35081 | 35081 | 1 | 26 | Indel | 30.80% |
|  | 35081 | 35081 | 1 | 26 | Indel | 69.20% |
| T | 35080 | 35080 | 1 | 26 | Indel | 30.80% |
|  | 35080 | 35080 | 1 | 26 | Indel | 69.20% |
| T | 35079 | 35079 | 1 | 26 | Indel | 30.80% |
|  | 35079 | 35079 | 1 | 26 | Indel | 69.20% |
| A | 35078 | 35078 | 1 | 26 | Indel | 30.80% |
|  | 35078 | 35078 | 1 | 26 | Indel | 69.20% |
| T | 35077 | 35077 | 1 | 26 | Indel | 30.80% |
|  | 35077 | 35077 | 1 | 26 | Indel | 69.20% |
| A | 35076 | 35076 | 1 | 26 | Indel | 30.80% |
|  | 35076 | 35076 | 1 | 26 | Indel | 69.20% |
| T | 35075 | 35075 | 1 | 26 | Indel | 30.80% |
|  | 35075 | 35075 | 1 | 26 | Indel | 69.20% |
| T | 35074 | 35074 | 1 | 26 | Indel | 30.80% |
|  | 35074 | 35074 | 1 | 26 | Indel | 69.20% |
| A | 35073 | 35073 | 1 | 26 | Indel | 30.80% |
|  | 35073 | 35073 | 1 | 26 | Indel | 69.20% |
| T | 35072 | 35072 | 1 | 26 | Indel | 30.80% |
|  | 35072 | 35072 | 1 | 26 | Indel | 69.20% |
| A | 35071 | 35071 | 1 | 26 | Indel | 30.80% |
|  | 35071 | 35071 | 1 | 26 | Indel | 69.20% |
| A | 35070 | 35070 | 1 | 26 | Indel | 30.80% |
|  | 35070 | 35070 | 1 | 26 | Indel | 69.20% |
| A | 35069 | 35069 | 1 | 26 | Indel | 30.80% |
|  | 35069 | 35069 | 1 | 26 | Indel | 69.20% |
| T | 35068 | 35068 | 1 | 26 | Indel | 30.80% |
|  | 35068 | 35068 | 1 | 26 | Indel | 69.20% |
| A | 35067 | 35067 | 1 | 26 | Indel | 30.80% |
|  | 35067 | 35067 | 1 | 26 | Indel | 69.20% |
| T | 35066 | 35066 | 1 | 26 | Indel | 30.80% |
|  | 35066 | 35066 | 1 | 26 | Indel | 69.20% |
| T | 35065 | 35065 | 1 | 26 | Indel | 30.80% |
|  | 35065 | 35065 | 1 | 26 | Indel | 69.20% |
| A | 35064 | 35064 | 1 | 26 | Indel | 30.80% |
|  | 35064 | 35064 | 1 | 26 | Indel | 69.20% |
| T | 35063 | 35063 | 1 | 26 | Indel | 30.80% |
|  | 35063 | 35063 | 1 | 26 | Indel | 69.20% |
| T | 35062 | 35062 | 1 | 26 | Indel | 30.80% |
|  | 35062 | 35062 | 1 | 26 | Indel | 69.20% |
| A | 35061 | 35061 | 1 | 26 | Indel | 30.80% |
|  | 35061 | 35061 | 1 | 26 | Indel | 69.20% |
| A | 35060 | 35060 | 1 | 26 | Indel | 30.80% |
|  | 35060 | 35060 | 1 | 26 | Indel | 69.20% |
| T | 35059 | 35059 | 1 | 26 | Indel | 30.80% |
|  | 35059 | 35059 | 1 | 26 | Indel | 69.20% |
| T | 35058 | 35058 | 1 | 26 | Indel | 30.80% |
|  | 35058 | 35058 | 1 | 26 | Indel | 69.20% |
| A | 35057 | 35057 | 1 | 26 | Indel | 30.80% |
|  | 35057 | 35057 | 1 | 26 | Indel | 69.20% |
| A | 35056 | 35056 | 1 | 26 | Indel | 30.80% |
|  | 35056 | 35056 | 1 | 26 | Indel | 69.20% |
| A | 35055 | 35055 | 1 | 26 | Indel | 30.80% |
|  | 35055 | 35055 | 1 | 26 | Indel | 69.20% |
| T | 35054 | 35054 | 1 | 26 | Indel | 30.80% |
|  | 35054 | 35054 | 1 | 26 | Indel | 69.20% |
| T | 35053 | 35053 | 1 | 26 | Indel | 30.80% |
|  | 35053 | 35053 | 1 | 26 | Indel | 69.20% |
| A | 35052 | 35052 | 1 | 26 | Indel | 30.80% |
|  | 35052 | 35052 | 1 | 26 | Indel | 69.20% |
| T | 35051 | 35051 | 1 | 26 | Indel | 30.80% |
|  | 35051 | 35051 | 1 | 26 | Indel | 69.20% |
| A | 35050 | 35050 | 1 | 26 | Indel | 30.80% |
|  | 35050 | 35050 | 1 | 26 | Indel | 69.20% |
| A | 35049 | 35049 | 1 | 26 | Indel | 30.80% |
|  | 35049 | 35049 | 1 | 26 | Indel | 69.20% |
| A | 35048 | 35048 | 1 | 26 | Indel | 30.80% |
|  | 35048 | 35048 | 1 | 26 | Indel | 69.20% |
| A | 35047 | 35047 | 1 | 26 | Indel | 30.80% |
|  | 35047 | 35047 | 1 | 26 | Indel | 69.20% |
| T | 35046 | 35046 | 1 | 26 | Indel | 30.80% |
|  | 35046 | 35046 | 1 | 26 | Indel | 69.20% |
| T | 35045 | 35045 | 1 | 26 | Indel | 30.80% |
|  | 35045 | 35045 | 1 | 26 | Indel | 69.20% |
| A | 35044 | 35044 | 1 | 26 | Indel | 30.80% |
|  | 35044 | 35044 | 1 | 26 | Indel | 69.20% |
| T | 35043 | 35043 | 1 | 26 | Indel | 30.80% |
|  | 35043 | 35043 | 1 | 26 | Indel | 69.20% |
| A | 35042 | 35042 | 1 | 26 | Indel | 30.80% |
|  | 35042 | 35042 | 1 | 26 | Indel | 69.20% |
| T | 35041 | 35041 | 1 | 26 | Indel | 30.80% |
|  | 35041 | 35041 | 1 | 26 | Indel | 69.20% |
| T | 35040 | 35040 | 1 | 26 | Indel | 30.80% |
|  | 35040 | 35040 | 1 | 26 | Indel | 69.20% |
| A | 35039 | 35039 | 1 | 26 | Indel | 30.80% |
|  | 35039 | 35039 | 1 | 26 | Indel | 69.20% |
| A | 35038 | 35038 | 1 | 26 | Indel | 30.80% |
|  | 35038 | 35038 | 1 | 26 | Indel | 69.20% |
| A | 35037 | 35037 | 1 | 26 | Indel | 30.80% |
|  | 35037 | 35037 | 1 | 26 | Indel | 69.20% |
| A | 35036 | 35036 | 1 | 26 | Indel | 30.80% |
|  | 35036 | 35036 | 1 | 26 | Indel | 69.20% |
| A | 35035 | 35035 | 1 | 26 | Indel | 30.80% |
|  | 35035 | 35035 | 1 | 26 | Indel | 69.20% |
| T | 35034 | 35034 | 1 | 26 | Indel | 30.80% |
|  | 35034 | 35034 | 1 | 26 | Indel | 69.20% |
| T | 35033 | 35033 | 1 | 26 | Indel | 30.80% |
|  | 35033 | 35033 | 1 | 26 | Indel | 69.20% |
| A | 35032 | 35032 | 1 | 26 | Indel | 30.80% |
|  | 35032 | 35032 | 1 | 26 | Indel | 69.20% |
| T | 35031 | 35031 | 1 | 26 | Indel | 30.80% |
|  | 35031 | 35031 | 1 | 26 | Indel | 69.20% |
| A | 35030 | 35030 | 1 | 26 | Indel | 30.80% |
|  | 35030 | 35030 | 1 | 26 | Indel | 69.20% |
| T | 35029 | 35029 | 1 | 26 | Indel | 30.80% |
|  | 35029 | 35029 | 1 | 26 | Indel | 69.20% |
| T | 35028 | 35028 | 1 | 26 | Indel | 30.80% |
|  | 35028 | 35028 | 1 | 26 | Indel | 69.20% |
| A | 35027 | 35027 | 1 | 26 | Indel | 30.80% |
|  | 35027 | 35027 | 1 | 26 | Indel | 69.20% |
| A | 35026 | 35026 | 1 | 26 | Indel | 30.80% |
|  | 35026 | 35026 | 1 | 26 | Indel | 69.20% |
| A | 35025 | 35025 | 1 | 26 | Indel | 30.80% |
|  | 35025 | 35025 | 1 | 26 | Indel | 69.20% |
| A | 35024 | 35024 | 1 | 26 | Indel | 30.80% |
|  | 35024 | 35024 | 1 | 26 | Indel | 69.20% |
| A | 35023 | 35023 | 1 | 26 | Indel | 30.80% |
|  | 35023 | 35023 | 1 | 26 | Indel | 69.20% |
| A | 35022 | 35022 | 1 | 26 | Indel | 30.80% |
|  | 35022 | 35022 | 1 | 26 | Indel | 69.20% |
| A | 35021 | 35021 | 1 | 26 | Indel | 30.80% |
|  | 35021 | 35021 | 1 | 26 | Indel | 69.20% |
| T | 35020 | 35020 | 1 | 26 | Indel | 30.80% |
|  | 35020 | 35020 | 1 | 26 | Indel | 69.20% |
| T | 35019 | 35019 | 1 | 26 | Indel | 30.80% |
|  | 35019 | 35019 | 1 | 26 | Indel | 69.20% |
| A | 35018 | 35018 | 1 | 26 | Indel | 30.80% |
|  | 35018 | 35018 | 1 | 26 | Indel | 69.20% |
| T | 35017 | 35017 | 1 | 26 | Indel | 30.80% |
|  | 35017 | 35017 | 1 | 26 | Indel | 69.20% |
| A | 35016 | 35016 | 1 | 26 | Indel | 30.80% |
|  | 35016 | 35016 | 1 | 26 | Indel | 69.20% |
| T | 35015 | 35015 | 1 | 26 | Indel | 30.80% |
|  | 35015 | 35015 | 1 | 26 | Indel | 69.20% |
| T | 35014 | 35014 | 1 | 26 | Indel | 30.80% |
|  | 35014 | 35014 | 1 | 26 | Indel | 69.20% |
| A | 35013 | 35013 | 1 | 26 | Indel | 30.80% |
|  | 35013 | 35013 | 1 | 26 | Indel | 69.20% |
| A | 35012 | 35012 | 1 | 26 | Indel | 30.80% |
|  | 35012 | 35012 | 1 | 26 | Indel | 69.20% |
| T | 35011 | 35011 | 1 | 26 | Indel | 30.80% |
|  | 35011 | 35011 | 1 | 26 | Indel | 69.20% |
| A | 35010 | 35010 | 1 | 26 | Indel | 30.80% |
|  | 35010 | 35010 | 1 | 26 | Indel | 69.20% |
| T | 35009 | 35009 | 1 | 26 | Indel | 30.80% |
|  | 35009 | 35009 | 1 | 26 | Indel | 69.20% |
| T | 35008 | 35008 | 1 | 26 | Indel | 30.80% |
|  | 35008 | 35008 | 1 | 26 | Indel | 69.20% |
| A | 35007 | 35007 | 1 | 26 | Indel | 30.80% |
|  | 35007 | 35007 | 1 | 26 | Indel | 69.20% |
| T | 35006 | 35006 | 1 | 26 | Indel | 30.80% |
|  | 35006 | 35006 | 1 | 26 | Indel | 69.20% |
| T | 34989 | 34989 | 1 | 26 | Indel | 30.80% |
|  | 34989 | 34989 | 1 | 26 | Indel | 69.20% |
| A | 34988 | 34988 | 1 | 26 | Indel | 30.80% |
|  | 34988 | 34988 | 1 | 26 | Indel | 69.20% |
| A | 34987 | 34987 | 1 | 26 | Indel | 30.80% |
|  | 34987 | 34987 | 1 | 26 | Indel | 69.20% |
| T | 34986 | 34986 | 1 | 26 | Indel | 30.80% |
|  | 34986 | 34986 | 1 | 26 | Indel | 69.20% |
| T | 34985 | 34985 | 1 | 26 | Indel | 30.80% |
|  | 34985 | 34985 | 1 | 26 | Indel | 69.20% |
| A | 34984 | 34984 | 1 | 26 | Indel | 30.80% |
|  | 34984 | 34984 | 1 | 26 | Indel | 69.20% |
| A | 34983 | 34983 | 1 | 26 | Indel | 30.80% |
|  | 34983 | 34983 | 1 | 26 | Indel | 69.20% |
| A | 34982 | 34982 | 1 | 26 | Indel | 30.80% |
|  | 34982 | 34982 | 1 | 26 | Indel | 69.20% |
| T | 34981 | 34981 | 1 | 26 | Indel | 30.80% |
|  | 34981 | 34981 | 1 | 26 | Indel | 69.20% |
| T | 34980 | 34980 | 1 | 26 | Indel | 30.80% |
|  | 34980 | 34980 | 1 | 26 | Indel | 69.20% |
| A | 34979 | 34979 | 1 | 26 | Indel | 30.80% |
|  | 34979 | 34979 | 1 | 26 | Indel | 69.20% |
| T | 34978 | 34978 | 1 | 26 | Indel | 30.80% |
|  | 34978 | 34978 | 1 | 26 | Indel | 69.20% |
| A | 34977 | 34977 | 1 | 26 | Indel | 30.80% |
|  | 34977 | 34977 | 1 | 26 | Indel | 69.20% |
| T | 34976 | 34976 | 1 | 26 | Indel | 30.80% |
|  | 34976 | 34976 | 1 | 26 | Indel | 69.20% |
| T | 34975 | 34975 | 1 | 26 | Indel | 30.80% |
|  | 34975 | 34975 | 1 | 26 | Indel | 69.20% |
| A | 34974 | 34974 | 1 | 26 | Indel | 30.80% |
|  | 34974 | 34974 | 1 | 26 | Indel | 69.20% |
| T | 34973 | 34973 | 1 | 26 | Indel | 30.80% |
|  | 34973 | 34973 | 1 | 26 | Indel | 69.20% |
| A | 34972 | 34972 | 1 | 26 | Indel | 30.80% |
|  | 34972 | 34972 | 1 | 26 | Indel | 69.20% |
| A | 34971 | 34971 | 1 | 26 | Indel | 30.80% |
|  | 34971 | 34971 | 1 | 26 | Indel | 69.20% |
| A | 34970 | 34970 | 1 | 26 | Indel | 30.80% |
|  | 34970 | 34970 | 1 | 26 | Indel | 69.20% |
| T | 34969 | 34969 | 1 | 26 | Indel | 30.80% |
|  | 34969 | 34969 | 1 | 26 | Indel | 69.20% |
| A | 34968 | 34968 | 1 | 26 | Indel | 30.80% |
|  | 34968 | 34968 | 1 | 26 | Indel | 69.20% |
| T | 34967 | 34967 | 1 | 26 | Indel | 30.80% |
|  | 34967 | 34967 | 1 | 26 | Indel | 69.20% |
| T | 34966 | 34966 | 1 | 26 | Indel | 30.80% |
|  | 34966 | 34966 | 1 | 26 | Indel | 69.20% |
| A | 34965 | 34965 | 1 | 26 | Indel | 30.80% |
|  | 34965 | 34965 | 1 | 26 | Indel | 69.20% |
| A | 34964 | 34964 | 1 | 26 | Indel | 30.80% |
|  | 34964 | 34964 | 1 | 26 | Indel | 69.20% |
| A | 34963 | 34963 | 1 | 26 | Indel | 30.80% |
|  | 34963 | 34963 | 1 | 26 | Indel | 69.20% |
| T | 34962 | 34962 | 1 | 26 | Indel | 30.80% |
|  | 34962 | 34962 | 1 | 26 | Indel | 69.20% |
| T | 34961 | 34961 | 1 | 26 | Indel | 30.80% |
|  | 34961 | 34961 | 1 | 26 | Indel | 69.20% |
| A | 34960 | 34960 | 1 | 26 | Indel | 30.80% |
|  | 34960 | 34960 | 1 | 26 | Indel | 69.20% |
| T | 34959 | 34959 | 1 | 26 | Indel | 30.80% |
|  | 34959 | 34959 | 1 | 26 | Indel | 69.20% |
| A | 34958 | 34958 | 1 | 26 | Indel | 30.80% |
|  | 34958 | 34958 | 1 | 26 | Indel | 69.20% |
| T | 34957 | 34957 | 1 | 26 | Indel | 30.80% |
|  | 34957 | 34957 | 1 | 26 | Indel | 69.20% |
| T | 34956 | 34956 | 1 | 26 | Indel | 30.80% |
|  | 34956 | 34956 | 1 | 26 | Indel | 69.20% |
| A | 34955 | 34955 | 1 | 26 | Indel | 30.80% |
|  | 34955 | 34955 | 1 | 26 | Indel | 69.20% |
| T | 34954 | 34954 | 1 | 26 | Indel | 30.80% |
|  | 34954 | 34954 | 1 | 26 | Indel | 69.20% |
| A | 34953 | 34953 | 1 | 26 | Indel | 30.80% |
|  | 34953 | 34953 | 1 | 26 | Indel | 69.20% |
| A | 34952 | 34952 | 1 | 26 | Indel | 30.80% |
|  | 34952 | 34952 | 1 | 26 | Indel | 69.20% |
| A | 34951 | 34951 | 1 | 26 | Indel | 30.80% |
|  | 34951 | 34951 | 1 | 26 | Indel | 69.20% |
| T | 34950 | 34950 | 1 | 26 | Indel | 30.80% |
|  | 34950 | 34950 | 1 | 26 | Indel | 69.20% |
| A | 34908 | 34908 | 1 | 26 | Indel | 30.80% |
|  | 34908 | 34908 | 1 | 26 | Indel | 65.40% |
| T | 34907 | 34907 | 1 | 26 | Indel | 34.60% |
|  | 34907 | 34907 | 1 | 26 | Indel | 65.40% |
| A | 34906 | 34906 | 1 | 26 | Indel | 34.60% |
|  | 34906 | 34906 | 1 | 26 | Indel | 65.40% |
| A | 34905 | 34905 | 1 | 26 | Indel | 30.80% |
|  | 34905 | 34905 | 1 | 26 | Indel | 69.20% |
| A | 34904 | 34904 | 1 | 26 | Indel | 26.90% |
|  | 34904 | 34904 | 1 | 26 | Indel | 69.20% |
| T | 34903 | 34903 | 1 | 26 | Indel | 30.80% |
|  | 34903 | 34903 | 1 | 26 | Indel | 69.20% |
| A | 34902 | 34902 | 1 | 26 | Indel | 30.80% |
|  | 34902 | 34902 | 1 | 26 | Indel | 69.20% |
| T | 34901 | 34901 | 1 | 26 | Indel | 34.60% |
|  | 34901 | 34901 | 1 | 26 | Indel | 65.40% |
| A | 34900 | 34900 | 1 | 26 | Indel | 30.80% |
|  | 34900 | 34900 | 1 | 26 | Indel | 69.20% |
| A | 34899 | 34899 | 1 | 26 | Indel | 30.80% |
|  | 34899 | 34899 | 1 | 26 | Indel | 69.20% |
| T | 34898 | 34898 | 1 | 26 | Indel | 30.80% |
|  | 34898 | 34898 | 1 | 26 | Indel | 69.20% |
| A | 34897 | 34897 | 1 | 26 | Indel | 30.80% |
|  | 34897 | 34897 | 1 | 26 | Indel | 69.20% |
| A | 34896 | 34896 | 1 | 26 | Indel | 30.80% |
|  | 34896 | 34896 | 1 | 26 | Indel | 69.20% |
| A | 34895 | 34895 | 1 | 26 | Indel | 30.80% |
|  | 34895 | 34895 | 1 | 26 | Indel | 69.20% |
| T | 34894 | 34894 | 1 | 26 | Indel | 30.80% |
|  | 34894 | 34894 | 1 | 26 | Indel | 69.20% |
| A | 34893 | 34893 | 1 | 26 | Indel | 30.80% |
|  | 34893 | 34893 | 1 | 26 | Indel | 69.20% |
| A | 34892 | 34892 | 1 | 26 | Indel | 30.80% |
|  | 34892 | 34892 | 1 | 26 | Indel | 69.20% |
| A | 34891 | 34891 | 1 | 26 | Indel | 30.80% |
|  | 34891 | 34891 | 1 | 26 | Indel | 69.20% |
| T | 34890 | 34890 | 1 | 26 | Indel | 30.80% |
|  | 34890 | 34890 | 1 | 26 | Indel | 69.20% |
| T | 34889 | 34889 | 1 | 26 | Indel | 30.80% |
|  | 34889 | 34889 | 1 | 26 | Indel | 69.20% |
| A | 34888 | 34888 | 1 | 26 | Indel | 30.80% |
|  | 34888 | 34888 | 1 | 26 | Indel | 69.20% |
| A | 34887 | 34887 | 1 | 26 | Indel | 26.90% |
|  | 34887 | 34887 | 1 | 26 | Indel | 73.10% |
| T | 34886 | 34886 | 1 | 26 | Indel | 26.90% |
|  | 34886 | 34886 | 1 | 26 | Indel | 73.10% |
| A | 34885 | 34885 | 1 | 26 | Indel | 26.90% |
|  | 34885 | 34885 | 1 | 26 | Indel | 73.10% |
| T | 34884 | 34884 | 1 | 26 | Indel | 26.90% |
|  | 34884 | 34884 | 1 | 26 | Indel | 73.10% |
| A | 34883 | 34883 | 1 | 26 | Indel | 26.90% |
|  | 34883 | 34883 | 1 | 26 | Indel | 73.10% |
| A | 34882 | 34882 | 1 | 26 | Indel | 26.90% |
|  | 34882 | 34882 | 1 | 26 | Indel | 73.10% |
| T | 34881 | 34881 | 1 | 26 | Indel | 26.90% |
|  | 34881 | 34881 | 1 | 26 | Indel | 73.10% |
| A | 34880 | 34880 | 1 | 26 | Indel | 26.90% |
|  | 34880 | 34880 | 1 | 26 | Indel | 73.10% |
| A | 34879 | 34879 | 1 | 26 | Indel | 26.90% |
|  | 34879 | 34879 | 1 | 26 | Indel | 73.10% |
| A | 34878 | 34878 | 1 | 26 | Indel | 26.90% |
|  | 34878 | 34878 | 1 | 26 | Indel | 73.10% |
| T | 34877 | 34877 | 1 | 26 | Indel | 30.80% |
|  | 34877 | 34877 | 1 | 26 | Indel | 69.20% |
| A | 34876 | 34876 | 1 | 26 | Indel | 30.80% |
|  | 34876 | 34876 | 1 | 26 | Indel | 69.20% |
| A | 34875 | 34875 | 1 | 26 | Indel | 30.80% |
|  | 34875 | 34875 | 1 | 26 | Indel | 69.20% |
| A | 34874 | 34874 | 1 | 26 | Indel | 30.80% |
|  | 34874 | 34874 | 1 | 26 | Indel | 69.20% |
| T | 34873 | 34873 | 1 | 26 | Indel | 30.80% |
|  | 34873 | 34873 | 1 | 26 | Indel | 69.20% |
| T | 34872 | 34872 | 1 | 26 | Indel | 30.80% |
|  | 34872 | 34872 | 1 | 26 | Indel | 69.20% |
| A | 34871 | 34871 | 1 | 26 | Indel | 30.80% |
|  | 34871 | 34871 | 1 | 26 | Indel | 69.20% |
| T | 34870 | 34870 | 1 | 26 | Indel | 30.80% |
|  | 34870 | 34870 | 1 | 26 | Indel | 69.20% |
| T | 34869 | 34869 | 1 | 26 | Indel | 30.80% |
|  | 34869 | 34869 | 1 | 26 | Indel | 69.20% |
| A | 34868 | 34868 | 1 | 26 | Indel | 30.80% |
|  | 34868 | 34868 | 1 | 26 | Indel | 69.20% |
| A | 34867 | 34867 | 1 | 26 | Indel | 30.80% |
|  | 34867 | 34867 | 1 | 26 | Indel | 69.20% |
| T | 34866 | 34866 | 1 | 26 | Indel | 30.80% |
|  | 34866 | 34866 | 1 | 26 | Indel | 69.20% |
| T | 34865 | 34865 | 1 | 26 | Indel | 30.80% |
|  | 34865 | 34865 | 1 | 26 | Indel | 69.20% |
| A | 34864 | 34864 | 1 | 26 | Indel | 30.80% |
|  | 34864 | 34864 | 1 | 26 | Indel | 69.20% |
| T | 34863 | 34863 | 1 | 26 | Indel | 30.80% |
|  | 34863 | 34863 | 1 | 26 | Indel | 69.20% |
| T | 34862 | 34862 | 1 | 26 | Indel | 30.80% |
|  | 34862 | 34862 | 1 | 26 | Indel | 69.20% |
| A | 34861 | 34861 | 1 | 26 | Indel | 30.80% |
|  | 34861 | 34861 | 1 | 26 | Indel | 69.20% |
| T | 34860 | 34860 | 1 | 26 | Indel | 30.80% |
|  | 34860 | 34860 | 1 | 26 | Indel | 69.20% |
| A | 34859 | 34859 | 1 | 26 | Indel | 30.80% |
|  | 34859 | 34859 | 1 | 26 | Indel | 69.20% |
| T | 34858 | 34858 | 1 | 26 | Indel | 30.80% |
|  | 34858 | 34858 | 1 | 26 | Indel | 69.20% |
| A | 34857 | 34857 | 1 | 26 | Indel | 30.80% |
|  | 34857 | 34857 | 1 | 26 | Indel | 69.20% |
| A | 34856 | 34856 | 1 | 26 | Indel | 30.80% |
|  | 34856 | 34856 | 1 | 26 | Indel | 69.20% |
| A | 34855 | 34855 | 1 | 26 | Indel | 30.80% |
|  | 34855 | 34855 | 1 | 26 | Indel | 69.20% |
| A | 34854 | 34854 | 1 | 26 | Indel | 30.80% |
|  | 34854 | 34854 | 1 | 26 | Indel | 69.20% |
| T | 34853 | 34853 | 1 | 26 | Indel | 30.80% |
|  | 34853 | 34853 | 1 | 26 | Indel | 69.20% |
| T | 34852 | 34852 | 1 | 26 | Indel | 30.80% |
|  | 34852 | 34852 | 1 | 26 | Indel | 69.20% |
| A | 34851 | 34851 | 1 | 26 | Indel | 30.80% |
|  | 34851 | 34851 | 1 | 26 | Indel | 69.20% |
| T | 34850 | 34850 | 1 | 26 | Indel | 30.80% |
|  | 34850 | 34850 | 1 | 26 | Indel | 69.20% |
| A | 34849 | 34849 | 1 | 26 | Indel | 30.80% |
|  | 34849 | 34849 | 1 | 26 | Indel | 69.20% |
| A | 34828 | 34828 | 1 | 26 | Indel | 26.90% |
|  | 34828 | 34828 | 1 | 26 | Indel | 73.10% |
| A | 34827 | 34827 | 1 | 26 | Indel | 26.90% |
|  | 34827 | 34827 | 1 | 26 | Indel | 73.10% |
| A | 34826 | 34826 | 1 | 26 | Indel | 26.90% |
|  | 34826 | 34826 | 1 | 26 | Indel | 73.10% |
| T | 34825 | 34825 | 1 | 26 | Indel | 26.90% |
|  | 34825 | 34825 | 1 | 26 | Indel | 73.10% |
| T | 34824 | 34824 | 1 | 26 | Indel | 26.90% |
|  | 34824 | 34824 | 1 | 26 | Indel | 73.10% |
| A | 34823 | 34823 | 1 | 26 | Indel | 26.90% |
|  | 34823 | 34823 | 1 | 26 | Indel | 73.10% |
| T | 34822 | 34822 | 1 | 26 | Indel | 26.90% |
|  | 34822 | 34822 | 1 | 26 | Indel | 73.10% |
| A | 34821 | 34821 | 1 | 26 | Indel | 26.90% |
|  | 34821 | 34821 | 1 | 26 | Indel | 73.10% |
| A | 34820 | 34820 | 1 | 26 | Indel | 26.90% |
|  | 34820 | 34820 | 1 | 26 | Indel | 73.10% |
| T | 34819 | 34819 | 1 | 26 | Indel | 26.90% |
|  | 34819 | 34819 | 1 | 26 | Indel | 73.10% |
| T | 34818 | 34818 | 1 | 26 | Indel | 26.90% |
|  | 34818 | 34818 | 1 | 26 | Indel | 73.10% |
| A | 34817 | 34817 | 1 | 26 | Indel | 26.90% |
|  | 34817 | 34817 | 1 | 26 | Indel | 73.10% |
| T | 34816 | 34816 | 1 | 26 | Indel | 26.90% |
|  | 34816 | 34816 | 1 | 26 | Indel | 73.10% |
| A | 34815 | 34815 | 1 | 26 | Indel | 30.80% |
|  | 34815 | 34815 | 1 | 26 | Indel | 69.20% |
| A | 34814 | 34814 | 1 | 26 | Indel | 30.80% |
|  | 34814 | 34814 | 1 | 26 | Indel | 69.20% |
| A | 34813 | 34813 | 1 | 26 | Indel | 30.80% |
|  | 34813 | 34813 | 1 | 26 | Indel | 69.20% |
| A | 34812 | 34812 | 1 | 26 | Indel | 30.80% |
|  | 34812 | 34812 | 1 | 26 | Indel | 69.20% |
| T | 34811 | 34811 | 1 | 26 | Indel | 26.90% |
|  | 34811 | 34811 | 1 | 26 | Indel | 69.20% |
| T | 34810 | 34810 | 1 | 26 | Indel | 30.80% |
|  | 34810 | 34810 | 1 | 26 | Indel | 69.20% |
| A | 34809 | 34809 | 1 | 26 | Indel | 30.80% |
|  | 34809 | 34809 | 1 | 26 | Indel | 69.20% |
| T | 34808 | 34808 | 1 | 26 | Indel | 30.80% |
|  | 34808 | 34808 | 1 | 26 | Indel | 69.20% |
| T | 34807 | 34807 | 1 | 26 | Indel | 30.80% |
|  | 34807 | 34807 | 1 | 26 | Indel | 69.20% |
| A | 34806 | 34806 | 1 | 26 | Indel | 30.80% |
|  | 34806 | 34806 | 1 | 26 | Indel | 69.20% |
| A | 34805 | 34805 | 1 | 26 | Indel | 30.80% |
|  | 34805 | 34805 | 1 | 26 | Indel | 69.20% |
| T | 34804 | 34804 | 1 | 26 | Indel | 30.80% |
|  | 34804 | 34804 | 1 | 26 | Indel | 69.20% |
| T | 34803 | 34803 | 1 | 26 | Indel | 30.80% |
|  | 34803 | 34803 | 1 | 26 | Indel | 69.20% |
| A | 34802 | 34802 | 1 | 26 | Indel | 30.80% |
|  | 34802 | 34802 | 1 | 26 | Indel | 69.20% |
| T | 34801 | 34801 | 1 | 26 | Indel | 30.80% |
|  | 34801 | 34801 | 1 | 26 | Indel | 69.20% |
| T | 34800 | 34800 | 1 | 26 | Indel | 30.80% |
|  | 34800 | 34800 | 1 | 26 | Indel | 69.20% |
| A | 34799 | 34799 | 1 | 26 | Indel | 30.80% |
|  | 34799 | 34799 | 1 | 26 | Indel | 69.20% |
| T | 34798 | 34798 | 1 | 26 | Indel | 30.80% |
|  | 34798 | 34798 | 1 | 26 | Indel | 69.20% |
| A | 34797 | 34797 | 1 | 26 | Indel | 30.80% |
|  | 34797 | 34797 | 1 | 26 | Indel | 69.20% |
| T | 34796 | 34796 | 1 | 26 | Indel | 30.80% |
|  | 34796 | 34796 | 1 | 26 | Indel | 69.20% |
| A | 34795 | 34795 | 1 | 26 | Indel | 30.80% |
|  | 34795 | 34795 | 1 | 26 | Indel | 69.20% |
| A | 34794 | 34794 | 1 | 26 | Indel | 30.80% |
|  | 34794 | 34794 | 1 | 26 | Indel | 69.20% |
| A | 34793 | 34793 | 1 | 26 | Indel | 30.80% |
|  | 34793 | 34793 | 1 | 26 | Indel | 69.20% |
| A | 34792 | 34792 | 1 | 26 | Indel | 30.80% |
|  | 34792 | 34792 | 1 | 26 | Indel | 69.20% |
| A | 34791 | 34791 | 1 | 26 | Indel | 30.80% |
|  | 34791 | 34791 | 1 | 26 | Indel | 69.20% |
| A | 34790 | 34790 | 1 | 26 | Indel | 30.80% |
|  | 34790 | 34790 | 1 | 26 | Indel | 69.20% |
| T | 34789 | 34789 | 1 | 26 | Indel | 30.80% |
|  | 34789 | 34789 | 1 | 26 | Indel | 69.20% |
| A | 34788 | 34788 | 1 | 26 | Indel | 30.80% |
|  | 34788 | 34788 | 1 | 26 | Indel | 69.20% |
| T | 34787 | 34787 | 1 | 26 | Indel | 30.80% |
|  | 34787 | 34787 | 1 | 26 | Indel | 69.20% |
| T | 34786 | 34786 | 1 | 26 | Indel | 30.80% |
|  | 34786 | 34786 | 1 | 26 | Indel | 69.20% |
| A | 34785 | 34785 | 1 | 26 | Indel | 30.80% |
|  | 34785 | 34785 | 1 | 26 | Indel | 69.20% |
| A | 34784 | 34784 | 1 | 26 | Indel | 30.80% |
|  | 34784 | 34784 | 1 | 26 | Indel | 69.20% |
| T | 34783 | 34783 | 1 | 26 | Indel | 30.80% |
|  | 34783 | 34783 | 1 | 26 | Indel | 69.20% |
| T | 34782 | 34782 | 1 | 26 | Indel | 30.80% |
|  | 34782 | 34782 | 1 | 26 | Indel | 69.20% |
| A | 34781 | 34781 | 1 | 26 | Indel | 30.80% |
|  | 34781 | 34781 | 1 | 26 | Indel | 69.20% |
| T | 34780 | 34780 | 1 | 26 | Indel | 30.80% |
|  | 34780 | 34780 | 1 | 26 | Indel | 69.20% |
| T | 34779 | 34779 | 1 | 26 | Indel | 30.80% |
|  | 34779 | 34779 | 1 | 26 | Indel | 69.20% |
| A | 34778 | 34778 | 1 | 26 | Indel | 30.80% |
|  | 34778 | 34778 | 1 | 26 | Indel | 69.20% |
| T | 34777 | 34777 | 1 | 26 | Indel | 30.80% |
|  | 34777 | 34777 | 1 | 26 | Indel | 69.20% |
| A | 34776 | 34776 | 1 | 26 | Indel | 30.80% |
|  | 34776 | 34776 | 1 | 26 | Indel | 69.20% |
| T | 34775 | 34775 | 1 | 26 | Indel | 30.80% |
|  | 34775 | 34775 | 1 | 26 | Indel | 69.20% |
| A | 34774 | 34774 | 1 | 26 | Indel | 26.90% |
|  | 34774 | 34774 | 1 | 26 | Indel | 73.10% |
| A | 34773 | 34773 | 1 | 26 | Indel | 26.90% |
|  | 34773 | 34773 | 1 | 26 | Indel | 73.10% |
| A | 34772 | 34772 | 1 | 26 | Indel | 26.90% |
|  | 34772 | 34772 | 1 | 26 | Indel | 73.10% |
| A | 34771 | 34771 | 1 | 26 | Indel | 26.90% |
|  | 34771 | 34771 | 1 | 26 | Indel | 73.10% |
| A | 34770 | 34770 | 1 | 26 | Indel | 26.90% |
|  | 34770 | 34770 | 1 | 26 | Indel | 73.10% |
| T | 34769 | 34769 | 1 | 26 | Indel | 26.90% |
|  | 34769 | 34769 | 1 | 26 | Indel | 73.10% |
| A | 34768 | 34768 | 1 | 26 | Indel | 26.90% |
|  | 34768 | 34768 | 1 | 26 | Indel | 73.10% |
| T | 34767 | 34767 | 1 | 26 | Indel | 26.90% |
|  | 34767 | 34767 | 1 | 26 | Indel | 73.10% |
| A | 34766 | 34766 | 1 | 26 | Indel | 30.80% |
|  | 34766 | 34766 | 1 | 26 | Indel | 69.20% |
| A | 34765 | 34765 | 1 | 26 | Indel | 30.80% |
|  | 34765 | 34765 | 1 | 26 | Indel | 69.20% |
| A | 34764 | 34764 | 1 | 26 | Indel | 30.80% |
|  | 34764 | 34764 | 1 | 26 | Indel | 69.20% |
| A | 34763 | 34763 | 1 | 26 | Indel | 30.80% |
|  | 34763 | 34763 | 1 | 26 | Indel | 69.20% |
| A | 34762 | 34762 | 1 | 26 | Indel | 30.80% |
|  | 34762 | 34762 | 1 | 26 | Indel | 69.20% |
| A | 34761 | 34761 | 1 | 26 | Indel | 30.80% |
|  | 34761 | 34761 | 1 | 26 | Indel | 69.20% |
| T | 34760 | 34760 | 1 | 26 | Indel | 30.80% |
|  | 34760 | 34760 | 1 | 26 | Indel | 69.20% |
| A | 34759 | 34759 | 1 | 26 | Indel | 30.80% |
|  | 34759 | 34759 | 1 | 26 | Indel | 69.20% |
| A | 34758 | 34758 | 1 | 26 | Indel | 30.80% |
|  | 34758 | 34758 | 1 | 26 | Indel | 69.20% |
| A | 34757 | 34757 | 1 | 26 | Indel | 30.80% |
|  | 34757 | 34757 | 1 | 26 | Indel | 69.20% |
| A | 34756 | 34756 | 1 | 26 | Indel | 30.80% |
|  | 34756 | 34756 | 1 | 26 | Indel | 69.20% |
| T | 34755 | 34755 | 1 | 26 | Indel | 30.80% |
|  | 34755 | 34755 | 1 | 26 | Indel | 69.20% |
| A | 34754 | 34754 | 1 | 26 | Indel | 30.80% |
|  | 34754 | 34754 | 1 | 26 | Indel | 69.20% |
| A | 34753 | 34753 | 1 | 26 | Indel | 30.80% |
|  | 34753 | 34753 | 1 | 26 | Indel | 69.20% |
| A | 34752 | 34752 | 1 | 26 | Indel | 30.80% |
|  | 34752 | 34752 | 1 | 26 | Indel | 69.20% |
| T | 34751 | 34751 | 1 | 26 | Indel | 30.80% |
|  | 34751 | 34751 | 1 | 26 | Indel | 69.20% |
| T | 34750 | 34750 | 1 | 26 | Indel | 30.80% |
|  | 34750 | 34750 | 1 | 26 | Indel | 69.20% |
| A | 34749 | 34749 | 1 | 26 | Indel | 30.80% |
|  | 34749 | 34749 | 1 | 26 | Indel | 69.20% |
| T | 34748 | 34748 | 1 | 26 | Indel | 26.90% |
|  | 34748 | 34748 | 1 | 26 | Indel | 73.10% |
| T | 34747 | 34747 | 1 | 26 | Indel | 26.90% |
|  | 34747 | 34747 | 1 | 26 | Indel | 73.10% |
| A | 34746 | 34746 | 1 | 26 | Indel | 26.90% |
|  | 34746 | 34746 | 1 | 26 | Indel | 73.10% |
| T | 34745 | 34745 | 1 | 26 | Indel | 26.90% |
|  | 34745 | 34745 | 1 | 26 | Indel | 73.10% |
| A | 34744 | 34744 | 1 | 26 | Indel | 26.90% |
|  | 34744 | 34744 | 1 | 26 | Indel | 73.10% |
| T | 34743 | 34743 | 1 | 26 | Indel | 26.90% |
|  | 34743 | 34743 | 1 | 26 | Indel | 73.10% |
| T | 34742 | 34742 | 1 | 26 | Indel | 26.90% |
|  | 34742 | 34742 | 1 | 26 | Indel | 73.10% |
| A | 34741 | 34741 | 1 | 26 | Indel | 26.90% |
|  | 34741 | 34741 | 1 | 26 | Indel | 73.10% |
| A | 34740 | 34740 | 1 | 26 | Indel | 26.90% |
|  | 34740 | 34740 | 1 | 26 | Indel | 73.10% |
| A | 34739 | 34739 | 1 | 26 | Indel | 26.90% |
|  | 34739 | 34739 | 1 | 26 | Indel | 73.10% |
| T | 34738 | 34738 | 1 | 26 | Indel | 26.90% |
|  | 34738 | 34738 | 1 | 26 | Indel | 73.10% |
| T | 34737 | 34737 | 1 | 26 | Indel | 26.90% |
|  | 34737 | 34737 | 1 | 26 | Indel | 73.10% |
| A | 34736 | 34736 | 1 | 26 | Indel | 26.90% |
|  | 34736 | 34736 | 1 | 26 | Indel | 73.10% |
| T | 34735 | 34735 | 1 | 26 | Indel | 30.80% |
|  | 34735 | 34735 | 1 | 26 | Indel | 69.20% |
| T | 34734 | 34734 | 1 | 26 | Indel | 30.80% |
|  | 34734 | 34734 | 1 | 26 | Indel | 69.20% |
| A | 34733 | 34733 | 1 | 26 | Indel | 30.80% |
|  | 34733 | 34733 | 1 | 26 | Indel | 69.20% |
| T | 34732 | 34732 | 1 | 26 | Indel | 30.80% |
|  | 34732 | 34732 | 1 | 26 | Indel | 69.20% |
| A | 34731 | 34731 | 1 | 26 | Indel | 30.80% |
|  | 34731 | 34731 | 1 | 26 | Indel | 69.20% |
| T | 34730 | 34730 | 1 | 26 | Indel | 30.80% |
|  | 34730 | 34730 | 1 | 26 | Indel | 69.20% |
| T | 34729 | 34729 | 1 | 26 | Indel | 30.80% |
|  | 34729 | 34729 | 1 | 26 | Indel | 69.20% |
| T | 34728 | 34728 | 1 | 26 | Indel | 34.60% |
|  | 34728 | 34728 | 1 | 26 | Indel | 65.40% |
| A | 34727 | 34727 | 1 | 26 | Indel | 34.60% |
|  | 34727 | 34727 | 1 | 26 | Indel | 65.40% |
| T | 34726 | 34726 | 1 | 26 | Indel | 34.60% |
|  | 34726 | 34726 | 1 | 26 | Indel | 65.40% |
| A | 34725 | 34725 | 1 | 26 | Indel | 34.60% |
|  | 34725 | 34725 | 1 | 26 | Indel | 65.40% |
| T | 34724 | 34724 | 1 | 26 | Indel | 34.60% |
|  | 34724 | 34724 | 1 | 26 | Indel | 65.40% |
| A | 34723 | 34723 | 1 | 26 | Indel | 34.60% |
|  | 34723 | 34723 | 1 | 26 | Indel | 65.40% |
| A | 34582 | 34582 | 1 | 26 | Indel | 69.20% |
|  | 34582 | 34582 | 1 | 26 | Indel | 30.80% |
| A | 34581 | 34581 | 1 | 26 | Indel | 69.20% |
|  | 34581 | 34581 | 1 | 26 | Indel | 30.80% |
| A | 34580 | 34580 | 1 | 26 | Indel | 69.20% |
|  | 34580 | 34580 | 1 | 26 | Indel | 30.80% |
| A | 34579 | 34579 | 1 | 26 | Indel | 69.20% |
|  | 34579 | 34579 | 1 | 26 | Indel | 30.80% |
| A | 34578 | 34578 | 1 | 26 | Indel | 69.20% |
|  | 34578 | 34578 | 1 | 26 | Indel | 30.80% |
| A | 34577 | 34577 | 1 | 26 | Indel | 69.20% |
|  | 34577 | 34577 | 1 | 26 | Indel | 30.80% |
| A | 34576 | 34576 | 1 | 26 | Indel | 69.20% |
|  | 34576 | 34576 | 1 | 26 | Indel | 30.80% |
| T | 34575 | 34575 | 1 | 26 | Indel | 69.20% |
|  | 34575 | 34575 | 1 | 26 | Indel | 30.80% |
| A | 34574 | 34574 | 1 | 26 | Indel | 69.20% |
|  | 34574 | 34574 | 1 | 26 | Indel | 30.80% |
| A | 34573 | 34573 | 1 | 26 | Indel | 69.20% |
|  | 34573 | 34573 | 1 | 26 | Indel | 30.80% |
| T | 34572 | 34572 | 1 | 26 | Indel | 69.20% |
|  | 34572 | 34572 | 1 | 26 | Indel | 30.80% |
| G | 34571 | 34571 | 1 | 26 | Indel | 69.20% |
|  | 34571 | 34571 | 1 | 26 | Indel | 30.80% |
| A | 34570 | 34570 | 1 | 26 | Indel | 69.20% |
|  | 34570 | 34570 | 1 | 26 | Indel | 30.80% |
| A | 34569 | 34569 | 1 | 26 | Indel | 69.20% |
|  | 34569 | 34569 | 1 | 26 | Indel | 30.80% |
| A | 34568 | 34568 | 1 | 26 | Indel | 69.20% |
|  | 34568 | 34568 | 1 | 26 | Indel | 30.80% |
| T | 34567 | 34567 | 1 | 26 | Indel | 69.20% |
|  | 34567 | 34567 | 1 | 26 | Indel | 30.80% |
| A | 34566 | 34566 | 1 | 26 | Indel | 69.20% |
|  | 34566 | 34566 | 1 | 26 | Indel | 30.80% |
| A | 34565 | 34565 | 1 | 26 | Indel | 69.20% |
|  | 34565 | 34565 | 1 | 26 | Indel | 30.80% |
| T | 34564 | 34564 | 1 | 26 | Indel | 69.20% |
|  | 34564 | 34564 | 1 | 26 | Indel | 30.80% |
| T | 33539 | 33539 | 1 | 26 | Indel | 69.20% |
|  | 33539 | 33539 | 1 | 26 | Indel | 30.80% |
| G | 33538 | 33538 | 1 | 26 | Indel | 69.20% |
|  | 33538 | 33538 | 1 | 26 | Indel | 30.80% |
| G | 33537 | 33537 | 1 | 26 | Indel | 69.20% |
|  | 33537 | 33537 | 1 | 26 | Indel | 30.80% |
| T | 33536 | 33536 | 1 | 26 | Indel | 69.20% |
|  | 33536 | 33536 | 1 | 26 | Indel | 30.80% |
| G | 33535 | 33535 | 1 | 26 | Indel | 69.20% |
|  | 33535 | 33535 | 1 | 26 | Indel | 30.80% |
| A | 33534 | 33534 | 1 | 26 | Indel | 69.20% |
|  | 33534 | 33534 | 1 | 26 | Indel | 30.80% |
| T | 33533 | 33533 | 1 | 26 | Indel | 69.20% |
|  | 33533 | 33533 | 1 | 26 | Indel | 30.80% |
| A | 33532 | 33532 | 1 | 26 | Indel | 69.20% |
|  | 33532 | 33532 | 1 | 26 | Indel | 30.80% |
| G | 33421 | 33421 | 1 | 26 | Indel | 69.20% |
|  | 33421 | 33421 | 1 | 26 | Indel | 26.90% |
| A | 32507 | 32507 | 1 | 26 | Indel | 30.80% |
|  | 32507 | 32507 | 1 | 26 | Indel | 69.20% |
| A | 32506 | 32506 | 1 | 26 | Indel | 34.60% |
|  | 32506 | 32506 | 1 | 26 | Indel | 65.40% |
| A | 32505 | 32505 | 1 | 26 | Indel | 34.60% |
|  | 32505 | 32505 | 1 | 26 | Indel | 65.40% |
| T | 32338 | 32338 | 1 | 26 | Indel | 69.20% |
|  | 32338 | 32338 | 1 | 26 | Indel | 30.80% |
| A | 30757 | 30757 | 1 | 26 | Indel | 69.20% |
|  | 30757 | 30757 | 1 | 26 | Indel | 30.80% |
| G | 30756 | 30756 | 1 | 26 | Indel | 69.20% |
|  | 30756 | 30756 | 1 | 26 | Indel | 30.80% |
| T | 30755 | 30755 | 1 | 26 | Indel | 69.20% |
|  | 30755 | 30755 | 1 | 26 | Indel | 30.80% |
| A | 30754 | 30754 | 1 | 26 | Indel | 69.20% |
|  | 30754 | 30754 | 1 | 26 | Indel | 30.80% |
| T | 30753 | 30753 | 1 | 26 | Indel | 69.20% |
|  | 30753 | 30753 | 1 | 26 | Indel | 30.80% |
| A | 30752 | 30752 | 1 | 26 | Indel | 69.20% |
|  | 30752 | 30752 | 1 | 26 | Indel | 30.80% |
| T | 30751 | 30751 | 1 | 26 | Indel | 69.20% |
|  | 30751 | 30751 | 1 | 26 | Indel | 30.80% |
| A | 30750 | 30750 | 1 | 26 | Indel | 69.20% |
|  | 30750 | 30750 | 1 | 26 | Indel | 30.80% |
| A | 29250 | 29250 | 1 | 26 | Indel | 30.80% |
|  | 29250 | 29250 | 1 | 26 | Indel | 69.20% |
| A | 29249 | 29249 | 1 | 26 | Indel | 30.80% |
|  | 29249 | 29249 | 1 | 26 | Indel | 69.20% |
| G | 29248 | 29248 | 1 | 26 | Indel | 30.80% |
|  | 29248 | 29248 | 1 | 26 | Indel | 69.20% |
| T | 29247 | 29247 | 1 | 26 | Indel | 30.80% |
|  | 29247 | 29247 | 1 | 26 | Indel | 69.20% |
| T | 29246 | 29246 | 1 | 26 | Indel | 30.80% |
|  | 29246 | 29246 | 1 | 26 | Indel | 69.20% |
| A | 29245 | 29245 | 1 | 26 | Indel | 30.80% |
|  | 29245 | 29245 | 1 | 26 | Indel | 69.20% |
| A | 29244 | 29244 | 1 | 26 | Indel | 30.80% |
|  | 29244 | 29244 | 1 | 26 | Indel | 69.20% |
| C | 29243 | 29243 | 1 | 26 | Indel | 30.80% |
|  | 29243 | 29243 | 1 | 26 | Indel | 69.20% |
| T | 29242 | 29242 | 1 | 26 | Indel | 30.80% |
|  | 29242 | 29242 | 1 | 26 | Indel | 69.20% |
| A | 29241 | 29241 | 1 | 26 | Indel | 30.80% |
|  | 29241 | 29241 | 1 | 26 | Indel | 69.20% |
| A | 29240 | 29240 | 1 | 26 | Indel | 30.80% |
|  | 29240 | 29240 | 1 | 26 | Indel | 69.20% |
| G | 29239 | 29239 | 1 | 26 | Indel | 30.80% |
|  | 29239 | 29239 | 1 | 26 | Indel | 69.20% |
| A | 29238 | 29238 | 1 | 26 | Indel | 30.80% |
|  | 29238 | 29238 | 1 | 26 | Indel | 69.20% |
| T | 29237 | 29237 | 1 | 26 | Indel | 30.80% |
|  | 29237 | 29237 | 1 | 26 | Indel | 69.20% |
| A | 29236 | 29236 | 1 | 26 | Indel | 30.80% |
|  | 29236 | 29236 | 1 | 26 | Indel | 69.20% |
| A | 29235 | 29235 | 1 | 26 | Indel | 30.80% |
|  | 29235 | 29235 | 1 | 26 | Indel | 69.20% |
| T | 29234 | 29234 | 1 | 26 | Indel | 30.80% |
|  | 29234 | 29234 | 1 | 26 | Indel | 69.20% |
| T | 29233 | 29233 | 1 | 26 | Indel | 30.80% |
|  | 29233 | 29233 | 1 | 26 | Indel | 69.20% |
| T | 29232 | 29232 | 1 | 26 | Indel | 30.80% |
|  | 29232 | 29232 | 1 | 26 | Indel | 69.20% |
| A | 29073 | 29073 | 1 | 26 | Indel | 69.20% |
|  | 29073 | 29073 | 1 | 26 | Indel | 30.80% |
| T | 28790 | 28790 | 1 | 26 | Indel | 30.80% |
|  | 28790 | 28790 | 1 | 26 | Indel | 69.20% |
| C | 28789 | 28789 | 1 | 26 | Indel | 30.80% |
|  | 28789 | 28789 | 1 | 26 | Indel | 69.20% |
| G | 28788 | 28788 | 1 | 26 | Indel | 30.80% |
|  | 28788 | 28788 | 1 | 26 | Indel | 69.20% |
| A | 28787 | 28787 | 1 | 26 | Indel | 30.80% |
|  | 28787 | 28787 | 1 | 26 | Indel | 69.20% |
| T | 28786 | 28786 | 1 | 26 | Indel | 30.80% |
|  | 28786 | 28786 | 1 | 26 | Indel | 69.20% |
| A | 28785 | 28785 | 1 | 26 | Indel | 30.80% |
|  | 28785 | 28785 | 1 | 26 | Indel | 69.20% |
| T | 28784 | 28784 | 1 | 26 | Indel | 30.80% |
|  | 28784 | 28784 | 1 | 26 | Indel | 69.20% |
| C | 28783 | 28783 | 1 | 26 | Indel | 30.80% |
|  | 28783 | 28783 | 1 | 26 | Indel | 69.20% |
| A | 28782 | 28782 | 1 | 26 | Indel | 30.80% |
|  | 28782 | 28782 | 1 | 26 | Indel | 69.20% |
| A | 28781 | 28781 | 1 | 26 | Indel | 30.80% |
|  | 28781 | 28781 | 1 | 26 | Indel | 69.20% |
| T | 28780 | 28780 | 1 | 26 | Indel | 30.80% |
|  | 28780 | 28780 | 1 | 26 | Indel | 69.20% |
| A | 28779 | 28779 | 1 | 26 | Indel | 30.80% |
|  | 28779 | 28779 | 1 | 26 | Indel | 69.20% |
| T | 28778 | 28778 | 1 | 26 | Indel | 30.80% |
|  | 28778 | 28778 | 1 | 26 | Indel | 69.20% |
| A | 28777 | 28777 | 1 | 26 | Indel | 30.80% |
|  | 28777 | 28777 | 1 | 26 | Indel | 69.20% |
| A | 28776 | 28776 | 1 | 26 | Indel | 30.80% |
|  | 28776 | 28776 | 1 | 26 | Indel | 69.20% |
| A | 28775 | 28775 | 1 | 26 | Indel | 30.80% |
|  | 28775 | 28775 | 1 | 26 | Indel | 69.20% |
| T | 28774 | 28774 | 1 | 26 | Indel | 30.80% |
|  | 28774 | 28774 | 1 | 26 | Indel | 69.20% |
| C | 28773 | 28773 | 1 | 26 | Indel | 30.80% |
|  | 28773 | 28773 | 1 | 26 | Indel | 69.20% |
| T | 28772 | 28772 | 1 | 26 | Indel | 30.80% |
|  | 28772 | 28772 | 1 | 26 | Indel | 69.20% |
| A | 28771 | 28771 | 1 | 26 | Indel | 30.80% |
|  | 28771 | 28771 | 1 | 26 | Indel | 69.20% |
| T | 28199 | 28199 | 1 | 26 | Indel | 30.80% |
|  | 28199 | 28199 | 1 | 26 | Indel | 69.20% |
| A | 17622 | 17622 | 1 | 26 | Indel | 30.80% |
|  | 17622 | 17622 | 1 | 26 | Indel | 69.20% |
| T | 17621 | 17621 | 1 | 26 | Indel | 30.80% |
|  | 17621 | 17621 | 1 | 26 | Indel | 69.20% |
| C | 15291 | 15291 | 1 | 26 | Indel | 73.10% |
|  | 15291 | 15291 | 1 | 26 | Indel | 26.90% |
| C | 15290 | 15290 | 1 | 26 | Indel | 69.20% |
|  | 15290 | 15290 | 1 | 26 | Indel | 30.80% |
| C | 15289 | 15289 | 1 | 26 | Indel | 69.20% |
|  | 15289 | 15289 | 1 | 26 | Indel | 30.80% |
| A | 14754 | 14754 | 1 | 26 | Indel | 69.20% |
|  | 14754 | 14754 | 1 | 26 | Indel | 30.80% |
| T | 13783 | 13783 | 1 | 26 | Indel | 30.80% |
|  | 13783 | 13783 | 1 | 26 | Indel | 69.20% |
| A | 13290 | 13290 | 1 | 26 | Indel | 30.80% |
|  | 13290 | 13290 | 1 | 26 | Indel | 69.20% |
| A | 13289 | 13289 | 1 | 26 | Indel | 30.80% |
|  | 13289 | 13289 | 1 | 26 | Indel | 69.20% |
| T | 13288 | 13288 | 1 | 26 | Indel | 30.80% |
|  | 13288 | 13288 | 1 | 26 | Indel | 69.20% |
| T | 13287 | 13287 | 1 | 26 | Indel | 30.80% |
|  | 13287 | 13287 | 1 | 26 | Indel | 69.20% |
| T | 13286 | 13286 | 1 | 26 | Indel | 30.80% |
|  | 13286 | 13286 | 1 | 26 | Indel | 69.20% |
| T | 12703 | 12703 | 1 | 26 | Indel | 73.10% |
|  | 12703 | 12703 | 1 | 26 | Indel | 26.90% |
| A | 10925 | 10925 | 1 | 26 | Indel | 69.20% |
|  | 10925 | 10925 | 1 | 26 | Indel | 30.80% |
| T | 10924 | 10924 | 1 | 26 | Indel | 69.20% |
|  | 10924 | 10924 | 1 | 26 | Indel | 30.80% |
| T | 10923 | 10923 | 1 | 26 | Indel | 69.20% |
|  | 10923 | 10923 | 1 | 26 | Indel | 30.80% |
| T | 10922 | 10922 | 1 | 26 | Indel | 69.20% |
|  | 10922 | 10922 | 1 | 26 | Indel | 30.80% |
| T | 10921 | 10921 | 1 | 26 | Indel | 69.20% |
|  | 10921 | 10921 | 1 | 26 | Indel | 30.80% |
| T | 10867 | 10867 | 1 | 26 | Indel | 69.20% |
|  | 10867 | 10867 | 1 | 26 | Indel | 30.80% |
| A | 10866 | 10866 | 1 | 26 | Indel | 69.20% |
|  | 10866 | 10866 | 1 | 26 | Indel | 30.80% |
| A | 10865 | 10865 | 1 | 26 | Indel | 69.20% |
|  | 10865 | 10865 | 1 | 26 | Indel | 30.80% |
| T | 10864 | 10864 | 1 | 26 | Indel | 69.20% |
|  | 10864 | 10864 | 1 | 26 | Indel | 30.80% |
| T | 10863 | 10863 | 1 | 26 | Indel | 69.20% |
|  | 10863 | 10863 | 1 | 26 | Indel | 30.80% |
| T | 10862 | 10862 | 1 | 26 | Indel | 69.20% |
|  | 10862 | 10862 | 1 | 26 | Indel | 30.80% |
| T | 10861 | 10861 | 1 | 26 | Indel | 69.20% |
|  | 10861 | 10861 | 1 | 26 | Indel | 30.80% |
| T | 10860 | 10860 | 1 | 26 | Indel | 69.20% |
|  | 10860 | 10860 | 1 | 26 | Indel | 30.80% |
| A | 10859 | 10859 | 1 | 26 | Indel | 69.20% |
|  | 10859 | 10859 | 1 | 26 | Indel | 30.80% |
| T | 10858 | 10858 | 1 | 26 | Indel | 69.20% |
|  | 10858 | 10858 | 1 | 26 | Indel | 30.80% |
| T | 10857 | 10857 | 1 | 26 | Indel | 69.20% |
|  | 10857 | 10857 | 1 | 26 | Indel | 30.80% |
| G | 10856 | 10856 | 1 | 26 | Indel | 69.20% |
|  | 10856 | 10856 | 1 | 26 | Indel | 30.80% |
| A | 10855 | 10855 | 1 | 26 | Indel | 69.20% |
|  | 10855 | 10855 | 1 | 26 | Indel | 30.80% |
| A | 10854 | 10854 | 1 | 26 | Indel | 69.20% |
|  | 10854 | 10854 | 1 | 26 | Indel | 30.80% |
| T | 10853 | 10853 | 1 | 26 | Indel | 69.20% |
|  | 10853 | 10853 | 1 | 26 | Indel | 30.80% |
| A | 10852 | 10852 | 1 | 26 | Indel | 69.20% |
|  | 10852 | 10852 | 1 | 26 | Indel | 30.80% |
| A | 10851 | 10851 | 1 | 26 | Indel | 69.20% |
|  | 10851 | 10851 | 1 | 26 | Indel | 30.80% |
| T | 10850 | 10850 | 1 | 26 | Indel | 69.20% |
|  | 10850 | 10850 | 1 | 26 | Indel | 30.80% |
| T | 10849 | 10849 | 1 | 26 | Indel | 69.20% |
|  | 10849 | 10849 | 1 | 26 | Indel | 30.80% |
| A | 10848 | 10848 | 1 | 26 | Indel | 69.20% |
|  | 10848 | 10848 | 1 | 26 | Indel | 30.80% |
| A | 10847 | 10847 | 1 | 26 | Indel | 69.20% |
|  | 10847 | 10847 | 1 | 26 | Indel | 30.80% |
| T | 10846 | 10846 | 1 | 26 | Indel | 69.20% |
|  | 10846 | 10846 | 1 | 26 | Indel | 30.80% |
| T | 10845 | 10845 | 1 | 26 | Indel | 69.20% |
|  | 10845 | 10845 | 1 | 26 | Indel | 30.80% |
| A | 10844 | 10844 | 1 | 26 | Indel | 69.20% |
|  | 10844 | 10844 | 1 | 26 | Indel | 30.80% |
| T | 10843 | 10843 | 1 | 26 | Indel | 69.20% |
|  | 10843 | 10843 | 1 | 26 | Indel | 30.80% |
| A | 10842 | 10842 | 1 | 26 | Indel | 69.20% |
|  | 10842 | 10842 | 1 | 26 | Indel | 30.80% |
| T | 10841 | 10841 | 1 | 26 | Indel | 69.20% |
|  | 10841 | 10841 | 1 | 26 | Indel | 30.80% |
| A | 10768 | 10768 | 1 | 26 | Indel | 69.20% |
|  | 10768 | 10768 | 1 | 26 | Indel | 30.80% |
| A | 10767 | 10767 | 1 | 26 | Indel | 69.20% |
|  | 10767 | 10767 | 1 | 26 | Indel | 30.80% |
| T | 10766 | 10766 | 1 | 26 | Indel | 69.20% |
|  | 10766 | 10766 | 1 | 26 | Indel | 30.80% |
| A | 10765 | 10765 | 1 | 26 | Indel | 69.20% |
|  | 10765 | 10765 | 1 | 26 | Indel | 30.80% |
| A | 10764 | 10764 | 1 | 26 | Indel | 69.20% |
|  | 10764 | 10764 | 1 | 26 | Indel | 30.80% |
| T | 10763 | 10763 | 1 | 26 | Indel | 69.20% |
|  | 10763 | 10763 | 1 | 26 | Indel | 30.80% |
| T | 10762 | 10762 | 1 | 26 | Indel | 69.20% |
|  | 10762 | 10762 | 1 | 26 | Indel | 30.80% |
| A | 10761 | 10761 | 1 | 26 | Indel | 69.20% |
|  | 10761 | 10761 | 1 | 26 | Indel | 30.80% |
| A | 10760 | 10760 | 1 | 26 | Indel | 69.20% |
|  | 10760 | 10760 | 1 | 26 | Indel | 30.80% |
| A | 10759 | 10759 | 1 | 26 | Indel | 69.20% |
|  | 10759 | 10759 | 1 | 26 | Indel | 30.80% |
| T | 10758 | 10758 | 1 | 26 | Indel | 69.20% |
|  | 10758 | 10758 | 1 | 26 | Indel | 30.80% |
| C | 10757 | 10757 | 1 | 26 | Indel | 69.20% |
|  | 10757 | 10757 | 1 | 26 | Indel | 30.80% |
| T | 10756 | 10756 | 1 | 26 | Indel | 69.20% |
|  | 10756 | 10756 | 1 | 26 | Indel | 30.80% |
| T | 10755 | 10755 | 1 | 26 | Indel | 69.20% |
|  | 10755 | 10755 | 1 | 26 | Indel | 30.80% |
| A | 10754 | 10754 | 1 | 26 | Indel | 69.20% |
|  | 10754 | 10754 | 1 | 26 | Indel | 30.80% |
| T | 10753 | 10753 | 1 | 26 | Indel | 69.20% |
|  | 10753 | 10753 | 1 | 26 | Indel | 30.80% |
| A | 10752 | 10752 | 1 | 26 | Indel | 69.20% |
|  | 10752 | 10752 | 1 | 26 | Indel | 30.80% |
| G | 10751 | 10751 | 1 | 26 | Indel | 69.20% |
|  | 10751 | 10751 | 1 | 26 | Indel | 30.80% |
| A | 10750 | 10750 | 1 | 26 | Indel | 69.20% |
|  | 10750 | 10750 | 1 | 26 | Indel | 30.80% |
| A | 10749 | 10749 | 1 | 26 | Indel | 69.20% |
|  | 10749 | 10749 | 1 | 26 | Indel | 30.80% |
| A | 10748 | 10748 | 1 | 26 | Indel | 69.20% |
|  | 10748 | 10748 | 1 | 26 | Indel | 30.80% |
| T | 10747 | 10747 | 1 | 26 | Indel | 69.20% |
|  | 10747 | 10747 | 1 | 26 | Indel | 30.80% |
| A | 10746 | 10746 | 1 | 26 | Indel | 69.20% |
|  | 10746 | 10746 | 1 | 26 | Indel | 30.80% |
| T | 10745 | 10745 | 1 | 26 | Indel | 69.20% |
|  | 10745 | 10745 | 1 | 26 | Indel | 30.80% |
| A | 10744 | 10744 | 1 | 26 | Indel | 69.20% |
|  | 10744 | 10744 | 1 | 26 | Indel | 30.80% |
| T | 10743 | 10743 | 1 | 26 | Indel | 69.20% |
|  | 10743 | 10743 | 1 | 26 | Indel | 30.80% |
| T | 10742 | 10742 | 1 | 26 | Indel | 69.20% |
|  | 10742 | 10742 | 1 | 26 | Indel | 30.80% |
| T | 10741 | 10741 | 1 | 26 | Indel | 69.20% |
|  | 10741 | 10741 | 1 | 26 | Indel | 30.80% |
| A | 10740 | 10740 | 1 | 26 | Indel | 69.20% |
|  | 10740 | 10740 | 1 | 26 | Indel | 30.80% |
| T | 10739 | 10739 | 1 | 26 | Indel | 69.20% |
|  | 10739 | 10739 | 1 | 26 | Indel | 30.80% |
| T | 10738 | 10738 | 1 | 26 | Indel | 69.20% |
|  | 10738 | 10738 | 1 | 26 | Indel | 30.80% |
| A | 10737 | 10737 | 1 | 26 | Indel | 69.20% |
|  | 10737 | 10737 | 1 | 26 | Indel | 30.80% |
| T | 10736 | 10736 | 1 | 26 | Indel | 69.20% |
|  | 10736 | 10736 | 1 | 26 | Indel | 30.80% |
| T | 10735 | 10735 | 1 | 26 | Indel | 69.20% |
|  | 10735 | 10735 | 1 | 26 | Indel | 30.80% |
| G | 10734 | 10734 | 1 | 26 | Indel | 69.20% |
|  | 10734 | 10734 | 1 | 26 | Indel | 30.80% |
| A | 10733 | 10733 | 1 | 26 | Indel | 69.20% |
|  | 10733 | 10733 | 1 | 26 | Indel | 30.80% |
| A | 10732 | 10732 | 1 | 26 | Indel | 69.20% |
|  | 10732 | 10732 | 1 | 26 | Indel | 30.80% |
| T | 10731 | 10731 | 1 | 26 | Indel | 69.20% |
|  | 10731 | 10731 | 1 | 26 | Indel | 30.80% |
| A | 10730 | 10730 | 1 | 26 | Indel | 69.20% |
|  | 10730 | 10730 | 1 | 26 | Indel | 30.80% |
| A | 10729 | 10729 | 1 | 26 | Indel | 69.20% |
|  | 10729 | 10729 | 1 | 26 | Indel | 30.80% |
| T | 10728 | 10728 | 1 | 26 | Indel | 69.20% |
|  | 10728 | 10728 | 1 | 26 | Indel | 30.80% |
| T | 10727 | 10727 | 1 | 26 | Indel | 69.20% |
|  | 10727 | 10727 | 1 | 26 | Indel | 30.80% |
| A | 10726 | 10726 | 1 | 26 | Indel | 69.20% |
|  | 10726 | 10726 | 1 | 26 | Indel | 30.80% |
| A | 10725 | 10725 | 1 | 26 | Indel | 69.20% |
|  | 10725 | 10725 | 1 | 26 | Indel | 30.80% |
| A | 10724 | 10724 | 1 | 26 | Indel | 69.20% |
|  | 10724 | 10724 | 1 | 26 | Indel | 30.80% |
| T | 10723 | 10723 | 1 | 26 | Indel | 69.20% |
|  | 10723 | 10723 | 1 | 26 | Indel | 30.80% |
| T | 10722 | 10722 | 1 | 26 | Indel | 69.20% |
|  | 10722 | 10722 | 1 | 26 | Indel | 30.80% |
| A | 10721 | 10721 | 1 | 26 | Indel | 69.20% |
|  | 10721 | 10721 | 1 | 26 | Indel | 30.80% |
| A | 10720 | 10720 | 1 | 26 | Indel | 69.20% |
|  | 10720 | 10720 | 1 | 26 | Indel | 30.80% |
| A | 10719 | 10719 | 1 | 26 | Indel | 69.20% |
|  | 10719 | 10719 | 1 | 26 | Indel | 30.80% |
| T | 10718 | 10718 | 1 | 26 | Indel | 69.20% |
|  | 10718 | 10718 | 1 | 26 | Indel | 30.80% |
| C | 10717 | 10717 | 1 | 26 | Indel | 69.20% |
|  | 10717 | 10717 | 1 | 26 | Indel | 30.80% |
| T | 10716 | 10716 | 1 | 26 | Indel | 69.20% |
|  | 10716 | 10716 | 1 | 26 | Indel | 30.80% |
| T | 10715 | 10715 | 1 | 26 | Indel | 69.20% |
|  | 10715 | 10715 | 1 | 26 | Indel | 30.80% |
| A | 10714 | 10714 | 1 | 26 | Indel | 69.20% |
|  | 10714 | 10714 | 1 | 26 | Indel | 30.80% |
| T | 10713 | 10713 | 1 | 26 | Indel | 69.20% |
|  | 10713 | 10713 | 1 | 26 | Indel | 30.80% |
| A | 10712 | 10712 | 1 | 26 | Indel | 69.20% |
|  | 10712 | 10712 | 1 | 26 | Indel | 30.80% |
| G | 10711 | 10711 | 1 | 26 | Indel | 69.20% |
|  | 10711 | 10711 | 1 | 26 | Indel | 30.80% |
| A | 10710 | 10710 | 1 | 26 | Indel | 69.20% |
|  | 10710 | 10710 | 1 | 26 | Indel | 30.80% |
| A | 10709 | 10709 | 1 | 26 | Indel | 69.20% |
|  | 10709 | 10709 | 1 | 26 | Indel | 30.80% |
| A | 10708 | 10708 | 1 | 26 | Indel | 69.20% |
|  | 10708 | 10708 | 1 | 26 | Indel | 30.80% |
| T | 10707 | 10707 | 1 | 26 | Indel | 69.20% |
|  | 10707 | 10707 | 1 | 26 | Indel | 30.80% |
| A | 10706 | 10706 | 1 | 26 | Indel | 69.20% |
|  | 10706 | 10706 | 1 | 26 | Indel | 30.80% |
| T | 10682 | 10682 | 1 | 26 | Indel | 69.20% |
|  | 10682 | 10682 | 1 | 26 | Indel | 30.80% |
| A | 10681 | 10681 | 1 | 26 | Indel | 69.20% |
|  | 10681 | 10681 | 1 | 26 | Indel | 30.80% |
| A | 10680 | 10680 | 1 | 26 | Indel | 69.20% |
|  | 10680 | 10680 | 1 | 26 | Indel | 30.80% |
| T | 10679 | 10679 | 1 | 26 | Indel | 69.20% |
|  | 10679 | 10679 | 1 | 26 | Indel | 30.80% |
| C | 10678 | 10678 | 1 | 26 | Indel | 69.20% |
|  | 10678 | 10678 | 1 | 26 | Indel | 30.80% |
| T | 10677 | 10677 | 1 | 26 | Indel | 69.20% |
|  | 10677 | 10677 | 1 | 26 | Indel | 30.80% |
| T | 10676 | 10676 | 1 | 26 | Indel | 69.20% |
|  | 10676 | 10676 | 1 | 26 | Indel | 30.80% |
| A | 10675 | 10675 | 1 | 26 | Indel | 69.20% |
|  | 10675 | 10675 | 1 | 26 | Indel | 30.80% |
| A | 10674 | 10674 | 1 | 26 | Indel | 69.20% |
|  | 10674 | 10674 | 1 | 26 | Indel | 30.80% |
| A | 10673 | 10673 | 1 | 26 | Indel | 69.20% |
|  | 10673 | 10673 | 1 | 26 | Indel | 30.80% |
| T | 10672 | 10672 | 1 | 26 | Indel | 69.20% |
|  | 10672 | 10672 | 1 | 26 | Indel | 30.80% |
| C | 10671 | 10671 | 1 | 26 | Indel | 69.20% |
|  | 10671 | 10671 | 1 | 26 | Indel | 30.80% |
| T | 10670 | 10670 | 1 | 26 | Indel | 69.20% |
|  | 10670 | 10670 | 1 | 26 | Indel | 30.80% |
| T | 10669 | 10669 | 1 | 26 | Indel | 69.20% |
|  | 10669 | 10669 | 1 | 26 | Indel | 30.80% |
| A | 10668 | 10668 | 1 | 26 | Indel | 69.20% |
|  | 10668 | 10668 | 1 | 26 | Indel | 30.80% |
| T | 10667 | 10667 | 1 | 26 | Indel | 69.20% |
|  | 10667 | 10667 | 1 | 26 | Indel | 30.80% |
| A | 10666 | 10666 | 1 | 26 | Indel | 69.20% |
|  | 10666 | 10666 | 1 | 26 | Indel | 30.80% |
| C | 10610 | 10610 | 1 | 26 | Indel | 69.20% |
|  | 10610 | 10610 | 1 | 26 | Indel | 30.80% |
| T | 10561 | 10561 | 1 | 26 | Indel | 30.80% |
|  | 10561 | 10561 | 1 | 26 | Indel | 69.20% |
| A | 10560 | 10560 | 1 | 26 | Indel | 30.80% |
|  | 10560 | 10560 | 1 | 26 | Indel | 69.20% |
| T | 10559 | 10559 | 1 | 26 | Indel | 30.80% |
|  | 10559 | 10559 | 1 | 26 | Indel | 69.20% |
| A | 10558 | 10558 | 1 | 26 | Indel | 30.80% |
|  | 10558 | 10558 | 1 | 26 | Indel | 69.20% |
| A | 10557 | 10557 | 1 | 26 | Indel | 30.80% |
|  | 10557 | 10557 | 1 | 26 | Indel | 69.20% |
| A | 10556 | 10556 | 1 | 26 | Indel | 30.80% |
|  | 10556 | 10556 | 1 | 26 | Indel | 69.20% |
| T | 10555 | 10555 | 1 | 26 | Indel | 30.80% |
|  | 10555 | 10555 | 1 | 26 | Indel | 69.20% |
| A | 10554 | 10554 | 1 | 26 | Indel | 30.80% |
|  | 10554 | 10554 | 1 | 26 | Indel | 69.20% |
| A | 10553 | 10553 | 1 | 26 | Indel | 30.80% |
|  | 10553 | 10553 | 1 | 26 | Indel | 69.20% |
| T | 10552 | 10552 | 1 | 26 | Indel | 30.80% |
|  | 10552 | 10552 | 1 | 26 | Indel | 69.20% |
| A | 10551 | 10551 | 1 | 26 | Indel | 30.80% |
|  | 10551 | 10551 | 1 | 26 | Indel | 69.20% |
| A | 10550 | 10550 | 1 | 26 | Indel | 30.80% |
|  | 10550 | 10550 | 1 | 26 | Indel | 69.20% |
| A | 10549 | 10549 | 1 | 26 | Indel | 30.80% |
|  | 10549 | 10549 | 1 | 26 | Indel | 69.20% |
| T | 10548 | 10548 | 1 | 26 | Indel | 30.80% |
|  | 10548 | 10548 | 1 | 26 | Indel | 69.20% |
| A | 10547 | 10547 | 1 | 26 | Indel | 30.80% |
|  | 10547 | 10547 | 1 | 26 | Indel | 69.20% |
| T | 10546 | 10546 | 1 | 26 | Indel | 30.80% |
|  | 10546 | 10546 | 1 | 26 | Indel | 69.20% |
| A | 10545 | 10545 | 1 | 26 | Indel | 30.80% |
|  | 10545 | 10545 | 1 | 26 | Indel | 69.20% |
| T | 10521 | 10521 | 1 | 26 | Indel | 30.80% |
|  | 10521 | 10521 | 1 | 26 | Indel | 69.20% |
| T | 10510 | 10510 | 1 | 26 | Indel | 26.90% |
|  | 10510 | 10510 | 1 | 26 | Indel | 73.10% |
| A | 10509 | 10509 | 1 | 26 | Indel | 26.90% |
|  | 10509 | 10509 | 1 | 26 | Indel | 73.10% |
| A | 10508 | 10508 | 1 | 26 | Indel | 26.90% |
|  | 10508 | 10508 | 1 | 26 | Indel | 73.10% |
| A | 10507 | 10507 | 1 | 26 | Indel | 26.90% |
|  | 10507 | 10507 | 1 | 26 | Indel | 73.10% |
| A | 10506 | 10506 | 1 | 26 | Indel | 26.90% |
|  | 10506 | 10506 | 1 | 26 | Indel | 73.10% |
| T | 10505 | 10505 | 1 | 26 | Indel | 26.90% |
|  | 10505 | 10505 | 1 | 26 | Indel | 73.10% |
| A | 10504 | 10504 | 1 | 26 | Indel | 26.90% |
|  | 10504 | 10504 | 1 | 26 | Indel | 73.10% |
| A | 10503 | 10503 | 1 | 26 | Indel | 26.90% |
|  | 10503 | 10503 | 1 | 26 | Indel | 73.10% |
| T | 10502 | 10502 | 1 | 26 | Indel | 26.90% |
|  | 10502 | 10502 | 1 | 26 | Indel | 73.10% |
| A | 10501 | 10501 | 1 | 26 | Indel | 26.90% |
|  | 10501 | 10501 | 1 | 26 | Indel | 73.10% |
| A | 10500 | 10500 | 1 | 26 | Indel | 26.90% |
|  | 10500 | 10500 | 1 | 26 | Indel | 73.10% |
| A | 10499 | 10499 | 1 | 26 | Indel | 26.90% |
|  | 10499 | 10499 | 1 | 26 | Indel | 73.10% |
| T | 10498 | 10498 | 1 | 26 | Indel | 26.90% |
|  | 10498 | 10498 | 1 | 26 | Indel | 73.10% |
| A | 10497 | 10497 | 1 | 26 | Indel | 26.90% |
|  | 10497 | 10497 | 1 | 26 | Indel | 73.10% |
| T | 10496 | 10496 | 1 | 26 | Indel | 26.90% |
|  | 10496 | 10496 | 1 | 26 | Indel | 73.10% |
| A | 10495 | 10495 | 1 | 26 | Indel | 26.90% |
|  | 10495 | 10495 | 1 | 26 | Indel | 73.10% |
| A | 10494 | 10494 | 1 | 26 | Indel | 26.90% |
|  | 10494 | 10494 | 1 | 26 | Indel | 73.10% |
| T | 10493 | 10493 | 1 | 26 | Indel | 26.90% |
|  | 10493 | 10493 | 1 | 26 | Indel | 73.10% |
| T | 10492 | 10492 | 1 | 26 | Indel | 26.90% |
|  | 10492 | 10492 | 1 | 26 | Indel | 73.10% |
| A | 10491 | 10491 | 1 | 26 | Indel | 26.90% |
|  | 10491 | 10491 | 1 | 26 | Indel | 73.10% |
| A | 10490 | 10490 | 1 | 26 | Indel | 26.90% |
|  | 10490 | 10490 | 1 | 26 | Indel | 73.10% |
| T | 10489 | 10489 | 1 | 26 | Indel | 26.90% |
|  | 10489 | 10489 | 1 | 26 | Indel | 73.10% |
| A | 10488 | 10488 | 1 | 26 | Indel | 26.90% |
|  | 10488 | 10488 | 1 | 26 | Indel | 73.10% |
| A | 10487 | 10487 | 1 | 26 | Indel | 26.90% |
|  | 10487 | 10487 | 1 | 26 | Indel | 73.10% |
| A | 10486 | 10486 | 1 | 26 | Indel | 26.90% |
|  | 10486 | 10486 | 1 | 26 | Indel | 73.10% |
| A | 10485 | 10485 | 1 | 26 | Indel | 26.90% |
|  | 10485 | 10485 | 1 | 26 | Indel | 73.10% |
| T | 10484 | 10484 | 1 | 26 | Indel | 26.90% |
|  | 10484 | 10484 | 1 | 26 | Indel | 73.10% |
| A | 10483 | 10483 | 1 | 26 | Indel | 26.90% |
|  | 10483 | 10483 | 1 | 26 | Indel | 73.10% |
| A | 10482 | 10482 | 1 | 26 | Indel | 26.90% |
|  | 10482 | 10482 | 1 | 26 | Indel | 73.10% |
| T | 10481 | 10481 | 1 | 26 | Indel | 26.90% |
|  | 10481 | 10481 | 1 | 26 | Indel | 73.10% |
| A | 10480 | 10480 | 1 | 26 | Indel | 26.90% |
|  | 10480 | 10480 | 1 | 26 | Indel | 73.10% |
| A | 10479 | 10479 | 1 | 26 | Indel | 26.90% |
|  | 10479 | 10479 | 1 | 26 | Indel | 73.10% |
| A | 10478 | 10478 | 1 | 26 | Indel | 26.90% |
|  | 10478 | 10478 | 1 | 26 | Indel | 73.10% |
| A | 10477 | 10477 | 1 | 26 | Indel | 26.90% |
|  | 10477 | 10477 | 1 | 26 | Indel | 73.10% |
| T | 10476 | 10476 | 1 | 26 | Indel | 26.90% |
|  | 10476 | 10476 | 1 | 26 | Indel | 73.10% |
| T | 10475 | 10475 | 1 | 26 | Indel | 26.90% |
|  | 10475 | 10475 | 1 | 26 | Indel | 73.10% |
| A | 10474 | 10474 | 1 | 26 | Indel | 26.90% |
|  | 10474 | 10474 | 1 | 26 | Indel | 73.10% |
| A | 10473 | 10473 | 1 | 26 | Indel | 26.90% |
|  | 10473 | 10473 | 1 | 26 | Indel | 73.10% |
| T | 10472 | 10472 | 1 | 26 | Indel | 26.90% |
|  | 10472 | 10472 | 1 | 26 | Indel | 73.10% |
| A | 10471 | 10471 | 1 | 26 | Indel | 26.90% |
|  | 10471 | 10471 | 1 | 26 | Indel | 73.10% |
| A | 10470 | 10470 | 1 | 26 | Indel | 26.90% |
|  | 10470 | 10470 | 1 | 26 | Indel | 73.10% |
| A | 10469 | 10469 | 1 | 26 | Indel | 26.90% |
|  | 10469 | 10469 | 1 | 26 | Indel | 73.10% |
| A | 10468 | 10468 | 1 | 26 | Indel | 26.90% |
|  | 10468 | 10468 | 1 | 26 | Indel | 73.10% |
| T | 10467 | 10467 | 1 | 26 | Indel | 26.90% |
|  | 10467 | 10467 | 1 | 26 | Indel | 73.10% |
| A | 10466 | 10466 | 1 | 26 | Indel | 26.90% |
|  | 10466 | 10466 | 1 | 26 | Indel | 73.10% |
| A | 10465 | 10465 | 1 | 26 | Indel | 26.90% |
|  | 10465 | 10465 | 1 | 26 | Indel | 73.10% |
| T | 10464 | 10464 | 1 | 26 | Indel | 26.90% |
|  | 10464 | 10464 | 1 | 26 | Indel | 73.10% |
| A | 10463 | 10463 | 1 | 26 | Indel | 26.90% |
|  | 10463 | 10463 | 1 | 26 | Indel | 73.10% |
| A | 10462 | 10462 | 1 | 26 | Indel | 26.90% |
|  | 10462 | 10462 | 1 | 26 | Indel | 73.10% |
| A | 10461 | 10461 | 1 | 26 | Indel | 26.90% |
|  | 10461 | 10461 | 1 | 26 | Indel | 73.10% |
| T | 10460 | 10460 | 1 | 26 | Indel | 26.90% |
|  | 10460 | 10460 | 1 | 26 | Indel | 73.10% |
| A | 10459 | 10459 | 1 | 26 | Indel | 26.90% |
|  | 10459 | 10459 | 1 | 26 | Indel | 73.10% |
| T | 10458 | 10458 | 1 | 26 | Indel | 26.90% |
|  | 10458 | 10458 | 1 | 26 | Indel | 73.10% |
| A | 10457 | 10457 | 1 | 26 | Indel | 30.80% |
|  | 10457 | 10457 | 1 | 26 | Indel | 69.20% |
| A | 10456 | 10456 | 1 | 26 | Indel | 30.80% |
|  | 10456 | 10456 | 1 | 26 | Indel | 69.20% |
| T | 10455 | 10455 | 1 | 26 | Indel | 30.80% |
|  | 10455 | 10455 | 1 | 26 | Indel | 69.20% |
| A | 10454 | 10454 | 1 | 26 | Indel | 30.80% |
|  | 10454 | 10454 | 1 | 26 | Indel | 69.20% |
| T | 10453 | 10453 | 1 | 26 | Indel | 30.80% |
|  | 10453 | 10453 | 1 | 26 | Indel | 69.20% |
| A | 10452 | 10452 | 1 | 26 | Indel | 30.80% |
|  | 10452 | 10452 | 1 | 26 | Indel | 69.20% |
| T | 10451 | 10451 | 1 | 26 | Indel | 30.80% |
|  | 10451 | 10451 | 1 | 26 | Indel | 69.20% |
| A | 10450 | 10450 | 1 | 26 | Indel | 30.80% |
|  | 10450 | 10450 | 1 | 26 | Indel | 69.20% |
| T | 10449 | 10449 | 1 | 26 | Indel | 30.80% |
|  | 10449 | 10449 | 1 | 26 | Indel | 69.20% |
| T | 10448 | 10448 | 1 | 26 | Indel | 26.90% |
|  | 10448 | 10448 | 1 | 26 | Indel | 69.20% |
| A | 10447 | 10447 | 1 | 26 | Indel | 30.80% |
|  | 10447 | 10447 | 1 | 26 | Indel | 69.20% |
| A | 10446 | 10446 | 1 | 26 | Indel | 30.80% |
|  | 10446 | 10446 | 1 | 26 | Indel | 69.20% |
| T | 10445 | 10445 | 1 | 26 | Indel | 30.80% |
|  | 10445 | 10445 | 1 | 26 | Indel | 69.20% |
| A | 10444 | 10444 | 1 | 26 | Indel | 30.80% |
|  | 10444 | 10444 | 1 | 26 | Indel | 69.20% |
| A | 10443 | 10443 | 1 | 26 | Indel | 30.80% |
|  | 10443 | 10443 | 1 | 26 | Indel | 69.20% |
| A | 10442 | 10442 | 1 | 26 | Indel | 30.80% |
|  | 10442 | 10442 | 1 | 26 | Indel | 69.20% |
| T | 10441 | 10441 | 1 | 26 | Indel | 30.80% |
|  | 10441 | 10441 | 1 | 26 | Indel | 69.20% |
| A | 10440 | 10440 | 1 | 26 | Indel | 30.80% |
|  | 10440 | 10440 | 1 | 26 | Indel | 69.20% |
| T | 10439 | 10439 | 1 | 26 | Indel | 30.80% |
|  | 10439 | 10439 | 1 | 26 | Indel | 69.20% |
| G | 9983 | 9983 | 1 | 26 | Indel | 69.20% |
|  | 9983 | 9983 | 1 | 26 | Indel | 30.80% |
| A | 9982 | 9982 | 1 | 26 | Indel | 69.20% |
|  | 9982 | 9982 | 1 | 26 | Indel | 30.80% |
| A | 9981 | 9981 | 1 | 26 | Indel | 69.20% |
|  | 9981 | 9981 | 1 | 26 | Indel | 30.80% |
| T | 9980 | 9980 | 1 | 26 | Indel | 69.20% |
|  | 9980 | 9980 | 1 | 26 | Indel | 30.80% |
| A | 9979 | 9979 | 1 | 26 | Indel | 69.20% |
|  | 9979 | 9979 | 1 | 26 | Indel | 30.80% |
| A | 9978 | 9978 | 1 | 26 | Indel | 69.20% |
|  | 9978 | 9978 | 1 | 26 | Indel | 30.80% |
| A | 9977 | 9977 | 1 | 26 | Indel | 69.20% |
|  | 9977 | 9977 | 1 | 26 | Indel | 30.80% |
| G | 9976 | 9976 | 1 | 26 | Indel | 69.20% |
|  | 9976 | 9976 | 1 | 26 | Indel | 30.80% |
| T | 9945 | 9945 | 1 | 26 | Indel | 30.80% |
|  | 9945 | 9945 | 1 | 26 | Indel | 69.20% |
| T | 9944 | 9944 | 1 | 26 | Indel | 30.80% |
|  | 9944 | 9944 | 1 | 26 | Indel | 69.20% |
| T | 9943 | 9943 | 1 | 26 | Indel | 30.80% |
|  | 9943 | 9943 | 1 | 26 | Indel | 69.20% |
| T | 9942 | 9942 | 1 | 26 | Indel | 30.80% |
|  | 9942 | 9942 | 1 | 26 | Indel | 69.20% |
| C | 9775 | 9775 | 1 | 26 | Indel | 30.80% |
|  | 9775 | 9775 | 1 | 26 | Indel | 69.20% |
| T | 9435 | 9435 | 1 | 26 | Indel | 30.80% |
|  | 9435 | 9435 | 1 | 26 | Indel | 69.20% |
| T | 9434 | 9434 | 1 | 26 | Indel | 30.80% |
|  | 9434 | 9434 | 1 | 26 | Indel | 69.20% |
| A | 9433 | 9433 | 1 | 26 | Indel | 30.80% |
|  | 9433 | 9433 | 1 | 26 | Indel | 69.20% |
| A | 9432 | 9432 | 1 | 26 | Indel | 30.80% |
|  | 9432 | 9432 | 1 | 26 | Indel | 69.20% |
| T | 9431 | 9431 | 1 | 26 | Indel | 30.80% |
|  | 9431 | 9431 | 1 | 26 | Indel | 69.20% |
| G | 9430 | 9430 | 1 | 26 | Indel | 26.90% |
|  | 9430 | 9430 | 1 | 26 | Indel | 73.10% |
| A | 9429 | 9429 | 1 | 26 | Indel | 26.90% |
|  | 9429 | 9429 | 1 | 26 | Indel | 73.10% |
| G | 9428 | 9428 | 1 | 26 | Indel | 26.90% |
|  | 9428 | 9428 | 1 | 26 | Indel | 73.10% |
| T | 9427 | 9427 | 1 | 26 | Indel | 26.90% |
|  | 9427 | 9427 | 1 | 26 | Indel | 73.10% |
| A | 9426 | 9426 | 1 | 26 | Indel | 26.90% |
|  | 9426 | 9426 | 1 | 26 | Indel | 73.10% |
| T | 9425 | 9425 | 1 | 26 | Indel | 26.90% |
|  | 9425 | 9425 | 1 | 26 | Indel | 73.10% |
| G | 9424 | 9424 | 1 | 26 | Indel | 26.90% |
|  | 9424 | 9424 | 1 | 26 | Indel | 73.10% |
| A | 9423 | 9423 | 1 | 26 | Indel | 26.90% |
|  | 9423 | 9423 | 1 | 26 | Indel | 73.10% |
| A | 9422 | 9422 | 1 | 26 | Indel | 26.90% |
|  | 9422 | 9422 | 1 | 26 | Indel | 73.10% |
| T | 9421 | 9421 | 1 | 26 | Indel | 26.90% |
|  | 9421 | 9421 | 1 | 26 | Indel | 73.10% |
| A | 9420 | 9420 | 1 | 26 | Indel | 26.90% |
|  | 9420 | 9420 | 1 | 26 | Indel | 73.10% |
| A | 9419 | 9419 | 1 | 26 | Indel | 26.90% |
|  | 9419 | 9419 | 1 | 26 | Indel | 73.10% |
| C | 9418 | 9418 | 1 | 26 | Indel | 26.90% |
|  | 9418 | 9418 | 1 | 26 | Indel | 73.10% |
| T | 9385 | 9385 | 1 | 26 | Indel | 30.80% |
|  | 9385 | 9385 | 1 | 26 | Indel | 69.20% |
| T | 8661 | 8661 | 1 | 26 | Indel | 30.80% |
|  | 8661 | 8661 | 1 | 26 | Indel | 69.20% |
| A | 7953 | 7953 | 1 | 26 | Indel | 69.20% |
|  | 7953 | 7953 | 1 | 26 | Indel | 30.80% |
| A | 7218 | 7218 | 1 | 26 | Indel | 69.20% |
|  | 7218 | 7218 | 1 | 26 | Indel | 30.80% |
| A | 7217 | 7217 | 1 | 26 | Indel | 69.20% |
|  | 7217 | 7217 | 1 | 26 | Indel | 30.80% |
| A | 7216 | 7216 | 1 | 26 | Indel | 69.20% |
|  | 7216 | 7216 | 1 | 26 | Indel | 30.80% |
| T | 7215 | 7215 | 1 | 26 | Indel | 69.20% |
|  | 7215 | 7215 | 1 | 26 | Indel | 30.80% |
| A | 7214 | 7214 | 1 | 26 | Indel | 69.20% |
|  | 7214 | 7214 | 1 | 26 | Indel | 30.80% |
| A | 7213 | 7213 | 1 | 26 | Indel | 69.20% |
|  | 7213 | 7213 | 1 | 26 | Indel | 30.80% |
| T | 7212 | 7212 | 1 | 26 | Indel | 69.20% |
|  | 7212 | 7212 | 1 | 26 | Indel | 30.80% |
| T | 7211 | 7211 | 1 | 26 | Indel | 69.20% |
|  | 7211 | 7211 | 1 | 26 | Indel | 30.80% |
| A | 7210 | 7210 | 1 | 26 | Indel | 69.20% |
|  | 7210 | 7210 | 1 | 26 | Indel | 30.80% |
| G | 7209 | 7209 | 1 | 26 | Indel | 69.20% |
|  | 7209 | 7209 | 1 | 26 | Indel | 30.80% |
| A | 7208 | 7208 | 1 | 26 | Indel | 69.20% |
|  | 7208 | 7208 | 1 | 26 | Indel | 30.80% |
| T | 7207 | 7207 | 1 | 26 | Indel | 69.20% |
|  | 7207 | 7207 | 1 | 26 | Indel | 30.80% |
| A | 7206 | 7206 | 1 | 26 | Indel | 69.20% |
|  | 7206 | 7206 | 1 | 26 | Indel | 30.80% |
| T | 7205 | 7205 | 1 | 26 | Indel | 69.20% |
|  | 7205 | 7205 | 1 | 26 | Indel | 30.80% |
| T | 7204 | 7204 | 1 | 26 | Indel | 69.20% |
|  | 7204 | 7204 | 1 | 26 | Indel | 30.80% |
| A | 7203 | 7203 | 1 | 26 | Indel | 69.20% |
|  | 7203 | 7203 | 1 | 26 | Indel | 30.80% |
| A | 7064 | 7064 | 1 | 26 | Indel | 30.80% |
|  | 7064 | 7064 | 1 | 26 | Indel | 69.20% |
| A | 7063 | 7063 | 1 | 26 | Indel | 30.80% |
|  | 7063 | 7063 | 1 | 26 | Indel | 69.20% |
| A | 6906 | 6906 | 1 | 26 | Indel | 69.20% |
|  | 6906 | 6906 | 1 | 26 | Indel | 30.80% |
| C | 5726 | 5726 | 1 | 26 | Indel | 30.80% |
|  | 5726 | 5726 | 1 | 26 | Indel | 69.20% |
| C | 5725 | 5725 | 1 | 26 | Indel | 26.90% |
|  | 5725 | 5725 | 1 | 26 | Indel | 73.10% |
| C | 5570 | 5570 | 1 | 26 | Indel | 69.20% |
|  | 5570 | 5570 | 1 | 26 | Indel | 30.80% |
| C | 5569 | 5569 | 1 | 26 | Indel | 50.00% |
|  | 5569 | 5569 | 1 | 26 | Indel | 50.00% |
| T | 5218 | 5218 | 1 | 26 | Indel | 73.10% |
|  | 5218 | 5218 | 1 | 26 | Indel | 26.90% |
| A | 5217 | 5217 | 1 | 26 | Indel | 73.10% |
|  | 5217 | 5217 | 1 | 26 | Indel | 26.90% |
| T | 5216 | 5216 | 1 | 26 | Indel | 73.10% |
|  | 5216 | 5216 | 1 | 26 | Indel | 26.90% |
| A | 5215 | 5215 | 1 | 26 | Indel | 73.10% |
|  | 5215 | 5215 | 1 | 26 | Indel | 26.90% |
| G | 5214 | 5214 | 1 | 26 | Indel | 73.10% |
|  | 5214 | 5214 | 1 | 26 | Indel | 26.90% |
| A | 5213 | 5213 | 1 | 26 | Indel | 73.10% |
|  | 5213 | 5213 | 1 | 26 | Indel | 26.90% |
| T | 5212 | 5212 | 1 | 26 | Indel | 73.10% |
|  | 5212 | 5212 | 1 | 26 | Indel | 26.90% |
| A | 5211 | 5211 | 1 | 26 | Indel | 73.10% |
|  | 5211 | 5211 | 1 | 26 | Indel | 26.90% |
| T | 5210 | 5210 | 1 | 26 | Indel | 73.10% |
|  | 5210 | 5210 | 1 | 26 | Indel | 26.90% |
| A | 5209 | 5209 | 1 | 26 | Indel | 73.10% |
|  | 5209 | 5209 | 1 | 26 | Indel | 26.90% |
| G | 5208 | 5208 | 1 | 26 | Indel | 73.10% |
|  | 5208 | 5208 | 1 | 26 | Indel | 26.90% |
| T | 5207 | 5207 | 1 | 26 | Indel | 73.10% |
|  | 5207 | 5207 | 1 | 26 | Indel | 26.90% |
| A | 5206 | 5206 | 1 | 26 | Indel | 73.10% |
|  | 5206 | 5206 | 1 | 26 | Indel | 26.90% |
| T | 5205 | 5205 | 1 | 26 | Indel | 73.10% |
|  | 5205 | 5205 | 1 | 26 | Indel | 26.90% |
| G | 5204 | 5204 | 1 | 26 | Indel | 73.10% |
|  | 5204 | 5204 | 1 | 26 | Indel | 26.90% |
| A | 5203 | 5203 | 1 | 26 | Indel | 73.10% |
|  | 5203 | 5203 | 1 | 26 | Indel | 26.90% |
| T | 5202 | 5202 | 1 | 26 | Indel | 73.10% |
|  | 5202 | 5202 | 1 | 26 | Indel | 26.90% |
| T | 5201 | 5201 | 1 | 26 | Indel | 73.10% |
|  | 5201 | 5201 | 1 | 26 | Indel | 26.90% |
| T | 5200 | 5200 | 1 | 26 | Indel | 73.10% |
|  | 5200 | 5200 | 1 | 26 | Indel | 26.90% |
| A | 5199 | 5199 | 1 | 26 | Indel | 73.10% |
|  | 5199 | 5199 | 1 | 26 | Indel | 26.90% |
| A | 5198 | 5198 | 1 | 26 | Indel | 73.10% |
|  | 5198 | 5198 | 1 | 26 | Indel | 26.90% |
| C | 2015 | 2015 | 1 | 26 | Indel | 69.20% |
|  | 2015 | 2015 | 1 | 26 | Indel | 30.80% |
| T | 2014 | 2014 | 1 | 26 | Indel | 69.20% |
|  | 2014 | 2014 | 1 | 26 | Indel | 30.80% |
| C | 2013 | 2013 | 1 | 26 | Indel | 69.20% |
|  | 2013 | 2013 | 1 | 26 | Indel | 30.80% |
| T | 2012 | 2012 | 1 | 26 | Indel | 69.20% |
|  | 2012 | 2012 | 1 | 26 | Indel | 30.80% |
| A | 2011 | 2011 | 1 | 26 | Indel | 69.20% |
|  | 2011 | 2011 | 1 | 26 | Indel | 30.80% |
| T | 2010 | 2010 | 1 | 26 | Indel | 69.20% |
|  | 2010 | 2010 | 1 | 26 | Indel | 30.80% |
| T | 2009 | 2009 | 1 | 26 | Indel | 69.20% |
|  | 2009 | 2009 | 1 | 26 | Indel | 30.80% |
| G | 2008 | 2008 | 1 | 26 | Indel | 69.20% |
|  | 2008 | 2008 | 1 | 26 | Indel | 30.80% |
| T | 2007 | 2007 | 1 | 26 | Indel | 69.20% |
|  | 2007 | 2007 | 1 | 26 | Indel | 30.80% |
| T | 2006 | 2006 | 1 | 26 | Indel | 69.20% |
|  | 2006 | 2006 | 1 | 26 | Indel | 30.80% |
| T | 2005 | 2005 | 1 | 26 | Indel | 69.20% |
|  | 2005 | 2005 | 1 | 26 | Indel | 30.80% |
| T | 2004 | 2004 | 1 | 26 | Indel | 69.20% |
|  | 2004 | 2004 | 1 | 26 | Indel | 30.80% |
| T | 2003 | 2003 | 1 | 26 | Indel | 69.20% |
|  | 2003 | 2003 | 1 | 26 | Indel | 30.80% |
| T | 2002 | 2002 | 1 | 26 | Indel | 69.20% |
|  | 2002 | 2002 | 1 | 26 | Indel | 30.80% |
| A | 2001 | 2001 | 1 | 26 | Indel | 69.20% |
|  | 2001 | 2001 | 1 | 26 | Indel | 30.80% |
| A | 2000 | 2000 | 1 | 26 | Indel | 69.20% |
|  | 2000 | 2000 | 1 | 26 | Indel | 30.80% |
| T | 1999 | 1999 | 1 | 26 | Indel | 69.20% |
|  | 1999 | 1999 | 1 | 26 | Indel | 30.80% |
| T | 1998 | 1998 | 1 | 26 | Indel | 69.20% |
|  | 1998 | 1998 | 1 | 26 | Indel | 30.80% |
| A | 1701 | 1701 | 1 | 26 | Indel | 30.80% |
|  | 1701 | 1701 | 1 | 26 | Indel | 69.20% |
| A | 1700 | 1700 | 1 | 26 | Indel | 30.80% |
|  | 1700 | 1700 | 1 | 26 | Indel | 69.20% |
| A | 1699 | 1699 | 1 | 26 | Indel | 30.80% |
|  | 1699 | 1699 | 1 | 26 | Indel | 69.20% |
| T | 1698 | 1698 | 1 | 26 | Indel | 30.80% |
|  | 1698 | 1698 | 1 | 26 | Indel | 69.20% |
| A | 1697 | 1697 | 1 | 26 | Indel | 30.80% |
|  | 1697 | 1697 | 1 | 26 | Indel | 69.20% |
| A | 1696 | 1696 | 1 | 26 | Indel | 30.80% |
|  | 1696 | 1696 | 1 | 26 | Indel | 69.20% |
| G | 1695 | 1695 | 1 | 26 | Indel | 30.80% |
|  | 1695 | 1695 | 1 | 26 | Indel | 69.20% |
| A | 1694 | 1694 | 1 | 26 | Indel | 30.80% |
|  | 1694 | 1694 | 1 | 26 | Indel | 69.20% |
| T | 1693 | 1693 | 1 | 26 | Indel | 30.80% |
|  | 1693 | 1693 | 1 | 26 | Indel | 69.20% |
| T | 1692 | 1692 | 1 | 26 | Indel | 30.80% |
|  | 1692 | 1692 | 1 | 26 | Indel | 69.20% |
| T | 1691 | 1691 | 1 | 26 | Indel | 30.80% |
|  | 1691 | 1691 | 1 | 26 | Indel | 69.20% |
| T | 1690 | 1690 | 1 | 26 | Indel | 30.80% |
|  | 1690 | 1690 | 1 | 26 | Indel | 69.20% |
| T | 1689 | 1689 | 1 | 26 | Indel | 30.80% |
|  | 1689 | 1689 | 1 | 26 | Indel | 69.20% |
| T | 1688 | 1688 | 1 | 26 | Indel | 30.80% |
|  | 1688 | 1688 | 1 | 26 | Indel | 69.20% |
| T | 1687 | 1687 | 1 | 26 | Indel | 30.80% |
|  | 1687 | 1687 | 1 | 26 | Indel | 69.20% |
| T | 1686 | 1686 | 1 | 26 | Indel | 30.80% |
|  | 1686 | 1686 | 1 | 26 | Indel | 69.20% |
| T | 1685 | 1685 | 1 | 26 | Indel | 30.80% |
|  | 1685 | 1685 | 1 | 26 | Indel | 69.20% |
| T | 1684 | 1684 | 1 | 26 | Indel | 30.80% |
|  | 1684 | 1684 | 1 | 26 | Indel | 69.20% |
| T | 1683 | 1683 | 1 | 26 | Indel | 30.80% |
|  | 1683 | 1683 | 1 | 26 | Indel | 69.20% |
| A | 1682 | 1682 | 1 | 26 | Indel | 30.80% |
|  | 1682 | 1682 | 1 | 26 | Indel | 69.20% |
| A | 1681 | 1681 | 1 | 26 | Indel | 30.80% |
|  | 1681 | 1681 | 1 | 26 | Indel | 69.20% |
| G | 1680 | 1680 | 1 | 26 | Indel | 30.80% |
|  | 1680 | 1680 | 1 | 26 | Indel | 69.20% |
| T | 1679 | 1679 | 1 | 26 | Indel | 30.80% |
|  | 1679 | 1679 | 1 | 26 | Indel | 69.20% |
| A | 1678 | 1678 | 1 | 26 | Indel | 30.80% |
|  | 1678 | 1678 | 1 | 26 | Indel | 69.20% |
| A | 1677 | 1677 | 1 | 26 | Indel | 30.80% |
|  | 1677 | 1677 | 1 | 26 | Indel | 69.20% |
| A | 1676 | 1676 | 1 | 26 | Indel | 30.80% |
|  | 1676 | 1676 | 1 | 26 | Indel | 69.20% |
| G | 1675 | 1675 | 1 | 26 | Indel | 30.80% |
|  | 1675 | 1675 | 1 | 26 | Indel | 69.20% |
| T | 1674 | 1674 | 1 | 26 | Indel | 30.80% |
|  | 1674 | 1674 | 1 | 26 | Indel | 69.20% |
| T | 333 | 333 | 1 | 26 | Indel | 30.80% |
|  | 333 | 333 | 1 | 26 | Indel | 69.20% |
| G | 332 | 332 | 1 | 26 | Indel | 30.80% |
|  | 332 | 332 | 1 | 26 | Indel | 69.20% |
| A | 331 | 331 | 1 | 26 | Indel | 30.80% |
|  | 331 | 331 | 1 | 26 | Indel | 69.20% |
| T | 330 | 330 | 1 | 26 | Indel | 30.80% |
|  | 330 | 330 | 1 | 26 | Indel | 69.20% |
| A | 329 | 329 | 1 | 26 | Indel | 30.80% |
|  | 329 | 329 | 1 | 26 | Indel | 69.20% |
| A | 328 | 328 | 1 | 26 | Indel | 30.80% |
|  | 328 | 328 | 1 | 26 | Indel | 69.20% |
| C | 281 | 281 | 1 | 26 | Indel | 30.80% |
|  | 281 | 281 | 1 | 26 | Indel | 69.20% |
| A | 280 | 280 | 1 | 26 | Indel | 30.80% |
|  | 280 | 280 | 1 | 26 | Indel | 69.20% |
| A | 279 | 279 | 1 | 26 | Indel | 30.80% |
|  | 279 | 279 | 1 | 26 | Indel | 69.20% |
| A | 278 | 278 | 1 | 26 | Indel | 30.80% |
|  | 278 | 278 | 1 | 26 | Indel | 69.20% |
| A | 277 | 277 | 1 | 26 | Indel | 30.80% |
|  | 277 | 277 | 1 | 26 | Indel | 69.20% |
| T | 276 | 276 | 1 | 26 | Indel | 30.80% |
|  | 276 | 276 | 1 | 26 | Indel | 69.20% |
| A | 275 | 275 | 1 | 26 | Indel | 30.80% |
|  | 275 | 275 | 1 | 26 | Indel | 69.20% |
| A | 274 | 274 | 1 | 26 | Indel | 30.80% |
|  | 274 | 274 | 1 | 26 | Indel | 69.20% |
| G | 273 | 273 | 1 | 26 | Indel | 30.80% |
|  | 273 | 273 | 1 | 26 | Indel | 69.20% |
| A | 272 | 272 | 1 | 26 | Indel | 30.80% |
|  | 272 | 272 | 1 | 26 | Indel | 69.20% |
| A | 271 | 271 | 1 | 26 | Indel | 30.80% |
|  | 271 | 271 | 1 | 26 | Indel | 69.20% |
| T | 82 | 82 | 1 | 26 | Indel | 30.80% |
|  | 82 | 82 | 1 | 26 | Indel | 69.20% |
| T | 81 | 81 | 1 | 26 | Indel | 30.80% |
|  | 81 | 81 | 1 | 26 | Indel | 69.20% |
| T | 80 | 80 | 1 | 26 | Indel | 30.80% |
|  | 80 | 80 | 1 | 26 | Indel | 69.20% |
| A | 79 | 79 | 1 | 26 | Indel | 30.80% |
|  | 79 | 79 | 1 | 26 | Indel | 69.20% |
| T | 78 | 78 | 1 | 26 | Indel | 30.80% |
|  | 78 | 78 | 1 | 26 | Indel | 69.20% |
| A | 77 | 77 | 1 | 26 | Indel | 30.80% |
|  | 77 | 77 | 1 | 26 | Indel | 69.20% |
| A | 76 | 76 | 1 | 26 | Indel | 30.80% |
|  | 76 | 76 | 1 | 26 | Indel | 69.20% |
| T | 75 | 75 | 1 | 26 | Indel | 30.80% |
|  | 75 | 75 | 1 | 26 | Indel | 69.20% |
| T | 74 | 74 | 1 | 26 | Indel | 30.80% |
|  | 74 | 74 | 1 | 26 | Indel | 69.20% |
| T | 73 | 73 | 1 | 26 | Indel | 30.80% |
|  | 73 | 73 | 1 | 26 | Indel | 69.20% |
| A | 72 | 72 | 1 | 26 | Indel | 30.80% |
|  | 72 | 72 | 1 | 26 | Indel | 69.20% |
| T | 71 | 71 | 1 | 26 | Indel | 30.80% |
|  | 71 | 71 | 1 | 26 | Indel | 69.20% |
| A | 70 | 70 | 1 | 26 | Indel | 30.80% |
|  | 70 | 70 | 1 | 26 | Indel | 69.20% |
| T | 69 | 69 | 1 | 26 | Indel | 30.80% |
|  | 69 | 69 | 1 | 26 | Indel | 69.20% |
| A | 44 | 44 | 1 | 26 | Indel | 30.80% |
|  | 44 | 44 | 1 | 26 | Indel | 69.20% |
| T | 43 | 43 | 1 | 26 | Indel | 30.80% |
|  | 43 | 43 | 1 | 26 | Indel | 69.20% |
| A | 42 | 42 | 1 | 26 | Indel | 30.80% |
|  | 42 | 42 | 1 | 26 | Indel | 69.20% |
| A | 41 | 41 | 1 | 26 | Indel | 30.80% |
|  | 41 | 41 | 1 | 26 | Indel | 69.20% |
| T | 40 | 40 | 1 | 26 | Indel | 30.80% |
|  | 40 | 40 | 1 | 26 | Indel | 69.20% |
| T | 39 | 39 | 1 | 26 | Indel | 30.80% |
|  | 39 | 39 | 1 | 26 | Indel | 69.20% |
